# Accelerating Whole-Cell Simulations of mRNA Translation Using a Dedicated Hardware

**DOI:** 10.1021/acssynbio.1c00415

**Published:** 2021-11-23

**Authors:** David Shallom, Danny Naiger, Shlomo Weiss, Tamir Tuller

**Affiliations:** † School of Electrical Engineering, Tel Aviv University, Tel Aviv 69978, Israel; ‡ Department of Biomedical Engineering, Tel Aviv University, Tel Aviv 69978, Israel

**Keywords:** mRNA translation, FPGA, gene expression optimization, TASEP, hardware acceleration, whole-cell translation simulation

## Abstract

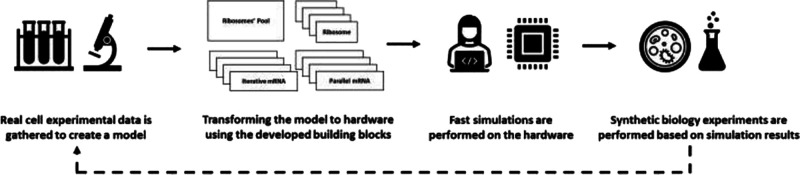

In recent years, intracellular biophysical simulations have been used with increasing frequency not only for answering basic scientific questions but also in the field of synthetic biology. However, since these models include networks of interaction between millions of components, they are extremely time-consuming and cannot run easily on parallel computers. In this study, we demonstrate for the first time a novel approach addressing this challenge by using a dedicated hardware designed specifically to simulate such processes. As a proof of concept, we specifically focus on mRNA translation, which is the process consuming most of the energy in the cell. We design a hardware that simulates translation in *Escherichia coli* and *Saccharomyces cerevisiae* for thousands of mRNAs and ribosomes, which is in orders of magnitude faster than a similar software solution. With the sharp increase in the amount of genomic data available today and the complexity of the corresponding models inferred from them, we believe that the strategy suggested here will become common and can be used among others for simulating entire cells with all gene expression steps.

## Introduction

Biophysical models of intracellular processes such as gene expression have been used in recent years for studying numerous questions related to all biomedical disciplines.^1−8^ The more advanced models in the field consider the “competition” of molecules in the cell (e.g., mRNAs) on resources (e.g., ribosomes).^8−11^ In recent years, we understand that without considering this aspect, the models usually provide significantly biased prediction and miss important intracellular aspects.^6−9,11−19^ Thus, it is clear that in the near future, these models will be very frequently used for synthetic biology for designing cells and viruses; indeed, recent manuscripts emphasize the importance of such whole-cell simulations in synthetic biology.^20−23^ However, when performing designs based on such models, the running time is orders of magnitude lower than just predicting a single intracellular stage. Thus, our approach is needed.

As a case study, we focus here on mRNA translation. Since a typical cell includes thousands of mRNAs and ribosomes, the simulation of such a process is computationally challenging and cannot be parallelized easily. As the state of each mRNA molecule depends on the global assignment of ribosomes to mRNA molecules, software simulating mRNA molecules should operate in a synchronized manner. That enforces large synchronization overheads that, in turn, will also degrade the performance of equivalent graphical processing unit (GPU) implementations that typically accommodate large amounts of parallel threads.

This is specifically challenging when various sets of parameters of the models are studied or optimized as in the case of synthetic biology, where the aim is to find a set of modifications in the cell that will optimize a certain objective that is affected by a large pool of factors in the cell.^7,9,11,19,21,24−27^ In such cases, the optimizations can easily take many months or even years.

In this study, we demonstrate for the first time a new approach for tackling this challenge based on the design of a dedicated hardware that can yield an optimization process that is orders of magnitude faster. There exist a few previous studies describing very small analogous circuits^28−33^ that are inspired by biological phenomena and can capture many biological effects. However, no previous study included digital parallel whole-cell dedicated hardware.

**Figure 1:**
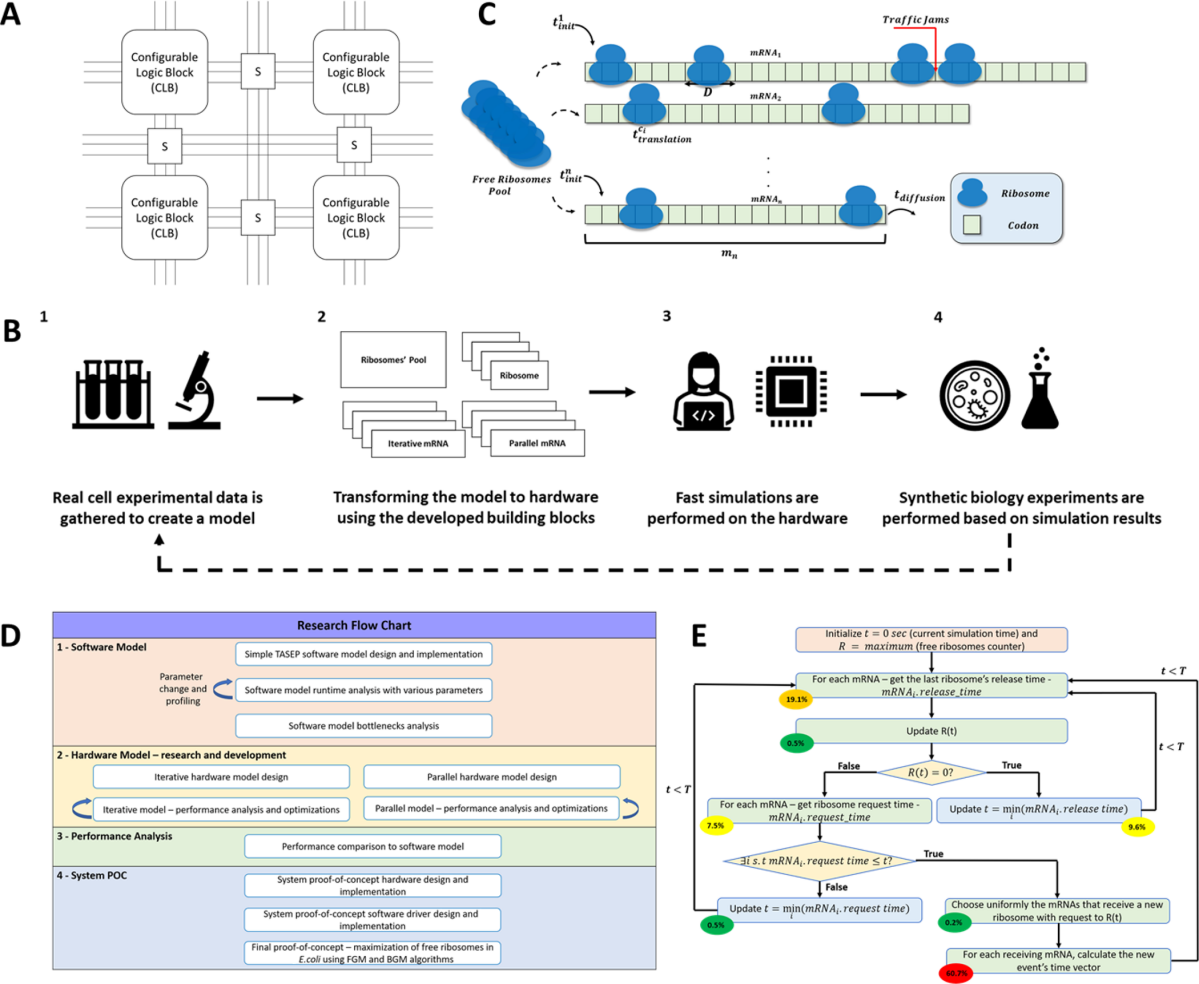
(A) Schematic high-level diagram of an FPGA. It consists of configurable logic blocks that are connected via the switching matrices (S). (B) Flow graph of the suggested iterations using the hardware model. (C**)** Schematic illustration for the biophysical process of mRNA translation. *t*_init_^*i*^denotes the average time it takes a ribosome to attach to a specific mRNA molecule, *t*_translation_^*c_i_*^ denotes the translation time of codon *c* on the *i*th mRNA, *t*_diffusion_ denotes the diffusion time of the ribosome after finishing translation, and *m_i_* denotes the length of the *i*th mRNA. (D) High-level flow diagram of the research flow. (E) High-level flow graph of the software model. Profiling results of the different stages is also shown next to each node in percentage out of total runtime. *T* denotes the end time of the simulation in real cell time.

In this work, we design a dedicated hardware using an FPGA (field-programmable gate array). FPGAs are configurable chips that can accommodate large user-designed digital circuits. FPGAs are often used for prototyping hardware designs before producing full dedicated ASICs (application-specific integrated circuits). An FPGA chip usually consists of a two-dimensional array of configurable logic blocks (memories, logic gates, multiplexers, etc.) alongside programmable interconnections that can connect logic blocks (see Figure 1A). The development of FPGA hardware consists of writing HDL (hardware description language) code and utilizing the FPGA’s vendor tool chain to translate the HDL to the FPGA configuration.

The general flow diagram of the suggested approach is described in Figure 1B. We first design the simulative model based on large-scale experimental biological measurements (e.g., coding sequences, mRNA levels, ribosomal densities, etc.). We then transform the simulative model to hardware by combining the building blocks described in this paper. Consecutive fast runs of the model in hardware with various parameters will follow, and finally, based on the results, synthetic biology experiments are performed. The entire process can then be repeated based on new experimental observations.

## Results

To demonstrate our approach, we focus here on a basic whole-cell simulation of mRNA translation. The model includes all the basic aspects of the computational models previously used in the field^7^ in addition to the ribosomes’ diffusion property. Specifically, our model includes the following aspects (see Figure 1C for illustration):

(1) Different translation time for each codon, which is related to the local biophysical properties of the mRNA and their interactions with translation factors and/or the availability of translation factors (e.g., tRNA levels).
(2) Initiation rates (which are affected by the properties of the mRNAs and initiation factors and global factors such as the concentrations of ribosomes and translation factors).
(3) More than one ribosome can translate an mRNA at a certain time.
(4) A single ribosome occupies several codons when moving alongside the mRNA molecule (due to its size). Therefore, several ribosomes alongside the same mRNA are forced to keep a minimal distance from each other.
(5) The movement is directional from the 5′ to the 3′ end of the mRNA.
(6) Different mRNA molecules compete for the same pool of ribosomes, i.e., a limited resource of ribosomes exists.
(7) Diffusion time—the time it takes for each ribosome to become available for translation after completing a translation of an mRNA molecule.

Our main test case for this research is the bacterium *Escherichia coli*. We specifically used all the genomic information related to real *E. coli* cells (see more details in the Methods section).

The research flow is described in Figure 1D. We first design, implement, and analyze an equivalent software model. Then, in accordance with the performance bottlenecks observed in the software model, we tackle the challenge of designing a dedicated hardware model using two architectural approaches. Next, we analyze the results and proceed to a whole-system proof-of-concept. The proof-of-concept includes variants of the algorithms presented in ref (22) for ribosomal traffic jam optimization. Finally, we analyze the results and suggest some future research directions.

### Equivalent Software Model Design

The software implementation consists of a list of mRNA objects that is addressed via a global scheduler that controls the global ribosomes’ pool and keeps track of the allocated and released ribosomes. Each mRNA object should only keep track of the times (in milliseconds) in which the previous ribosome finished initialization, codon translations, and diffusion (the “time-event vector”). When receiving a ribosome from the global scheduler, the above list of times can be calculated only by using the mRNA translation delays and the previous ribosome time-event vector (see the Methods section for a pseudo code). Then, the global scheduler keeps track of the generated time vectors of all mRNAs to determine the exact times in which ribosomes are allocated to specific mRNAs, ribosomes are freed, and proteins are generated. The high-level flow of the software model is shown in Figure 1E.

### Analyzing the Software Model

**Figure 2:**
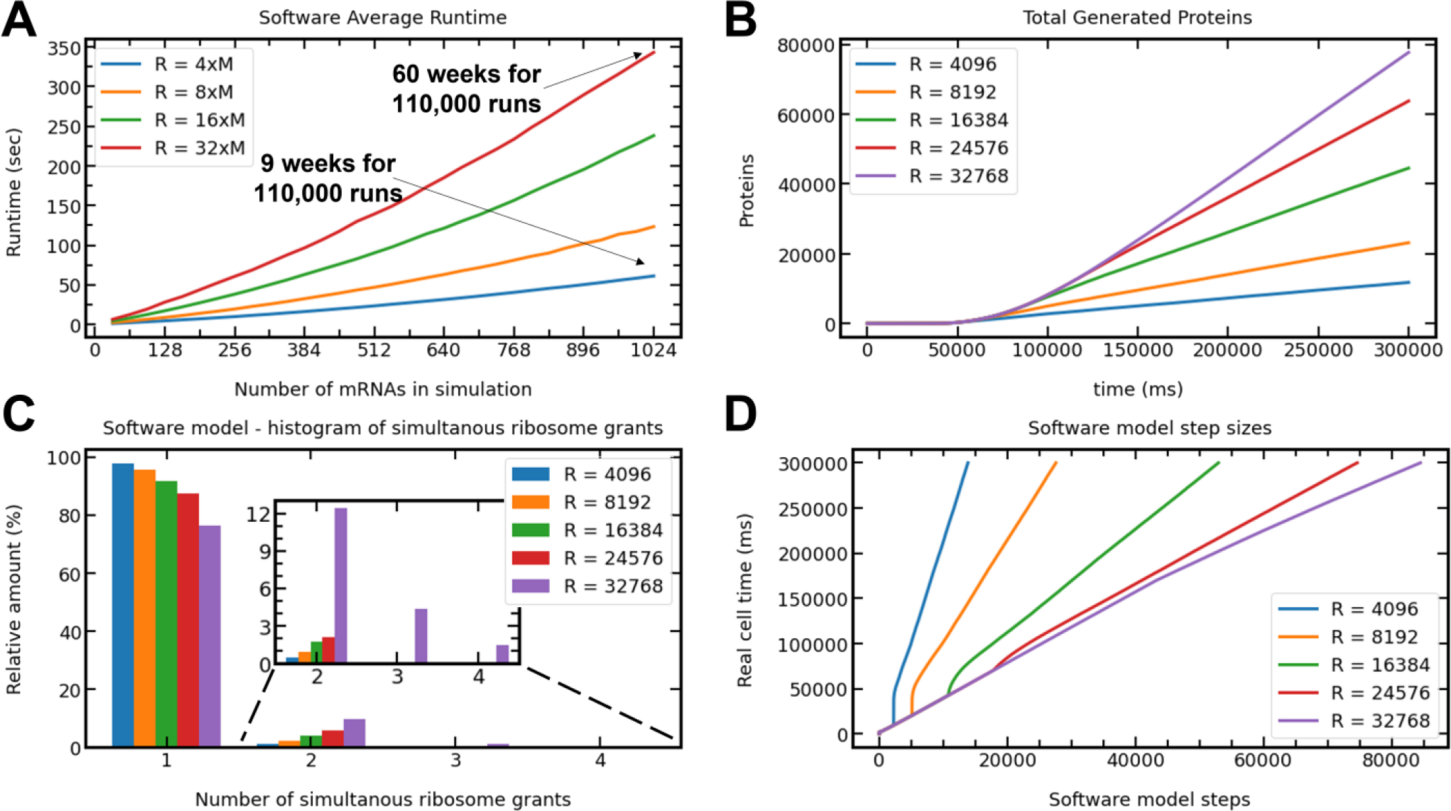
Software model analysis. (A) The runtime in seconds of the software model for 20 min of a real cell for various numbers of mRNAs and ribosomes. *R* denotes the number of ribosomes in the cell and *M* denotes the number of mRNAs. (B) The total amount of generated proteins as a function of time. As expected, we get more proteins as we increase the number of ribosomes in the cell. (C) The distribution of simultaneous ribosome grants in simulation. Ribosome grants are the events in which some ribosomes are released to the global pool and the arbiter decides which mRNA molecule is going to receive them. For example, for 32,768 ribosomes, we get one ribosomal grant in each iteration in around 80% of the iterations. (D) The number of iterations required by the model. As shown, all graphs end at the same time and take different numbers of iterations to complete.

The software model runtime in seconds as a function of mRNAs and ribosome numbers for 20 min of real cell time is shown in Figure 2.A. The percentage values depicted in Figure 1E are based on profiling the software model.

As can be seen in Figure 1E, the most time-consuming task is the calculation of the time-event vector upon granting a new ribosome to an mRNA. This calculation is responsible for calculating the exact times in which the new ribosome finishes translating each codon. In theory, we can reach this state with several ribosome attachments simultaneously. The time vector calculation for all the simultaneous grants can be carried in a parallel manner to reduce execution time. In Figure 2C, we can see that simultaneous grants are very rare, so in practice, we will not get significant improvement by parallelizing this part (i.e., using more cores to execute separate calls to the time-event vector calculation function). Also, we noticed that as the number of ribosomes in the cell grows, simultaneous grants are more probable (but still negligible).

Also, the calculation of the time-event vector itself is highly sequential due to its cumulative nature and the dependency in the previous ribosome time-vector. That is, the time-vector calculation itself also cannot be easily parallelized.

This implies that the main bottleneck of the software model is the mRNA synchronization stage—the simulation can advance only after calculating the state of all mRNA molecules. Thus, implementing the entire model in FPGA can accelerate this stage as FPGA is suitable for accommodating large amounts of synchronized replicas of hardware instances.

Also, note that the Δ*t* between consecutive global while iterations are determined by the fastest event (release/request of a ribosome). If those events happen at a similar time on all mRNAs, the simulation can be rather effective—it advances in large steps instead of waiting for the delay times (as done in our hardware equivalent model as shown later). Since this, in general, is not the case in real cells with various mRNA lengths, initiation times, diffusion times, and codon delays, the software simulation suffers greatly from the synchronization overhead (see Figure 2D for the timing of the simulation steps).

Moreover, as shown in Figure 2A, the average runtime of the software model grows as the number of ribosomes and mRNAs grows.

We show that, by implementing the optimization algorithms in ref (22) for more mRNA molecules, we needed approximately 110,000 runs of the model with various parameters. According to the profiling results, using the software model, it would take from 2.2 months (for 1024 mRNAs and 4096 ribosomes) up to 1 year and 3 months (for 1024 mRNAs and 32,768 ribosomes).

### High-Level Hardware Architecture

**Figure 3:**
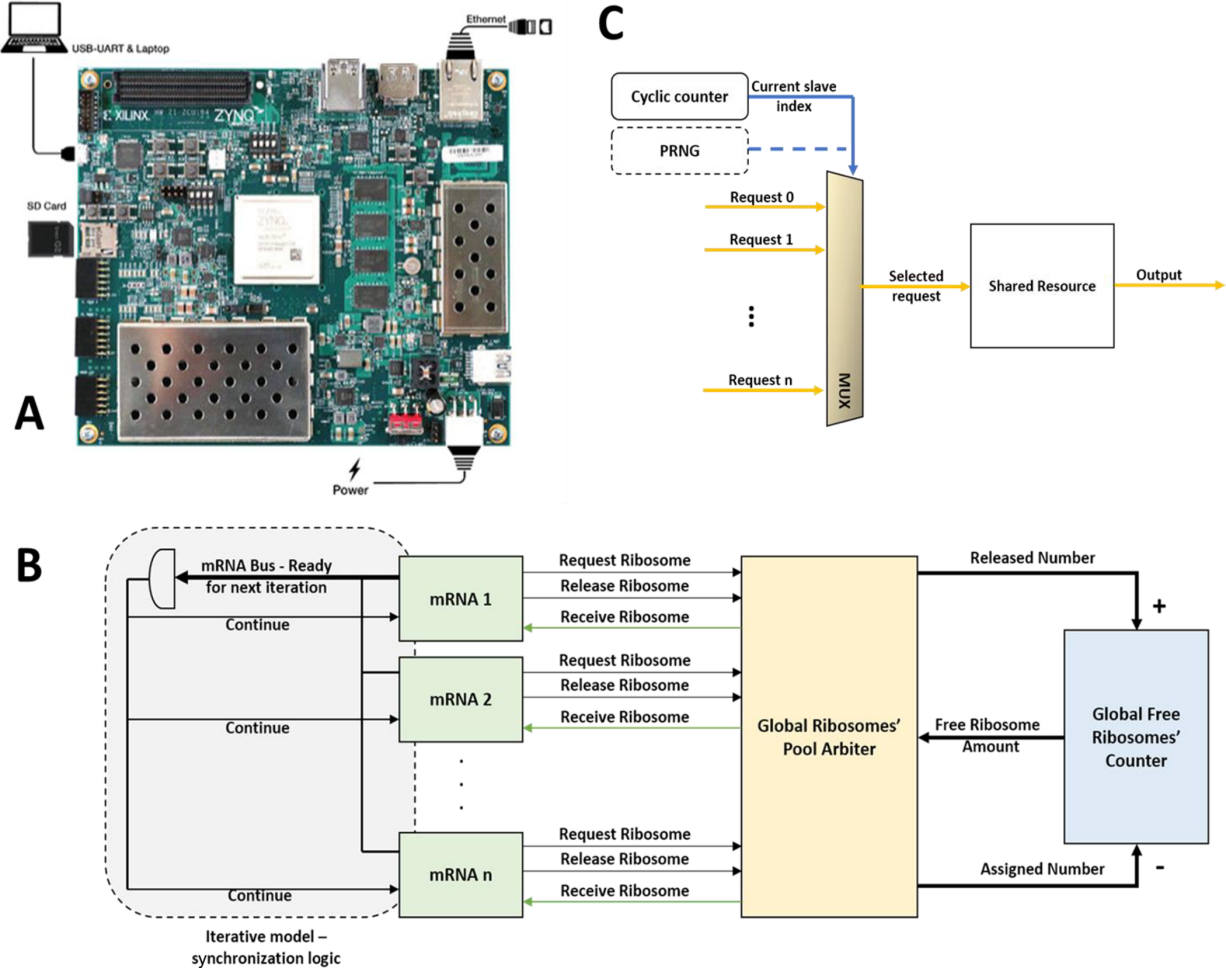
Hardware overview and design. (A) Xilinx ZCU104 evaluation board containing a Zynq Ultrascale+ FPGA chip. In the system POC, the firmware is loaded to the SD card, and the model is accessed via an ethernet connection. (B) High-level block diagram of the hardware models. The dashed lined components are for synchronizing the mRNA modules in the iterative model and are not present in the parallel model. (C) Schematic of a basic round-robin arbiter. In dashed line: By replacing the cyclic counter with a pseudorandom number generator (PRNG), we can get a uniform arbiter.

In this work, we are using the Xilinx ZCU104 evaluation board (see Figure 3A) that consists of a Zynq chip containing several CPU (central processing unit) cores alongside an FPGA.

We first divided the overall architecture design to several building blocks that should be designed (see Figure 3B). These include:

(1) mRNA module – Hardware entity that contains the specific codons’ information, manages the ribosomes, and keeps track of the generated proteins.
(2) Global arbiter – Hardware entity that is responsible for managing the ribosomes’ assignment to different mRNAs and keeps track of the overall ribosomes’ counter.
(3) Ribosomes – Hardware entity that keeps track of the state of the ribosomes (current codon index, translation time, etc.).

Also, to model allocation, translation, and diffusion delays, we use timers that are decremented in each clock cycle. The initialization value of those timers is chosen by normalizing all delays from seconds to clock cycles. In *E. coli*, for instance, each timer decrement models 1 ms in “real” cell time. Therefore, when running the hardware with input clock frequency *f*, the timer’s decrement phase of the model can potentially take  times faster in hardware. Thus, for example, for *f* = 100 MHz, the hardware runs 100,000 times faster (in the decrement phase) than a real cell.

### Ribosomes’ Global Arbiter

We choose an approach where one global entity keeps track of the available ribosomes. As shown in Figure 3B, this entity receives the request from and releases signals to all mRNAs, updates the free ribosome pool, and grants ribosomes (if available) to the requesting mRNAs.

We also examined an alternative approach in which we did not keep a global entity that manages the ribosomes but a small local buffer of ribosomes that propagates in a concatenated manner between the mRNA molecules. This method introduced considerable bias in the ribosomal allocation probability (see the Supporting Information and Figures S1, S7, and S9 for more details).

The required arbiter here is quite different from the common arbiters used today in hardware implementations^34−40^ for the following reasons:

(1) The arbitration is done among lots of endpoints (potentially hundreds to thousands)—this depends on the number of the mRNA molecules that we were able to fit into the device.
(2) The arbitration is done with uniform probability—each requesting endpoint (mRNA) should receive the resource (ribosome) with equal probability.

We note that existing implementations of hardware arbiters are designed for only few endpoints (i.e., multiple cores accessing the same shared memory) and are not required to be strictly uniform. In this work, we examined two implementations for the global arbiter—the “round-robin arbiter” and the “uniform arbiter.” The first is examined since it is commonly used in hardware implementations, and the second is our own variation that fixes the bias caused by the first.

### Round-Robin Arbiter

For simplicity, we first examined a basic round-robin arbiter, which is deterministic and therefore does not necessarily satisfy item (2) above. The round-robin arbiter sequentially examines the mRNAs, grants a ribosome if requested (and if a ribosome is freely available), and updates the pool’s counter with respect to the mRNA release signals (see Figure 3C).

**Figure 4:**
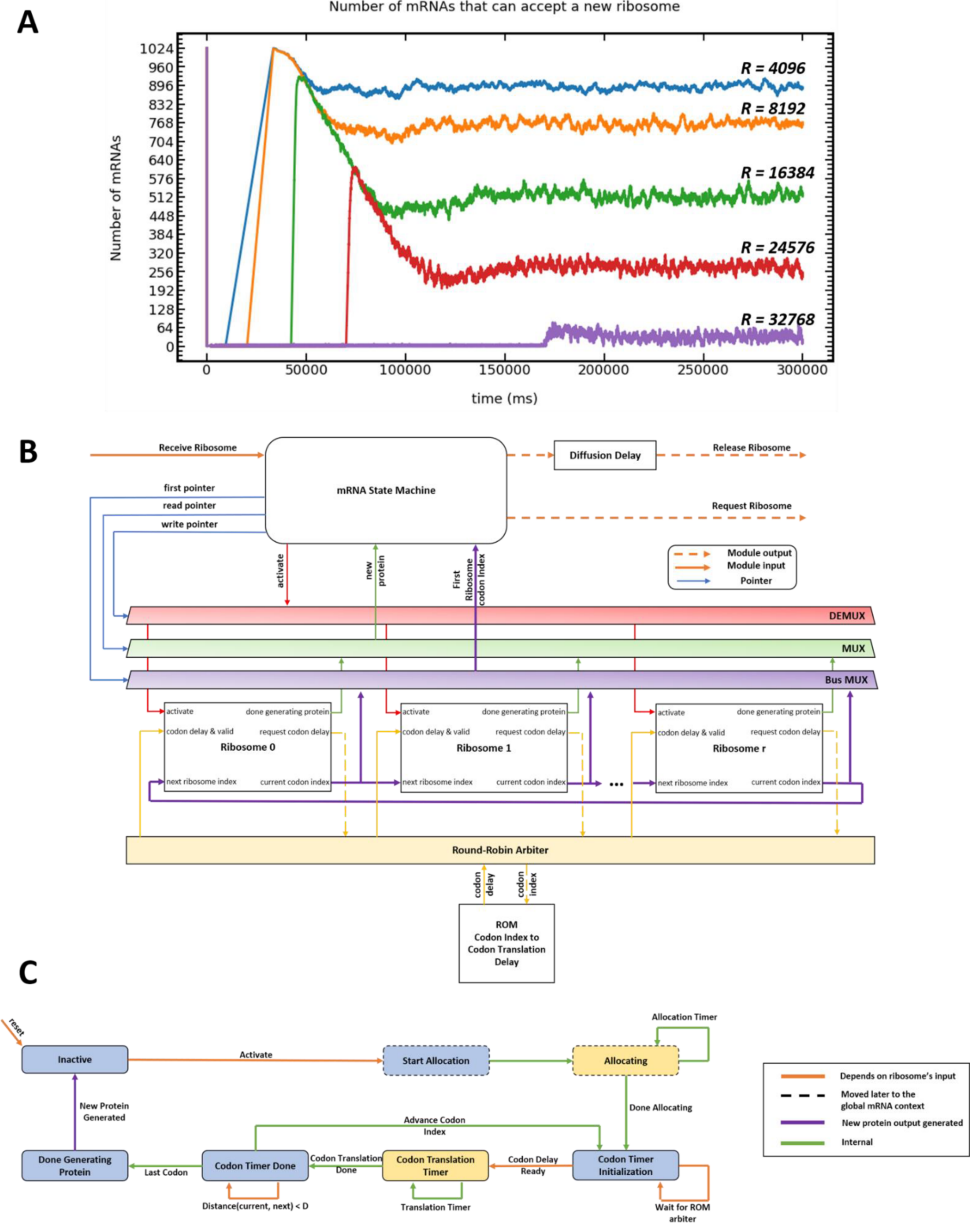
Arbiter’s hit probability and the parallel mRNA module. (A) The amount of mRNA molecules that are free to receive new ribosomes in the steady state for various ribosome amounts. (B) Block diagram of the parallel mRNA module and its local allocated hardware ribosomes. (C) Hardware ribosome’s state machine. The dashed-line states are later moved to the global mRNA context.

Figure 4A depicts the number of mRNAs that are free to receive new ribosomes as a function of time. As can be noticed for low and intermediate levels of ribosomes, the probability that a given mRNA is free to receive a ribosome is quite high. Therefore, since in this case, most of the mRNAs can receive new ribosomes in a steady state, it is highly probable that when the arbiter releases a ribosome from the *i*th mRNA, it will be collected by the consecutive (*i* + 1)th mRNA. This, of course, introduces a bias (see the Supporting Information and Figure S9 for more details).

On the other hand, for cases in which the cell is saturated with ribosomes, we see that the number of free mRNAs reduces substantially. Therefore, in those cases, the bias that the round-robin arbiter introduces is smaller.

### Uniform Arbiter

The round-robin arbiter consists of a cyclic counter that iterates sequentially over all mRNA indices. This counter is what causes the bias discussed above for simulations with low and intermediate numbers of ribosomes. Therefore, we decided to replace the counter by a hardware-efficient uniform pseudorandom number generator (UPRNG; see Figure 3C). By doing so, the arbiter first randomizes an index and then examines the signals of the indexed mRNA. This arbiter randomizes an index regardless of the state of the corresponding mRNA (requesting a ribosome or not). From Figure 4A, we can see that the arbiter’s hit probability (the probability to hit a free mRNA) is quite high for low and intermediate cell ribosomes’ number (which fits the typical physiological conditions^41^). For lower hit probabilities that occur in highly saturated cells, it might take the arbiter a long time until it reaches a free mRNA.

We will show that even for low hit probabilities, this arbiter is quite accurate. To understand that, we first need to introduce the mRNA modules’ architecture.

### Iterative Versus Parallel Hardware mRNA Model Approaches

For the design of the mRNA module, we examined two common hardware complementary design approaches:

(1) Parallel hardware design approach – Using as many replicas of processing units as necessary to improve timing performance. This approach typically results with high chip area consumption and high throughput.
(2) Iterative hardware design approach – Employing the same processing unit for different workloads if possible. This approach typically results with low chip-area consumption but also with lower throughput.

While the parallel approach might greatly improve the overall performance with respect to the software implementation, it might not support as many mRNAs as we need in a single FPGA chip. Next, we examine both approaches.

### Parallel Hardware mRNA Module

In the parallel approach, we wish to have all mRNAs and ribosomes run as autonomously as possible as if they were real molecules operating in a living cell. The major difficulty introduced by this approach is the assignment of ribosomes to mRNA molecules.

Here, each mRNA molecule contains a concatenated structure of hardware-implemented ribosomes as shown in Figure 4B (see the Supporting Information for more information).

Each mRNA molecule has a static allocation of hardware ribosomes. The hardware ribosomes start inactive and are activated one by one when an mRNA receives new ribosomes from the global arbiter. For managing the hardware ribosomes (i.e., activation and release), the mRNA state machine manages the following pointers:

(1) Write pointer – The index of the next inactive hardware ribosome. When the mRNA receives a new ribosome from the arbiter, the hardware ribosome pointed by this pointer is activated.
(2) First pointer – The index of the last hardware ribosome that was activated. That ribosome is the closest to the 5′ end of the mRNA. Using this pointer, the mRNA state machine can monitor the first ribosome’s index until it is far enough along the mRNA for it to be able to accept a new ribosome.
(3) Read pointer – The index of the ribosome that is closest to the 3′ end of the mRNA. The ribosome pointed by this pointer will be the next to generate a protein and then is deactivated.

The concatenation structure of the hardware-ribosomes resembles the internal implementation of memory-based first-in first-out (FIFO), which similarly consists of read and write pointers with similar functionality.

Instead of maintaining a local copy of the mRNA delays’ table for each hardware ribosome, we used a round-robin arbiter and a single read-only memory (ROM) for all hardware ribosomes of the same mRNA. That is important because, as shown later, the hardware utilization of each ribosome is the main limiting factor in the global utilization of the FPGA’s resources. The round-robin arbiter sequentially reads the current index of each ribosome and posts the codon delay from the table.

The diffusion of the ribosomes (i.e., the time it takes a ribosome to be usable again by other mRNAs) is modeled by delaying the “released” signal of the mRNA molecule.

### Hardware Ribosome Module

Each ribosome consists of the state machine as illustrated in Figure 4C. Here, the delay that the state machine adds to the ribosome timing (including waiting for the ROM arbiter) is negligible in relation to the timer’s delays.

As the hardware ribosome module is instantiated multiple times in the hardware, it is important to keep it as compact as possible. Placing the mRNA’s ROM in the mRNA module with a common arbiter instead of keeping a copy for each ribosome results in each ribosome consuming 65 lookup tables (LUTs) and 31 flip flops (FF) on average. To further improve that, we removed the allocation states (dashed line in Figure 4C) from the ribosome’s state machine and added allocation logic to the mRNA state machine. That is possible as only one ribosome can be at the allocation phase (at the 5′ end) at a given time. By doing so, we were able to reduce the size of the hardware ribosome to 48 LUTs and 22 FFs on average, improving the LUTs and FFs consumption by 26 and 30%, respectively (also see Figure S15).

### Parallel Hardware Results

Running at an 200 Mhz clock, the parallel hardware model implemented in the FPGA can consist of up to 4096 hardware ribosomes statically distributed among 512 mRNA molecules.

For each mRNA molecule, the maximal number of simultaneously active ribosomes is bounded by , where *m* is the mRNA length and *D* is the minimal distance between consecutive mRNAs. This is of course a bound that is very rare in physiological conditions. In this extreme case, 4096 ribosomes are needed to translate 128 mRNAs in *E. coli*. See the Methods section for a better utilization approach of the hardware ribosomes for cells with low concentrations of ribosomes.

As the model is not iterative, the runtime does not depend on the number of ribosomes or mRNAs and is given by  where *T*_clk_(FPGA) is the period of the design clock, *T*_sim_ is the real cell simulation time (20 min, for example), andΔ*T* is the time interval of a single step of the hardware model.

For *E. coli*, as mentioned before, Δ*T* = 1 ms. Therefore, since the parallel hardware model runs at 200 MHz, it takes  to model 20 min of a real cell. From Figure 2A, we see that the software model runs for 28.1 s for 128 mRNAs and 4096 ribosomes and 1.89 s for 128 mRNAs and 512 ribosomes. Therefore, for those cases, the parallel model runs 4683 and 315 times faster, respectively.

We conclude that this model is highly effective for intermediate and high amounts of ribosomes in term of execution runtime.

### Iterative Hardware mRNA

**Figure 5:**
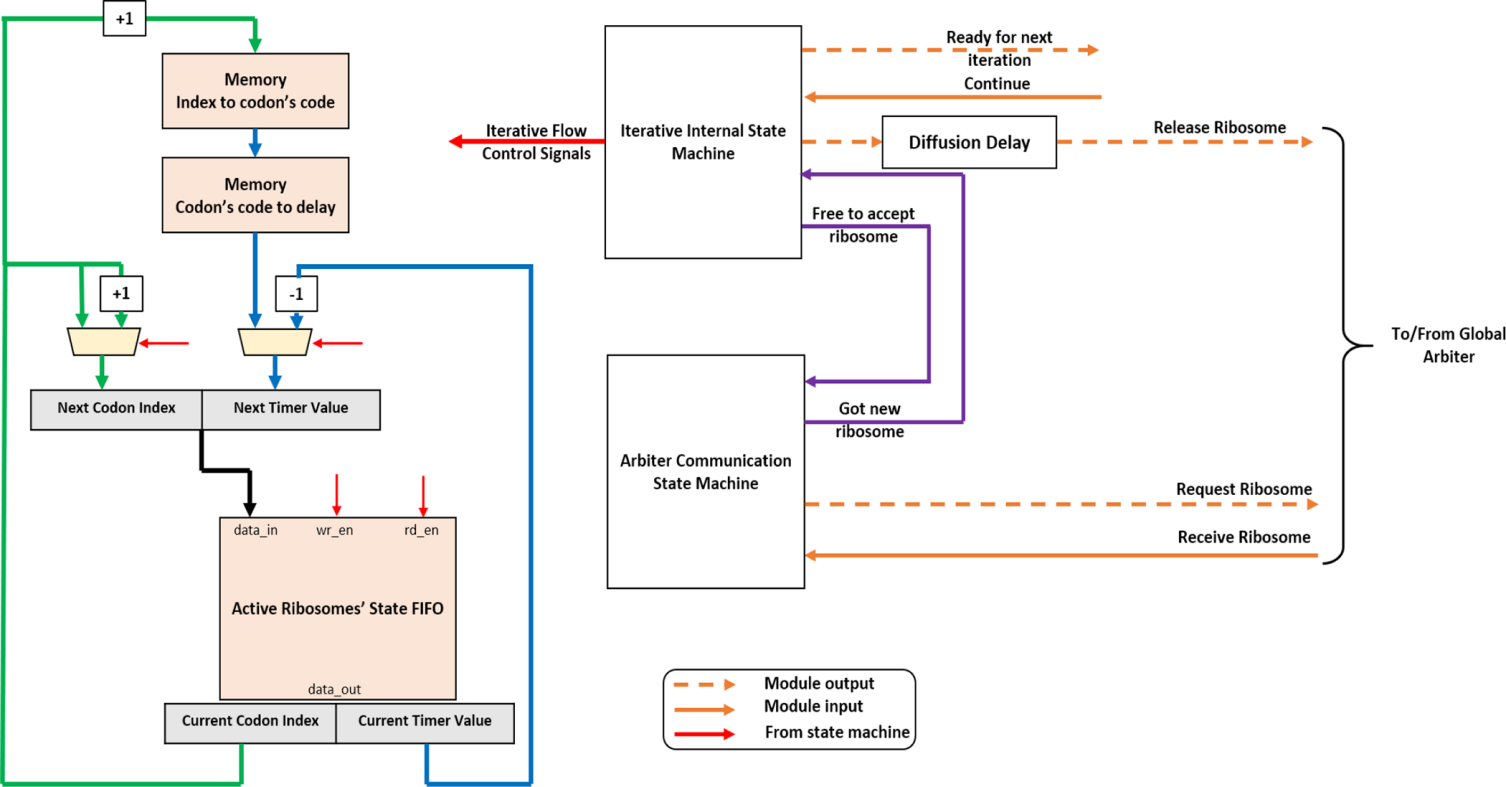
Iterative mRNA module block diagram. Here, we have a FIFO that contains the state of all active ribosomes. The state of the ribosome is read and updated from the FIFO by the state machine. When a specific ribosome advances to the next codon, the next translation delay is brought from the concatenated memories shown above. This module also contains a separate state machine for communication with the global ribosomes’ pool arbiter.

To improve the number of supported mRNAs and ribosomes, we next examined the iterative approach. The iterative mRNA module block diagram is shown in Figure 5 (see the Supporting Information for more details).

The codons’ data is stored in two concatenated memories—one containing the codon’s code and one mapping the code to translation delays (see the Methods section for more details).

Instead of having multiple replicas of hardware ribosomes (as in the parallel case), we only keep the state of each active ribosome (the current codon index and remaining translation time) inside a cyclic first-in first-out (FIFO).

To manage the system, we introduce two separate state machines. The first is to internally iterate over all active ribosome and advance their state. To synchronize the timing of all mRNA molecules, the state machine outputs a “ready” signal upon iteration completion and waits for all other mRNAs before moving to the next iteration (see the dash-circled area in Figure 3B).

The synchronization here affects the performance greatly as the “busiest” mRNA (the one with most active ribosomes) will hold back the update of all the other mRNAs. By doing so, the “busiest” mRNA dictates the time it takes the model to finish each step.

The second state machine is responsible for the communication with the global arbiter. This separation is done to have the global arbiter run as freely as possible at the designed clock speed. This feature is later shown to compensate for the lower hit probabilities of the uniform arbiter for the iterative case.

Finally, as before, the diffusion time is modeled by delaying the “release” signal.

### Iterative Model Results

By implementing this model, we were able to fit into the FPGA up to 1024 mRNA molecules at 200 MHz (with the required space for the maximal number of active ribosomes for each mRNA).

Since this model can accommodate more mRNAs, we decided to proceed and implement our proof-of-concept with the iterative model.

The performance analysis of this model is more complicated than the case of the parallel model since the runtime highly depends on the steady-state distribution of the ribosomes among the mRNA molecules. The empirical performance for various parameters is described later as part of the POC analysis.

### System Design Proof-of-Concept

To further demonstrate the effectiveness of hardware modeling, we decided to run the FGM and BGM algorithms presented in ref (22) using the iterative hardware model as described below.

In ref (22), optimization algorithms for improving the allocation of ribosomes in the cells by decreasing their traffic jams during translation were introduced. The algorithms introduced silent mutations within the coding regions. These do not affect the linear chain of the encoded protein amino acids but can affect the cell growth rate. Specifically, the algorithm introduced in ref (22) are:

(1) Forward gene minimization (FGM): Incorporates all silent mutations (from the beginning of the ORF) that improve the free ribosomal pool while not reducing/increasing the mRNA’s translation rate beyond some threshold. In each iteration, the mRNA that increases the free ribosomal pool the most is selected.
(2) Backward gene minimization (BGM): Similar to FGM but starting at the end of the modified region in the ORF and traversing backwards.

To reduce communication overhead as much as possible, we used the ARM cores in the Zynq processor to operate the FPGA and run the optimization algorithms.

**Figure 6:**
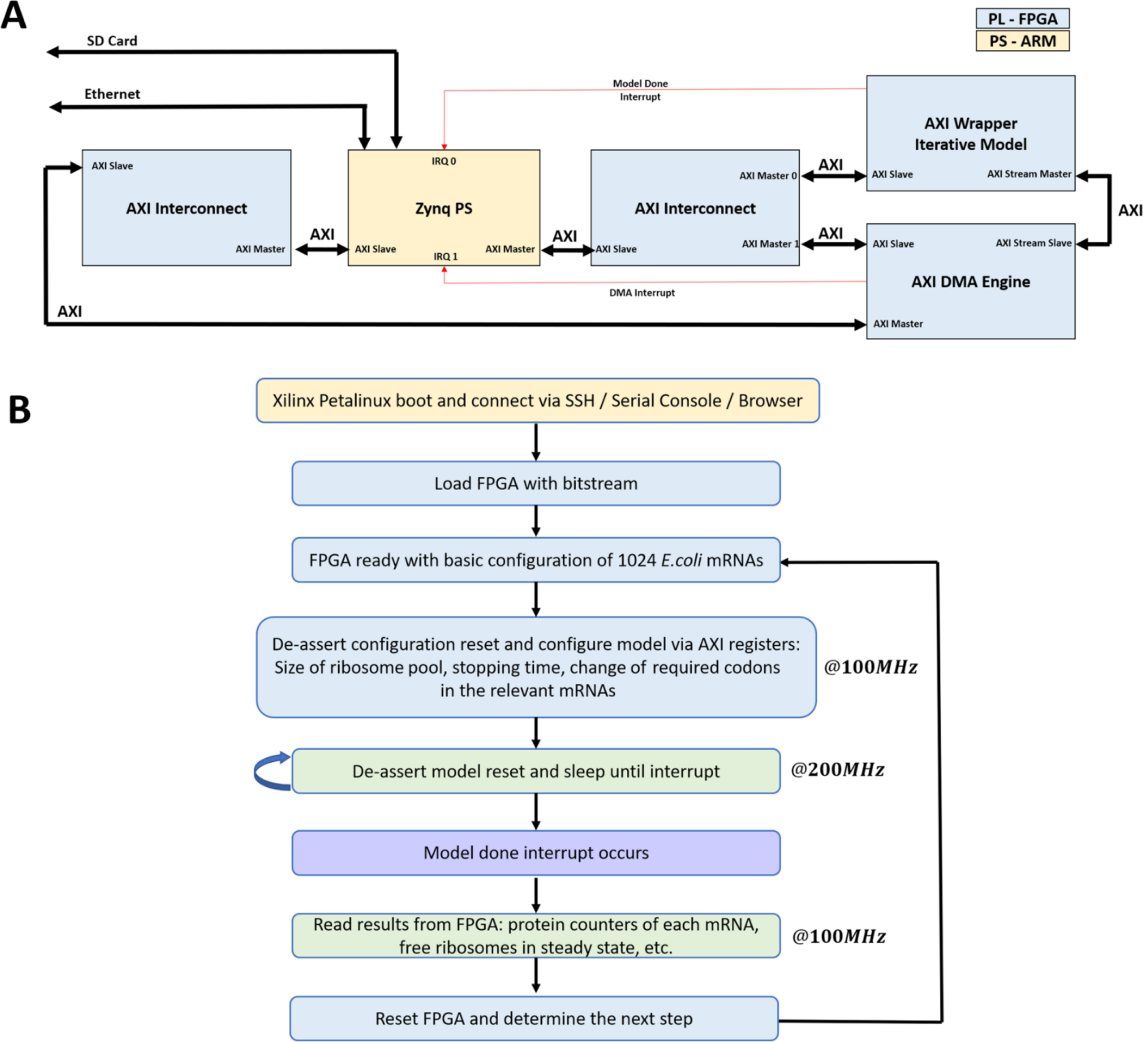
System POC design and flow. (A) Block diagram of the system including the FPGA part (in light blue) and the ARM part (light orange). (B) Flow graph to illustrate the order in which operations are carried throughout the POC.

The system design is shown in Figure 6A. Also, the general flow of running the POC is shown in Figure 6B (also see the Supporting Information). As shown, to connect the FPGA model to the CPU cores, we used the on-chip dedicated interfaces (named AXI) between the cores and the FPGA. This interface eventually generates a memory-mapped register read–write interface to the hardware. Also, for reading large amounts of data from the FPGA (for example, read all protein counters from all mRNAs), a direct memory access (DMA) engine can be connected to allow the FPGA direct access to the on-board CPU memory. Finally, when the model reaches the configured stop time, it raises an interrupt to the dedicated interrupt pins of the ARM cores.

### Verification and Validation of the Hardware Models

The hardware verification was performed by comparing the software simulator to the hardware outputs. When using the same random seed, we observed that the results were identical.

In order to test the accuracy of the hardware model predictions, we decided to model *Saccharomyces cerevisiae* and *E. coli* with the iterative model and the typical ribosome concentrations found in those cells.^42,43^

**Figure 7:**
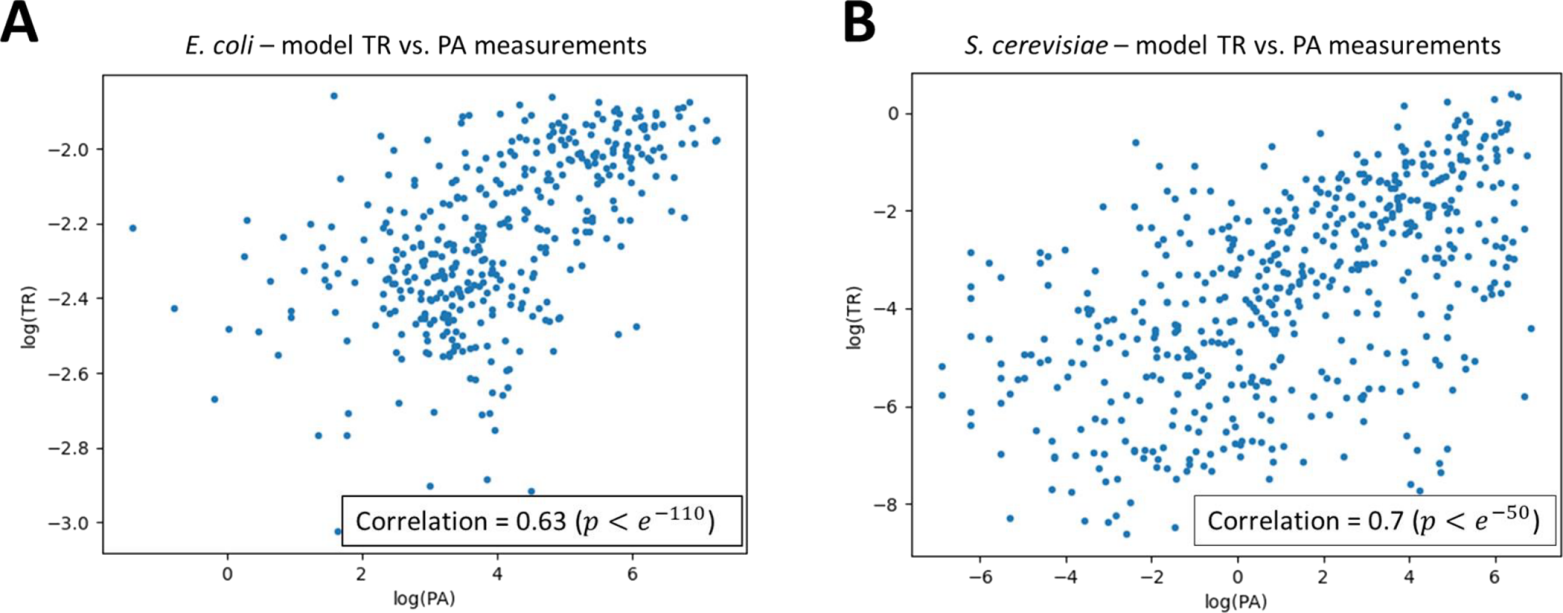
Comparison between model translation rate predictions (TR) and measurements of protein abundance (PA) in a logarithmic scale for (A) *E. coli* and (B) *S. cerevisiae*.

We then calculated the Spearman’s correlation between the predicted translation rate of proteins and real experimental measurements of protein abundance (PA). We got that the correlation is 0.63 (*P* < *e*^–110^) in *E. coli* (see Figure 7A) and 0.7 (*P* < *e*^–50^) in *S. cerevisiae* (see Figure 7B and additional details in the Methods section). Those correlation results are considered very high in the field (and similar to typical correlation between to experimental measurements) since cellular measurements are typically noisy, biased, and related to PA while we predict the translation rate (there are no large-scale measurements of translation rate).

### Proof-of-Concept Results: Ribosomal Traffic Jam Optimization Based on Dedicated Hardware

**Figure 8:**
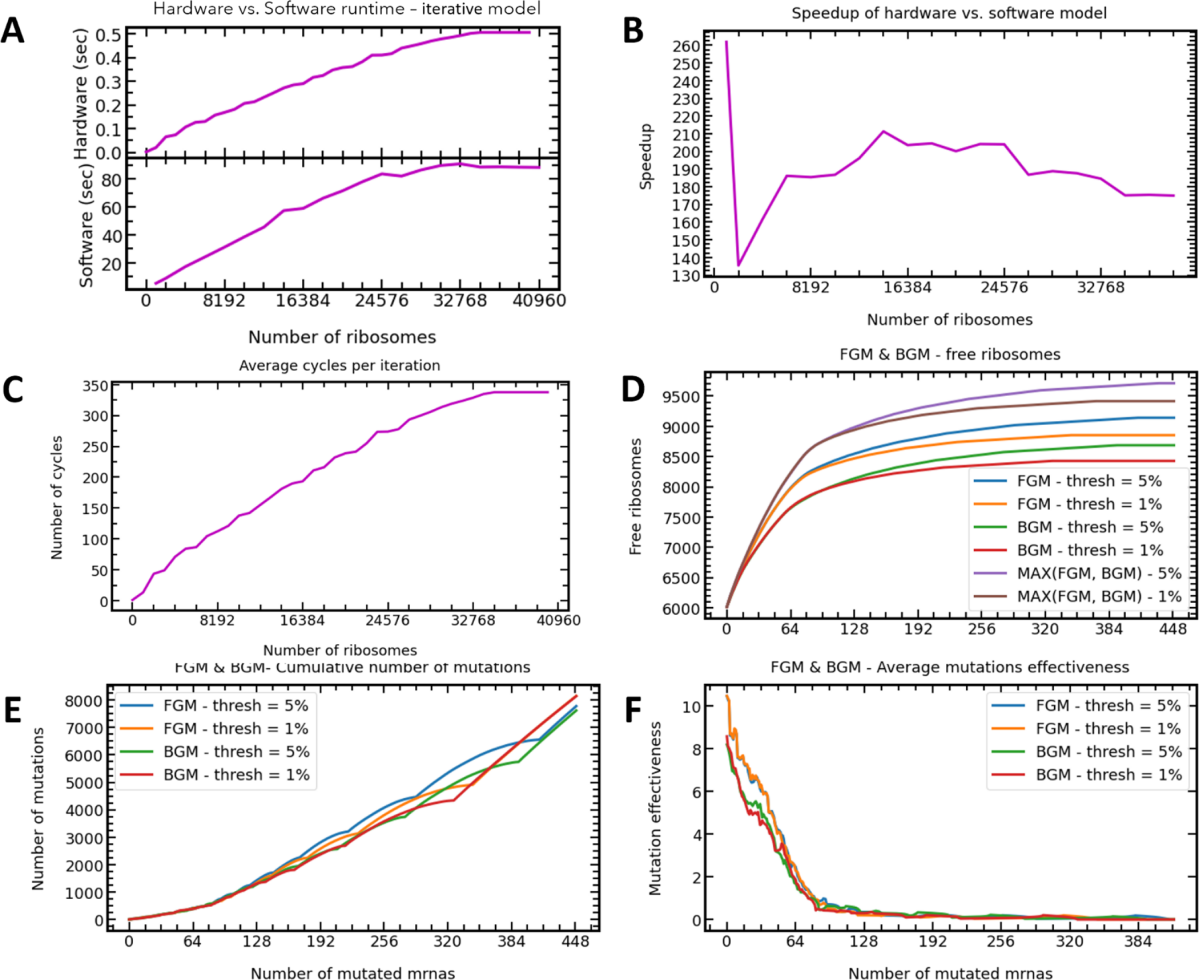
Results from full-system POC. (A) The average runtime of a single model run in software vs hardware (iterative model). Used parameters: 5 min of real cell time and 1024 mRNAs. (B) Speedup of hardware iterative model vs software model with the same parameters as in (A). We observed that the hardware iterative runs up to 262 times faster than the software model. (C) Average number of clock cycles per iteration of the iterative model in hardware versus total number of cell ribosomes. (D–F) Results of FGM and BGM on hardware with 1024 mRNAs and enough ribosomes to saturate the cell (around 38,000) as done in the referenced paper. The threshold is the allowed variation for each mRNA in the proteins’ production rate. Max (FGM, BGM): For each mRNA, the method that yields the best result was chosen and used. In (F), the mutation effectiveness is the ratio between the number of newly added free ribosomes and the required number of mutations of the specific mRNA. The graph is sorted similarly to (D) and (E).

Figure 8A,B presents the speedup of the hardware iterative model versus the software model. Here, the speedup is defined as the ratio between the software and hardware latencies. As expected, we can see that the runtime depends on the number of ribosomes. As the execution time of the mRNA iterative module increases with the number of ribosomes, it increases the iteration time (see Figure 8C for average iteration time of the iterative model as a function of the number of ribosomes). Also, as illustrated, the runtime flattens at some point for both the hardware and the equivalent software model. That is the saturation point in which the mRNAs have as much ribosomes as they can consume; this, however, is not a usual physiological condition. Recall that we have already seen the same saturation point in Figure 4A for 32,768 ribosomes. Figure 8C also proves that the uniform arbiter stays effective even for low hit probabilities. As the cell is more saturated with ribosomes, the hit probability reduces but the latency of each iteration increases. That is the reason why we kept the communication state machine in Figure 5 independent of the iterative state machine—to allow receiving and requesting ribosomes at the high 200 MHz clock speed regardless of the iterations’ state.

Figure 8B suggest that the speedup of the hardware iterative model varies between 180 and 262.

Figure 8D depicts the results for the optimization POC using the FGM and BGM algorithms. In the POC, we started with about 6000 free ribosomes (saturated cell as done in ref (22)). As depicted, by inducing silent mutations from codons 11 to 50, we can significantly increase the effectiveness of the ribosomes without impacting the translation rate of each mRNA (up to a given threshold). We can also notice that on average, the FGM algorithm yields better results than the BGM algorithm, similarly to ref (22).

In Figure 8D, we added graphs for a combined solution—for each mRNA, we are choosing the configuration that yields the best results. We can see that although the BGM is less effective on average, in some mRNA molecules, it performs better than the FGM algorithm.

Furthermore, from Figure 7E,F, we can see that the mRNAs that yield the best results also require less mutations. That is important because now, we have a small subset of roughly 64 mRNAs that is interesting to explore for further optimizations and can fit in the considerably faster parallel hardware model.

To produce these results, for each algorithm (FGM/BGM) and each threshold (1 or 5%), we needed an average of 110,000 model runs. For 1024 mRNAs, 38,000 ribosomes (for saturation), and 10 min of real cell time, the iterative model runs for approximately 1 s and the software model runs for 191 s. Therefore, a such a single optimization procedure took 30 h with our hardware iterative model and is approximated to take more than 8 months with the software model.

## Discussion

In this study, we describe for the first time a novel approach that can be very useful in synthetic biology: whole-cell modeling and engineering of translation based on a dedicated hardware. The model (whole-cell model of translation) we analyze here was chosen as a POC example, and our approach can of course be implemented to solve various similar problems and models.

The complexity of synthetic biology models dramatically increases in the recent years^7,9,11,19,21,24−27^ in a rate higher than the increase in computation cost. In some cases, when the models and algorithms are too extensive, the simulations may become the bottleneck of the development process.

Thus, we suggest that in some cases, the usage of a software in the Design–Build–Test–Learn (DBTL) synthetic biology cycle will be replaced by dedicated hardware (see Figure 1B).

### Comparison between the Hardware Models

By examining the results, it is easy to see that the iterative model runs slower (up to 260 times faster than software) but can accommodate more mRNAs (up to 1024 mRNAs) and ribosomes as the parallel model runs much faster (up to 4690 times faster than software) but contains less molecules (up to 128 mRNAs and 4096 ribosomes).

These results could be anticipated since this is a common tradeoff in designing hardware—runtime vs chip area. In the parallel model, the ribosomes can run in parallel to each other for the price of having them implemented in hardware. Conversely, in the iterative model, the ribosomes are being run sequentially (and therefore, more slowly) by the mRNA state machine and can only therefore consume the area needed for their state.

As the iterative model can accommodate more mRNA molecules, it is best suited for whole-cell modeling and optimization of translation. In order to explore a smaller part of the cell, the parallel model can be used to cover much more configurations in the same amount of time.

Going back to the above POC, we can first run the FGM and BGM algorithms on large amounts of mRNAs and then we can, for example, use the simulated annealing algorithm to further optimize the 64 “best” mRNA molecules using the parallel model.

### Our Approach Can Be Used for Modeling Other Type of Intracellular Competitions and for Changing Intracellular Conditions

In this work, we chose to demonstrate our approach by modeling competition over a limited ribosomal pool as this is currently the most studied intracellular model in the field, mainly due to the fact that most of its parameters can accurately be estimated from experimental data. It is important to emphasize the fact that due to the competition on limited cellular resources such as ribosomes and tRNAs, even a small intracellular circuit (e.g., 1–3 genes) can affect the entire cell and should be engineered based on a whole-cell model.^8−11^ This is specifically true when the expression levels of the circuit need to be high and induce huge load on the host. We would like to emphasize the fact that translation consumes more than 75% of the energy in the cell;^44−53^ thus, it is not surprising that translation is an important aspect in such cases.

In the future, similar approaches can be used for modeling other intracellular aspects such as competition of tRNA, miRNA, transcription factors, and more^8^ alongside more details related to the biophysical process (e.g., operon structure and reinitiation^54^).

For example, for modeling competition of tRNAs, we suggest examining a similar approach to the arbitration over the finite ribosomal pool. We can consider 61 pools of tRNA molecules (excluding the stop codons) that receive requests from all ribosomes. It seems that the challenge here might be the routing of all requests from all ribosomes to this pool in a ribosomal saturated cell.

It is important to emphasize that during the intracellular engineering process, the parameters of the models may change. For example, the concentrations of the tRNA molecules mainly impact the codons’ translation delay, and the values used here are the average based on measurements from real cells and therefore already include the influences of various tRNA concentrations. Thus, the demand for tRNA molecules in the cell might change when inducing silent mutations to several mRNAs as suggested in this paper. If the change in the demand is substantial, it might impact the translation delay of several codons. By going back to the first stage shown in Figure 1B with new experimental data, those variations can be corrected.

Finally, we want to emphasize the fact that according to our experience, whole-cell models based on differential equation are also very slow; thus, although the resolution of the model usually decreases, this is not a solution to the challenge of performing very fast simulations. This suggests that the hardware solution may also be relevant for accelerating whole-cell models based on differential equations.

### From 1024 mRNAs to a Whole-Cell Translation Model

In most cases, real cells contain thousands of mRNA molecules and even more ribosomes. It is often needed to model a whole translation cell and not just a subset of chosen mRNAs. For that purpose, the following changes should be examined.

First, one can simply consider using a bigger FPGA. The FPGA used for this POC is Xilinx Zynq Ultrascale+ ZU7EV, which contains 230 K LUTs and 11 Mbit total BRAM memory. By using Xilinx Virtex Ultrascale+ VU57P, which contains 1.3 M LUTs and 70.9 Mbit total BRAM memory, it is expected to be possible to fit much more mRNAs and ribosomes into the design. As a matter of fact, ignoring scaling considerations (as the enlargement of the global arbiter for more mRNAs), VU57P is expected to support up to 2900 mRNAs in the parallel model and much more mRNAs in the iterative model (i.e., all the mRNAs in the case of most bacterial species).

Moreover, as the main limitation of the parallel model is the amount of hardware ribosomes, a considerable challenge when designing it was efficiently distributing the hardware ribosomes between the mRNA modules (see the Methods section for more details). Perhaps it is possible to come up with a more dynamic approach so that the hardware ribosomes could be shared by several mRNAs. This problem of dynamically allocating a common resource resembles the way virtual memory is implemented in the hardware.^55,56^ A similar approach can perhaps be implemented here. If so, it is interesting to examine how it affects the performance as the main advantage of the parallel model over the iterative one is its performance.

Also, we can consider a solution in which multiple FPGAs are connected to form a large system. Platforms that support several FPGAs already exist in the market today. By using such platforms, we can distribute more mRNAs between the FPGA chips and split the global ribosomes’ pool to several small pools that are communicating with each other. In addition, each FPGA can simulate one aspect or stage of gene expression (e.g., one FPGA for transcription, one for transport, one for translation, etc.).

Finally, one can consider implementing an application-specific integrated circuit (ASIC). FPGAs are quite comfortable for prototyping as done here but are quite inefficient in the matters of power consumption, area utilization, and operating frequency in comparison to ASICs.^57−59^ Specifically, for example, according to ref (59), we can expect that for a full system design, the equivalent ASIC area will be up to 10 times smaller than the FPGA area. Therefore, we can expect that by having an ASIC of the same die size as the FPGA, we can potentially support up to 10,240 iterative mRNAs and 1280 parallel mRNAs. Using the architectures presented in this paper, it is possible to implement a high-speed, highly configurable ASIC that can accommodate large numbers of mRNAs and ribosomes.

Also, the lightweight randomization mechanism presented here can be easily adapted to randomize the translation delay of the codons. By doing so, the model can become completely stochastic at the cost of consuming more FPGA resources.

Another feature that can be considered is modeling the degradation of ribosomes and mRNA molecules in the cell. The current architecture of the hardware supports modifying the ribosomes’ levels to simulate degradation as the number of ribosomes is a software-controlled parameter of the hardware that can be dynamically changed throughout the simulation. Regarding the mRNA molecules’ degradation, to support the degradation feature, the enabled signals (that already exist in hardware) for the hardware-mRNA modules should be routed to the software interface. This is a simple hardware change that can allow the software algorithm to impact the mRNAs’ degradation by randomly disabling mRNA molecules according to the desired heuristic. However, one challenge related to this aspect is related to the lack in experimental measurements of the half-lives of mRNAs and ribosomes.

## Methods

### Source of the Data and the Parameters of the Models

The parameters of the model (initiation rates, elongation rates, mRNAs codons’ list with various lengths, and total number of ribosomes) were based on ref (9) and are inferred from ribo-seq experiments. The parameters there were inferred by fitting the biophysical model to the ribo-seq data of all mRNAs of *E. coli*. The number of mRNAs in *S. cerevisiae* is from ref (60) and that in *E. coli* is from ref (43), the number of ribosomes in *S. cerevisiae* is from ref (42) and that for *E. coli* is from ref (61), and *D* (ribosome size) for *E. coli* is from ref (62) and that for *S. cerevisiae* is from ref (63). Our model does not directly consider operon structures in the case of *E. coli*. This structure specifically affects the initiation rate to coding regions inside the transcript as it is a combination of “direct” initiation and reinitiation (after translation termination or the previous coding region^54^).

While current time < simulation time:

(1) For each mRNA, get the last ribosome’s release time
(2) Update the free ribosomes’ counter
(3) If no ribosomes are free at the current time:
  a. Advance current time to min (mrna_i_.release time)
  b. Continue
(4) For each mRNA, get the ribosome’s request time
(5) If none of the mRNAs are requesting at current time:
  a. Advance current time to (mrna_i_.request time)
  b. Continue
(6) Randomly grant ribosomes to the requesting mRNAs with respect to the amount of currently available ribosomes
  a. For each receiving mRNA object, calculate the new time vector

However, we do model the right initiation rate to each coding region since it was inferred by fitting the biophysical model to the ribo-seq data of all the mRNAs of *E. coli*, which reflect both components (direct initiation and reinitiation).

In the case of comparison of the translation rates from our models and PA, since we are limited to 1024 mRNA molecules in the current FPGA, in each organism, we chose the genes that are with the highest levels in the cell and replicated each gene type with proportion to the cellular mRNA level of that specific gene.

### Software Model Pseudocode

Following is a pseudocode for the time-event vector calculation of each mRNA molecule upon receiving a new ribosome:

*t*_list[0] = current time + ribosome initialization delay
  for *i* in [1..*L*–*D*):
  (1) blocking time of previous ribosome =
    max(0, prev_*t*_list[*i*+*D*] – *t*_list[*i*–1])
  (2) translation time = mRNA codon delay [*i*]
  (3) *t*_list[*i*] = t_list[*i*–1] + (1) + (2)

for *I* in [*L*–*D*:*L*):

(1) *t*_list[*i*] = *t*_list[*i*–1] + mRNA codon delay [*i*]

*t*_list[*M*+*D*] = *t*_list[*L*+*D*–1] + diffusion time

Here, *L* is the length (in codons) of the mRNA molecule, *D* is the minimal distance between consecutive ribosomes and *t*_list[*i*] is the absolute time in milliseconds for finishing the *i*th step of the current ribosome.

The global scheduler keeps track of the generated time vectors of all mRNAs to determine the exact times in which ribosomes are allocated to specific mRNAs, ribosomes are freed, and proteins are generated. Following is a pseudocode of the global software scheduler.

For this pseudocode, we get the following execution time distribution shown in Table 1.

**Table 1:** Execution Time Distribution in the Case of the Global Scheduler

section	(1)	(2)	(3)	(4)	(5)	(6)	(6a)
execution time (%)	19.1%	0.5%	9.6%	7.5%	0.5%	0.2%	60.7%

### The Global Ribosomes’ Pool Arbiter Local Release Counter

As previously mentioned, the round-robin arbiter iterates over all mRNAs, and therefore, it takes exactly *m* clock cycles to return to the same mRNA molecule. During that time, ribosome release events may occur. To take that into consideration, we added a release counter for each mRNA release signal. The size of this counter can be determined as follows: Given the minimal codon’s delay as *d*_minimal_ and *D* as before, it follows that the maximal number of release events during the arbiter’s iteration is given by
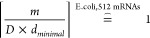


For the uniform arbiter, the number of release events that can occur until the arbiter reaches the same mRNA is different.

First, let us calculate the number of clock cycles that the arbiter takes to reach the same mRNA molecule. Here, this number is not a constant (as in the round-robin case) but a random variable. As shown, the arbiter randomizes an index from 0 to *m* with probability close to , as it is designed to be uniform.

Thus, we have a list of independent identical distributed (iid) Bernoulli experiments with a probability of , and we ask what number of experiments is required to receive two success events with high probability. Having *E* experiments , we get
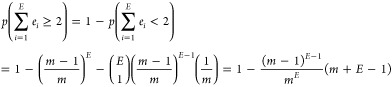


**Figure 9:**
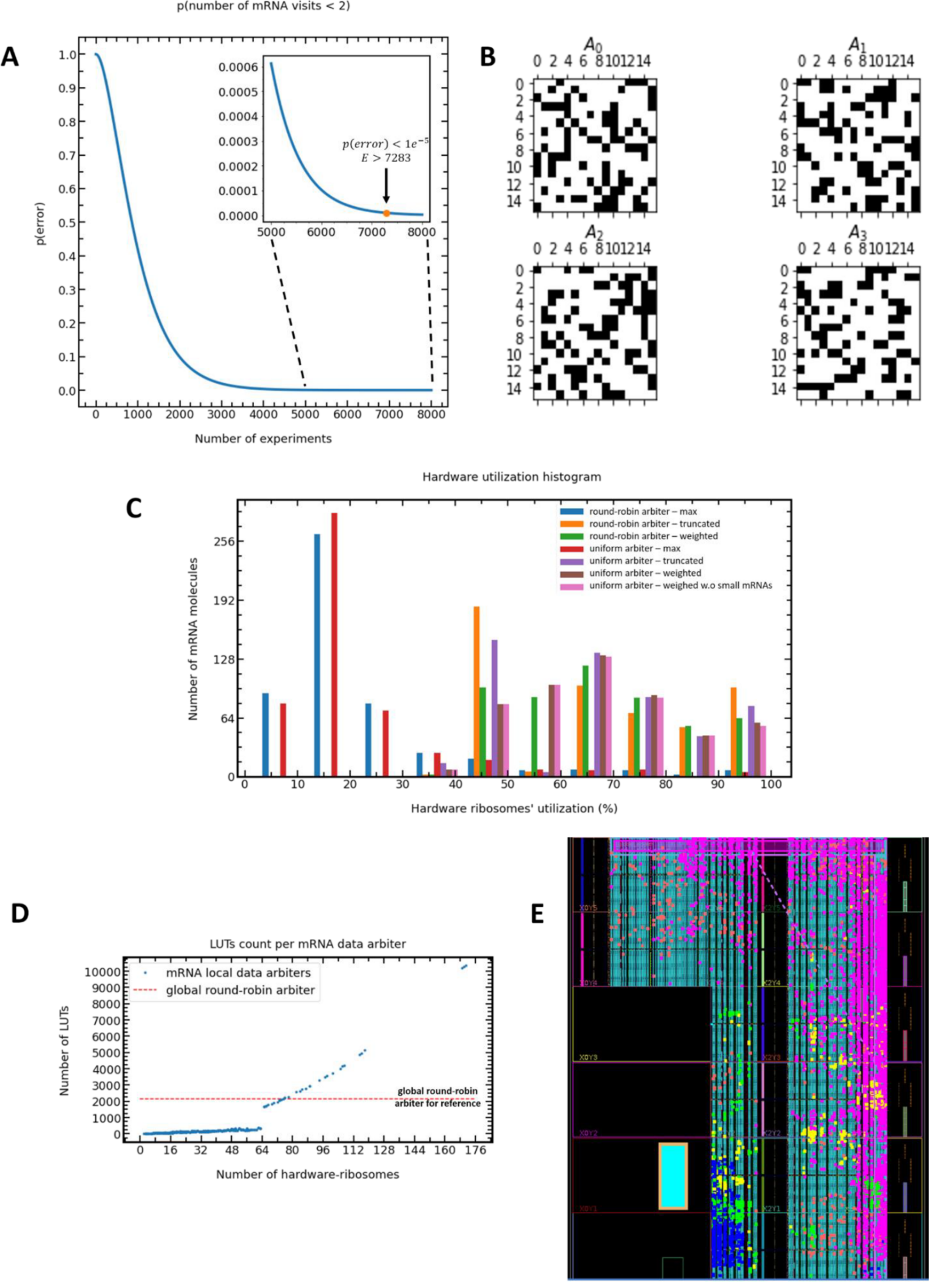
Methods section graphs. (A) Number of consecutive Bernoulli (with *P* = 1/512) experiments needed for at least two success events with miss probability lower than 1 × 10^–5^. (B) 16 bit matrices for generating random numbers in hardware. (C) Utilization of the allocated hardware ribosomes using different arbiters and different allocation methods: max, each mRNA has  hardware ribosomes; truncated, each mRNA has  ribosomes; weighted, the total amount of hardware ribosomes is distributed by a weight function. (D) Local mRNA data arbiter size in LUTs as a function of the number of hardware ribosomes; number of mRNAs that are free to receive new ribosomes as a function of time. (E) FPGA floor planning—in pink rectangles—guiding the placer to place the arbiter around the right and top edges.

For 512 mRNAs, we get (see Figure 9A)
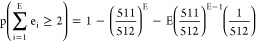


Consequently, the maximal number of release events during the arbiter’s iteration is now with overflow probability *p*_error_
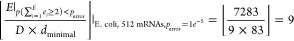
which requires a 4 bit counter for each mRNA molecule.

### Uniform Random Number Generators (URNG)

There exist plenty of hardware URNG implementations,^64−69^ some of which are optimized specifically for FPGAs.^64−66^ Since we wish to keep the URNG logic as compact as possible, the URNG suggested in ref (65) is the most relevant implementation for our need. There are two types of URNGs: true-URNGs and pseudorandom URNGs (PRNGs). True-URNGs are more relevant for cryptographic usages where high quality of unpredictable random sequences should often be generated. True-URNGs are often based on physical randomness that is generated through various methods^64^ and are rather complex. However, for our usage, PRNGs are sufficient.

By following the method suggested in ref (65), we had to come up with two matrices that operate on a state register to generate the random number
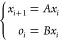
Where *x_i_* is the internal state register (with dimension *n*), *o_i_* is the output of the PRNG in the *i*th clock cycle (with dimension *m*), and  and  are generation matrices. By choosing the right  matrix, the sequence {*x_i_*} can have a cycle of 2^*n*^ – 1. In Ultrascale+ chips, each LUT has six entries. Therefore, for having an LUT-efficient matrix multiplication of *Ax_i_*, we should keep the number of ones in  rows below 6. We also wish to make sure that all bits in the state register take place in the calculation of the next state. The authors in ref (65) considered software-efficient algorithms for generating adequate matrices as *n* grows. For our case, small matrices of up to *n* = 32 suffice, and for their generation, we used the following simplified algorithm.

For illustration, for *n* = 16, we get the matrices shown in Figure 9B. It is easy to see that each row or contains at most six ones.

while True:
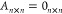

(1) for *i* from 0 to *n*:
  a. *k* = randomize number of LUT inputs from 4 to 6
  b. indices = randomly choose *k* indices from 0 to *k*–1
  c. *A*[indices] = 1
(2) *A*_sum = sum *A* rows
(3) If 0 in *A*_sum (make sure all state bits effect the next state):
  a. Continue
(4) Calculate *x*_*i* + 1_ = *Ax_i_* for *i* from 0 to 2^*n*^ – 1
(5) If |set({*x_i_*})| < 2^*n*^ – 1 (check if *A* generates a full cycle):
  a. Continue
(6) Return *A*

The same approach was used for . By using this approach, we were able to generate two *A*_16 × 16_ matrices (to operate on a 32-bit state register) and one *B*_9 × 32_ matrix that produces a random sequence with *P* = 0.992. For reference, we get *P* = 0.8 for random sequences of the same length generated by the “random” package in Python. The main reason for this improvement is that Python (for instance) randomizes large integers, while the approach here is tuned for small integers.

Apart from generating a high-quality uniform stream, this PRNG is quite efficient: {*A*_0_, *A*_1_} × *x_i_* requires 32 LUTs and *B*_9 × 32_*x_i_* requires only 9 LUTs. As the state register here is advanced separately as two concatenated state registers (one for each matrix) when the first state is advanced only after 2^*n*^ – 1 steps of the second state, an extra 16 bit counter is required.

### Parallel Model Hardware Ribosomes’ Buffer Size

The maximal theoretical number of active ribosomes operating on the same *i*th mRNA of length *L_i_* simultaneously is given by . Therefore, the average number of hardware ribosomes needed for *m* mRNA molecules is given by .

By implementing that design, we were able to fit into the FPGA 128 mRNAs at 200 MHz. Here, as opposed to the iterative model, the bottleneck is the LUT utilization and (not the BRAM utilization) as the ribosomes occupy most of the FPGA and are composed of the state machine shown in Figure 4C.

In real *E. coli* cells, there are approximately between 20,000 to 50,000 ribosomes and 4100 mRNA molecules.^9^ Keeping that ratio when modeling 512 mRNA molecules with 2048 ribosomes, we get the ribosome hardware utilization histogram shown in Figure 9C. The ribosome hardware utilization is the maximal number of simultaneously active ribosomes out of the available hardware ribosomes for the specific mRNA. That shows that the hardware ribosomes that consume the most FPGA resources are barely utilized. That is not surprising since the number of ribosomes in a cell is much lower than . Moreover, in Figure 9D, we can see that as the mRNA has more hardware ribosomes, its data arbiter consumes more LUTs. That is due to the wide multiplexer in the arbiter that chooses the codon index that is used for the mRNA ROM input (as shown in Figure 3C). In Figure 9D, we can also see the global round-robin arbiter LUT utilization for reference.

Keeping that in mind, we next investigated a different method to distribute the hardware ribosomes between the mRNA modules. By summing the maximal number of active ribosomes for all 512 mRNAs in a cell with 2048 ribosomes, the number of hardware ribosomes will be
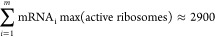


That means that if we could predict that number, only 2900 hardware ribosomes would be needed for an accurate 512 mRNAs operating with 2048 cell ribosomes. As mentioned, our hardware can accommodate 4096 hardware ribosomes. For 512 mRNAs and 2048 cell ribosomes, the maximal number of simultaneous active ribosomes is 2048. The question is how to distribute the 4096 available hardware ribosomes between the 512 mRNAs in such a way that each mRNA, even at its most occupied moment, does not miss any ribosomes granted by the arbiter.

We first tried the following approach: Assuming 2048 ribosomes are distributed uniformly among 512 mRNAs, the average amount of active ribosomes on each mRNA should be around 4; so if we double that in hardware-ribosomes per mRNA (as we could fit 4096 hardware ribosomes in a single chip), each mRNA, with high probability, will not saturate its hardware ribosomes.

As shown in Figure 9C, that leads to 96 mRNAs that saturate their hardware ribosomes for the round-robin arbiter (80 for the uniform arbiter). To further improve that, we seek to find a weight function that can predict the utilization of hardware ribosomes. The factors that should be taken into consideration are:

(1) mRNA length – As it is longer, it is more probable to have more active ribosomes.
(2) Codon translation time – As it takes longer for a ribosome to move along the mRNA, it is more probable to have more active ribosomes.
(3) Entry time – As the initiation delay plus the time it takes a ribosome to clear the mRNA 5′ end by moving D codons is longer, the mRNA is expected to request ribosomes at a lower rate.

Taking those parameters into account, we assigned each mRNA the following weight:
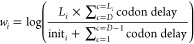
Where init_*i*_ is the initialization time of the *i*th mRNA. Here, we used the logarithm to smoothen the weights. By assigning those weights, with available *HR* hardware ribosomes (4096 in the case of Ultrascale+), the buffer size of the *i*th mRNA is given by
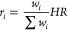


By applying this approach, we were able to reduce the number of mRNAs that saturate their hardware ribosomes to 57 for the uniform arbiter (see Figure 9C). See the Discussion section for ideas on improving that even further.

Finally, consider the case in which we wish to synthesize a single mRNA molecule and inject it into an existing cell (such as *E. coli*). In that case, we might wish to model intracellular interactions of thousands of different variants of that molecule while the rest of the cell remains the same. That use case is highly relevant, for instance, in the process of vaccination development. As the software simulation can run for months, enumerating over thousands of variants, the hardware model becomes quite attractive. In that case, a long simulation can first reveal the actual utilization of each mRNA molecule in the cell. Then, the utilization can be translated to the allocation number of hardware ribosomes for the cell mRNAs. By doing so, we will be able to fit even more mRNA molecules without hardware saturation in a single FPGA since as shown above, for 512 mRNAs and 2048 ribosomes in the cell, we only need 2900 hardware ribosomes (and we can fit 4096).

### Improving Memory Usage of the Iterative Model

To store the codon’s delay list for each mRNA molecule, we first used a map between the codon’s index to its delay for simplicity. The utilization report revealed that on average, each mRNA module uses one BRAM for the codons’ delay ROM. We found that we can reduce its size by replacing it by two concatenated ROM memories as follows.

The first ROM maps each codon index to the codon’s code. Each codon consists of three nucleotides, and each nucleotide can contain one of four possibilities. For coding the nucleotides, only 2 bits are required (four possibilities). Therefore, each codon can be coded using 6 bits.

The second concatenated ROM maps a codon’s code to the codon’s average translation delay (for the deterministic model the average suffices).

By using the original, single-ROM method, we get
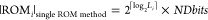
Where *NDbits* denotes the maximal width in bits of the codons’ delay (15 bits for *E. coli*) and *L_i_* denotes the length of the *i*th mRNA molecule. We use 2^⌈log_2_*L_i_*⌉^ instead of *L_i_* since in the case of BRAMs, one cannot use a fraction of BRAM that is not a power of 2.

By using the double-ROM method, we get
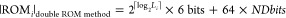


Also, the codon code to codon delay table can be implemented as *NDbits* 6-input LUTs (15 LUTs in *E. coli*), as LUT is basically a ROM with a 6 bit address and one output bit. That is important as the bottleneck here is the BRAMs and not the LUTs.

Therefore, the average improvement by implementing the double-ROM method is given by
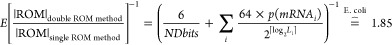


### System Complexities

It is important to keep the system operation simplified as possible for non-engineer users. For that, we compiled a Linux operating system for the Zynq ARM processor and wrote a device driver to communicate with the memory-mapped registers.

As Python is a more approachable coding language than Verilog (the language in which the hardware is developed), we decided to run a Jupyter Notebook server using Xilinx PYNQ. The server can be connected via a browser. The optimization algorithms shown in this paper where coded in Python and run on this Jupyter server on the CPU cores of the Zynq chip.

Apart from optimizing memory and LUT utilization as shown above, we had to take further steps in order to squeeze the design into a single FPGA at 200 MHz.

First, to ease the router’s task, we had to use floor planning to direct the placer to the general on-chip locations to which it should place different logic blocks (see Figures S28 and S32). In Figure 9E, we can see, for example, that we directed the placer to place the uniform arbiter on the upper right corner of the chip. That is because it should be connected to all mRNAs but not to the ARM processor that appears on the bottom left corner.

Next, the bigger number of mRNA molecules grows, the wider the multiplexers of the arbiter get. For 1024 mRNAs, we had to pipeline the multiplexers (at the cost of extra clock cycles) to meet the timing constraints (see Figures S21 and S31).

Also, to grant the ribosomes to the mRNAs in large amounts, we avoided the demultiplexer altogether and replaced it with an index of the current served mRNA that is routed to all mRNA molecules.

Moreover, to further improve timing, we used two different clocks as shown in Figure 6B. For configuration and result readback, we used the slow 100 MHz clock as communication is not the bottleneck. For the model itself, we kept the 200 MHz clock to avoid performance degradation.

Finally, we have many buses and signals that should reach all mRNA molecules (mRNA configuration signals from the ARM processor and the served mRNA index from the global arbiter, for example). To help the router’s convergence, we had to duplicate some of those high fanout nets and their logic at the cost of area (see Figure S30).

## Supporting Information Available

Additional information about the presented hardware architectures, system complexities encountered in the proof-of-concept, details about the RTL hardware modules, detailed analysis of a previous work done in Tuller’s lab, some conclusions that led to the architectures presented in this paper (PDF)

## Notes

The code developed and used in this study can be downloaded from https://www.cs.tau.ac.il/~tamirtul/mRNA-HW.
