# Supplementary material for: Accelerating Whole-Cell Simulations of mRNA Translation Using a Dedicated Hardware
Source: ACS Synth Biol. 2021 Nov 23;10(12):3489–506. doi: 10.1021/acssynbio.1c00415 (PMC8689694; doi:10.1021/acssynbio.1c00415)
Supplement: Supplementary file 1 — sb1c00415_si_001.pdf [file sb1c00415_si_001.pdf]

Supplementary Materials

Accelerating whole cell simulations of mRNA translation using a dedicated hardware

David Shallom[1], Danny Naiger[2], Shlomo Weiss[1], Tamir Tuller[2,*]

[1] School of Electrical Engineering, Tel-Aviv University, Tel-Aviv,

69978, Israel.

[2]Department of Biomedical Engineering, Tel-Aviv University, Tel-Aviv, 69978, Israel.

1 TABLE OF CONTENTS

2 Hardware Iterative model – initial Design 1
2.1 Initial mRNA module – memory utilization analysis 2
2.1.1 Memory blocks 2
2.1.2 Utilization Analysis 4
2.2 Initial mRNA module – memory optimizations 5
2.2.1 mRNA codons' ROM size 5
2.2.2 Ribosomes' state size 7
2.3 Initial mRNA model - Inaccuracies 9
2.3.1 mRNA state machine bias 9
2.3.2 Ribosomes' allocation bias 11
2.4 Initial design – conclusions 13
3 Parallel model – additional information 14
3.1 First Results 16
3.2 Utilization 19
4 System proof-of-concept details 21
4.1 Zynq PS 21
4.2 Using Xilinx PYNQ 23
4.3 Registers interface 23
4.4 PL_CONTROL package 24
5 HDL details 26
5.1 Model top hierarchy 26
5.2 Model top interface and further details 26
5.3 Round-robin global arbiter 29
5.4 Uniform global arbiter – implementation details 30
5.4.1 Pipelined multiplexer 30
5.4.2 PRNG 31
5.5 Uniform arbiter – interface and parameters 35
5.6 Parallel mRNA module – internal modules 36
5.6.1 Hardware ribosome module 36
5.6.2 Large delay module 38
5.6.3 mRNA data arbtier 40

5.7 Parallel mRNA module – parameters and interface 40
5.8 Iterative mRNA module 41
5.8.1 Iterative mRNA data module 41
5.9 Iterative mRNA module – interface and parameters 42
5.10 AXI wrapper modules 44
5.11 Design adjustments for better implementation convergence 46
6 E.coli data 50
6.1 Codon translation delay 50
7 Appendix A – Xilinx Device Resources 51

2 Hardware Iterative model – initial Design

As mentioned in the main text, our initial design did not contain a global arbiter for the assignment of ribosomes to mRNA molecules. Instead, our first hardware architecture was greatly inspired by the software implementation. Each mRNA molecule was implemented by a parametric RTL module with its own: state machine, ribosomal state management, initiation time, diffusion time and codon's delays list, as follows:

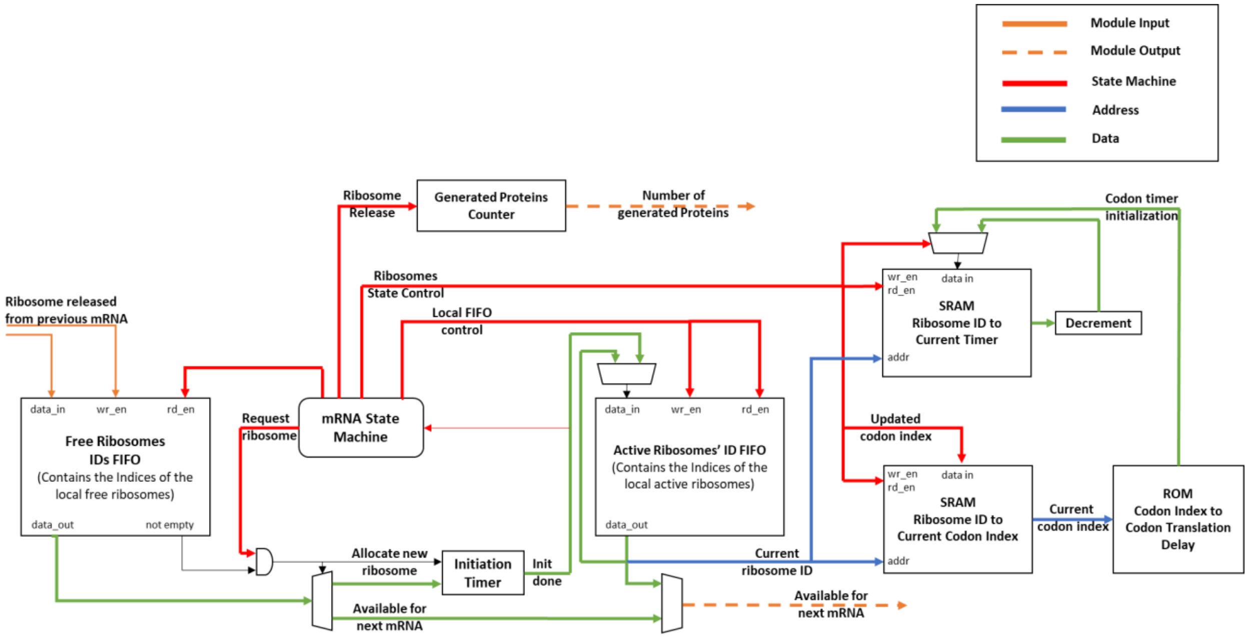

Figure S1 – Initial iterative mRNA module block diagram. As shown, we initially examined a basic approach in which we do not keep a global arbiter for the ribosomes but a local FIFO for each mRNA molecule that is concatenated to the following mRNA in hardware. That FIFO kept the IDs of the current local free ribosomes and the whole state of the ribosomes was managed via tables that map the ribosome's ID to the state.

To model allocation, translation, and diffusion delays, we use timers which are decremented each clock cycle. The initialization value of those timers is chosen by normalizing all delays from seconds to clock cycles. In E.Coli, for instance, each timer decrement models 1ms in "real" cell time. Therefore, when running the hardware with input clock of frequency f(MHz), we get that the timers' decrement phase of the model can potentially take $\frac{1ms}{1/f} = 1ms * f$ seconds. For $f = 100MHz$, we get that the hardware runs 100,000 times faster (in the decrement phase) than a real cell.

Next, the state of the ribosomes is stored in two SRAM memories. Each ribosome is assigned a unique index / address. The first SRAM maps each ribosome to the current codon index along the mRNA molecule. The second SRAM maps each ribosome to the remaining delay time it should wait before advancing to the next codon. Notice, that the allocation and diffusion delays are handled outside the SRAM complex to reduce hardware costs (only one active ribosome can be in the allocation / release state in each time point).

For the initialization of the ribosomes' translation timers, we used a ROM that maps codon index to timer value. That is, when a ribosome advances to the next codon, the state machine should retrieve the next timer initialization from that ROM.

The mRNA state machine constantly iterates over all its active ribosomes and decides the next step. The state machine is also responsible for incrementing the local generated proteins' counter upon ribosome release.

And finally, to form a multiple-mRNAs model, all mRNAs are cyclically concatenated as follows: each mRNA has a local free ribosomes FIFO which is written by the previous mRNA (upon ribosome release) and read by the current mRNA and the next one. By initializing the input FIFO for the entire complex, we control the assigned global number of ribosomes of the model.

By implemented this design, we were able to fit, into the FPGA, 256 E.Coli mRNA molecules with the maximal theoretical storage needed for their ribosomes' states. By doing so, we reached 90% BRAM utilization and 25% LUT utilization with 70MHz input clock.

We than continued by analyzing the utilization report of the design to further improve the utilization in order to be able to fit more mRNA molecules into the design.

2.1 Initial mRNA module – memory utilization analysis

As mentioned, from the utilization report, we got that the utilization of the BRAMs is very high and that is what basically limits the number of mRNA molecules that we can eventually fit into the FPGA device. Therefore, we first approach examining the memory utilization of each mRNA module.

2.1.1 Memory blocks

The following table summarizes the memory usage of the original design for E.*Coli:*

**Codon index to codon delay (ROM)**	
Each mRNA module contains a single-port memory for the codon's translation time. The size of the memory is chosen specifically for each mRNA molecule in the simulation. This is the ROM which contains the map between the codon index and the translation delay.	
**Entry Width (bits)**	**Number of Entries[1] (log)**
**Log(24052**[2]**) = 15**	$Max = Log(2358) = 12$<br>$Min = Log(14) = 4$
**Total size (bits): 60bits – 60Kbits**	
**Active ribosomes' ID FIFO**	
Each mRNA module contains the state of its active ribosomes. The state of the ribosomes is stored in 3 single-port memories. Each ribosome is given an index which is then used as an address to those memories. This FIFO contains the indices of the current active ribosomes.	

[1] Maximal and minimal mRNA lengths are taken from E.coli data

[2] That is the maximal translation time – taken from the E.Coli data

Entry Width (bits)	Number of Entries
15 – to allow up to $\mathbf{2^{15} = 32,000}$ ribosomes in the cell	The number of active ribosomes per mRNA is limited. For an mRNA molecule of size m and minimal distance between ribosomes as D, the maximal number of simultaneously active ribosome is given by: $\left\lfloor \frac{m}{D} \right\rfloor$. In E.coli, this value varies between 4 to 128.
**Total size (bits): 60bits – 1.92Kbits**	
**Ribosome ID to current codon index (RAM)**	
Maps each possible ribosome index to its current codon index.	
**Entry Width (bits)**	**Number of Entries**
The longest mRNA is of size 2358 so we need 12 bits to represent the codon's index.	For the same reason as in the FIFO size, we have between 4 to 128 active ribosomes per mRNA.
**Total size (bits): 48bits – 1.536Kbits**	
**Ribosome ID to current codon timer (RAM)**	
Maps each possible ribosome index to its current codon timer. The timer is initialized from the ROM and decremented for each iteration.	
**Entry Width (bits)**	**Number of Entries**
15 bits – to allow large allocation delays.	For the same reason as in the FIFO size, we have between 4 to 128 active ribosomes per mRNA.
**Total size (bits): 60bits – 1.92Kbits**	
**Free ribosomes' ID FIFO**	
Each mRNA module contains an output FIFO for its released ribosomes. A ribosome index is written to it when it is released from the current mRNA molecule and it is then can be used by the concatenated mRNA.	
**Entry Width (bits)**	**Number of Entries**
15 – to allow up to $\mathbf{2^{15} = 32,000}$ ribosomes in the cell	This number is arbitrary. We need to make sure that the total number of slots in all FIFO allows the maximal number of ribosomes in the cell. Currently, each FIFO contains 4 entries.
**Total size (bits): 60 bits**	

The module's state management is carried out by 4 state machines:

1. *mrna_fsm_main* – responsible for the global mRNA state management
2. *mrna_fsm_alloc* – responsible for new ribosome allocation
3. *mrna_fsm_qry* – responsible for advancing the active ribosomes' ID FIFO pointers.

4. *mrna_fsm_rls* – responsible for releasing ribosomes after translation is done.

The total number proteins generated for the current mRNA is kept in a counter that counts-up for every release event.

2.1.2 Utilization Analysis

Now, let us view the amount of memory bits available:

Part No.	BRAMs	LUTRAM bits	Slice Registers	Ultra RAM	Total
7z020clg484	4.9Mbits	17,400 * 64 = 1.1Mbits	106Kbits	None	6.1Mbits
ZU7EV	11Mbits	6.2Mbits		27Mbits	44.2Mbits

Therefore, without taking into consideration the common logic and state machines, by dividing the total memory resources by the average utilization of an mRNA, we get an upper bound which is much higher than the number of mRNAs than we can actually fit in the synthesis. Actual results for 7z020clg484 for 64 mRNA molecules (taken from Vivado utilization reports):

```
+----------------------------+-------+-------+-----------+-------+
|          Site Type         |  Used | Fixed | Available | Util% |
+----------------------------+-------+-------+-----------+-------+
| Slice LUTs*                | 32784 |     0 |     53200 | 61.62 |
|   LUT as Logic             | 31526 |     0 |     53200 | 59.26 |
|   LUT as Memory            |  1258 |     0 |     17400 |  7.23 |
|     LUT as Distributed RAM |  1258 |     0 |           |       |
|     LUT as Shift Register  |     0 |     0 |           |       |
| Slice Registers            | 14172 |     0 |    106400 | 13.32 |
|   Register as Flip Flop    | 14172 |     0 |    106400 | 13.32 |
|   Register as Latch        |     0 |     0 |    106400 |  0.00 |
| F7 Muxes                   |   297 |     0 |     26600 |  1.12 |
| F[illegible]2. Memory                                          0 |
+---------                                                     --+
```

```
+-------------------+------+-------+-----------+-------+
|     Site Type     | Used | Fixed | Available | Util% |
+-------------------+------+-------+-----------+-------+
| Block RAM Tile    |  100 |     0 |       140 | 71.43 |
|   RAMB36/FIFO*    |    1 |     0 |       140 |  0.71 |
|     RAMB36E1 only |    1 |       |           |       |
|   RAMB18          |  198 |     0 |       280 | 70.71 |
|     RAMB18E1 only |  198 |       |           |       |
+-------------------+------+-------+-----------+-------+
```

Figure S2 – Utilization report taken from Vivado synthesis tool.

The difference between the upper bound and the implementation results can be explained as follows:

1. the calculations do not take into consideration the fragmentation of the data. The fragmentation is important because not all data is stored in the same memory.

2. The amount of combinational logic & routing resources needed to implement a distributed RAM grows as the RAM grows. That is not the case when using a BRAM.

For a matter of fact, by viewing the resource utilization report of the synthesis, one can notice that in most cases, the implementation of the ribosomal state is done using 2 RAMB18 BRAMs.

Moreover, the implementation of the ROM using a BRAM requires a single BRAM18 in most cases. That reduces the maximal amount of mRNA bound to 280 / 3 = 93 mRNAs. That matches the synthesis results.

Therefore, by applying only the BRAM considerations, we get a tighter bound:

Part No.	BRAM18 amount	Max mRNA
7z020clg484	280	280 / 3 = 93
ZU7EV	610	610 / 3 = 203

As mentioned, from the utilization report, we got that the utilization of the BRAMs is very high and that is what basically limits the number of mRNA molecules that we can eventually fit into the FPGA device. Therefore, we first approach examining the memory utilization of each mRNA module.

2.2 Initial mRNA module – memory optimizations

2.2.1 mRNA codons' ROM size

This memory is the largest for a given mRNA molecule. This memory consists of the delay the simulator should wait for each codon in each mRNA.

Due to the fact the translation time of a given codon is independent of the mRNA molecule and only depends on the type of that codon – one can considerably reduce the memory needed for large mRNA molecules.

That can be done using two cascaded smaller memories. One that contains the list of the types of the codons in each mRNA molecule and another that contains the translation between codon type to translation time.

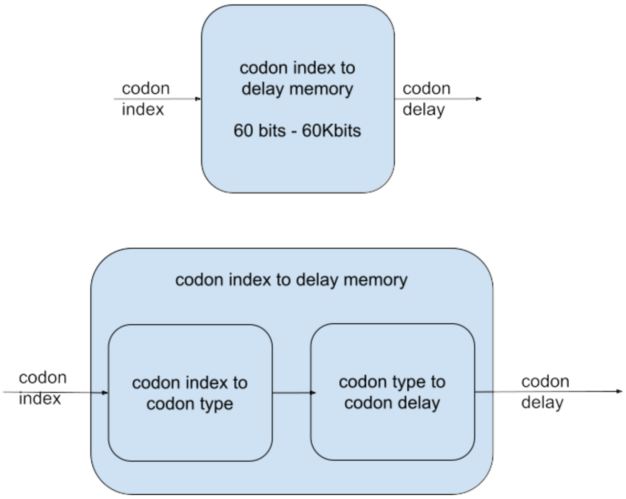

Figure S3 – Storing the codons' data. The upper table maps the codon index to the codon delay. The bottom is an alternative approach in which we keep two concatenated tables that are potentially smaller than a unified single table as in the upper table.

The mRNA molecules in the E.Coli molecule are of length 14 to 2358. Each codon can be coded by 6 bits = 4 possible nucleic acids (2bits) * 3 nucleic acids per codon.

Therefore, the first table is of max size of 4096 * 6 = 24Kbits. The second table is of constant size of 64 (possible codons) * 15 bits per codon delay = 960bits.

That means that for the worst case, this method reduces the required memory needed from 60Kbits to 24Kbits!

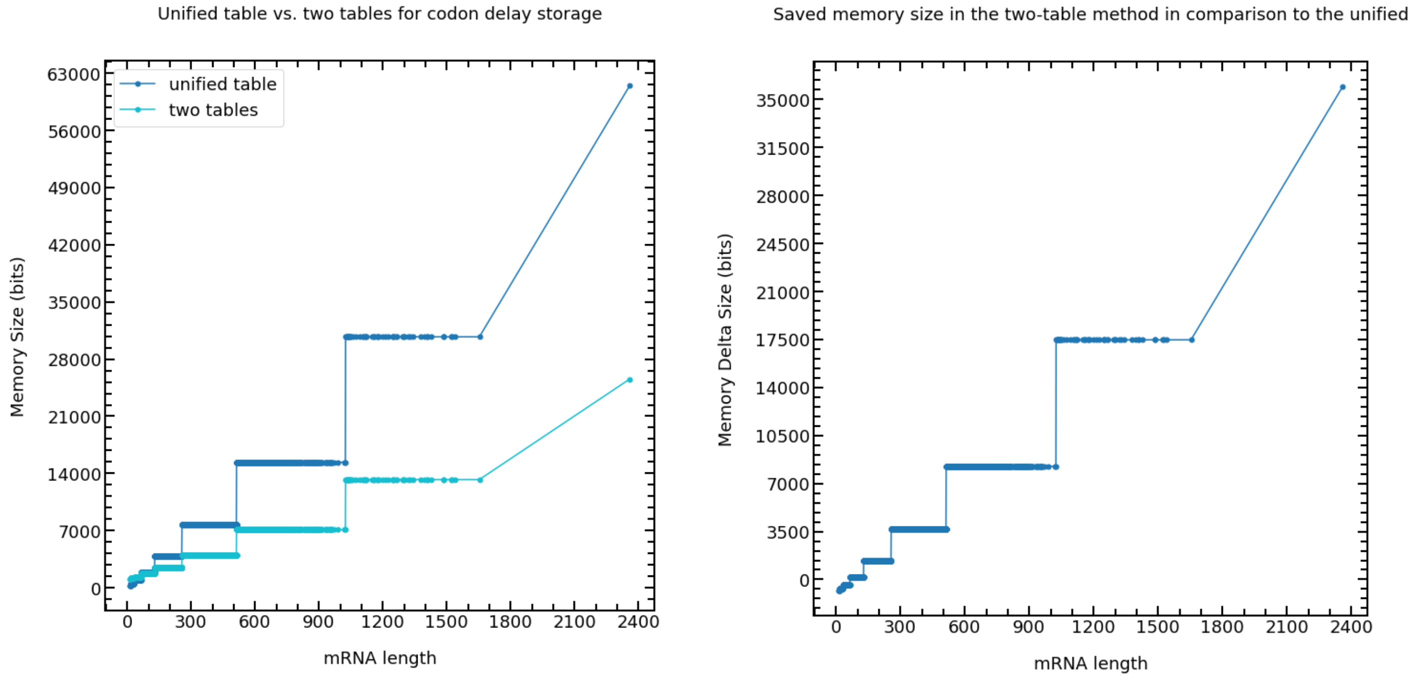

Figure S4 – Left – the memory size in bits required for the two suggested storage methods presented as a function of the length of the mRNA molecule. Right – The saved memory when using the two-table method in comparison to the unified. In this graph we can see that most of the values are positive (i.e. the two-table method improves the memory consumption) and some values are negative (for short mRNA molecules).

One can notice that the size is arranged in steps. That is since the memories are chosen to be of length of power of 2 for hardware efficiency.

When choosing randomly an mRNA molecule from the E.COLI data, those are the probabilities for the required memory size:

Unified Table Memory Size (bits)	Two Tables Memory Size (bits)	Probability
**240**	1056	0.001222195
**480**	1152	0.007577609
**960**	1344	0.021755072
**1920**	1728	0.125152774
**3840**	2496	0.287704718
**7680**	4032	0.429723784
**15360**	7104	0.114397458
**30720**	13248	0.012221951

**61440**	25536	0.000244439

That leads to the following expectation size of required memory to:

Average memory size in the unified method = ***6817.79 bits***
Average memory size in the two-table method = ***3687.11 bits***

That means that the two tables method is more memory efficient by a factor of 1.8 than the unified table method.
Since the ROM storage consumes approximately a third of the BRAMS, we get that by implementing the two-table method we can save up to approximately half of that!
Notice: The codon type-delay table can be easily implemented using 16 O6 LUTs (one for each delay bit) as follows:

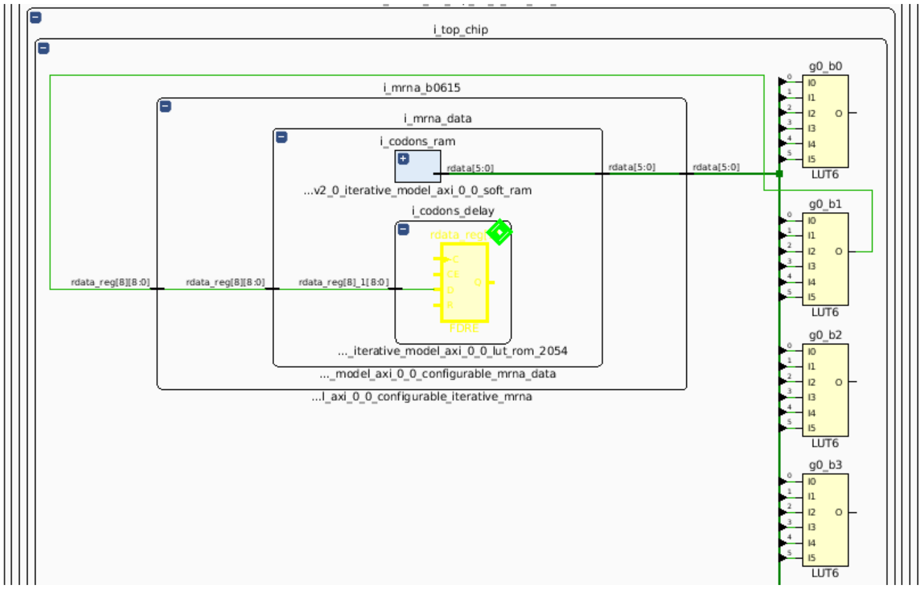

Figure S5 – The LUTROM implementation of the codons' code to codon delay table. This is taken directly from the netlist output of the synthesis. For simplicity, here we show only the four upper LUTs but there are 16 LUTS in total (as expected). In later chapters, we are going to present the lut_rom.v *module in which we used a special synthesis directive to have the synthesis implement this module using LUTs.*

In this figure, we can see that the address for the actual value of the codon is mapped inputed to 16 LUTs (in the figure we only see the first 4 LUTs). The figure is taken from the synthesis results using Vivado.

2.2.2 Ribosomes' state size

One can notice that the maximal number of ribosomes working on the same mRNA simultaneously is given by (mRNA length) / (minimal distance between consecutive ribosomes). The minimal distance must be preserved due to the size of the ribosome. For E.Coli molecules, a single ribosome occupies 9 codons. That is, the minimal distance between consecutive ribosomes is 9 units.

That means that the ribosomal state tables are bounded in length by the mRNA size divided by the minimal distance. In the current design, the size of the tables is fixed for the maximal possible value for all mRNAs.

Unfortunately, this size cannot be reduced separately for each mRNA to fit its needs because of the indexed matter in which ribosomes are handled. In fact, the ID of a given ribosome is not important. Therefore, if we could only save the index of the ribosome relative to the current mRNA, that would save a lot of space.

That can be accomplished by using a cyclic FIFO and a counter instead of the fixed-size memories.

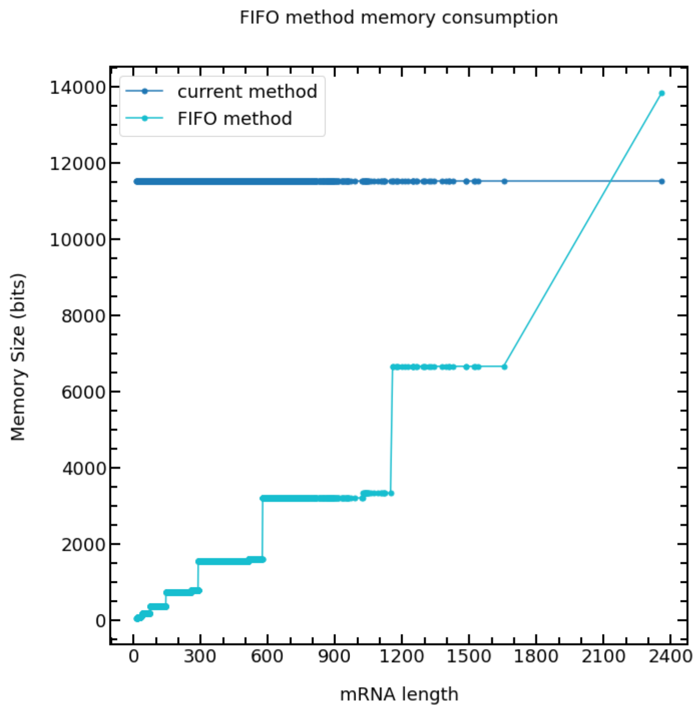

Figure S6 – This graph presents the memory consumption of the original method for the ribosomes' state storage (the constant line) and the new improved memory consumption in the FIFO method. Notice that for the original memory consumption graph we neglected the longest mRNA since there is a large gap in the mRNA lengths.

Notice that the last mRNA (the longest) is neglected. This graph suggests a significant improvement of the memory required for the ribosomal storage when using the suggested FIFO method.

Memory Size (bits)	Probability
**38**	0.001222
**40**	0.000733
**80**	0.006844
**84**	0.002689
**168**	0.019066
**176**	0.0154
**352**	0.109753
**368**	0.031777
**736**	0.255928
**768**	0.068198
**1536**	0.361525

**1600**	0.037399
**3200**	0.076998
**3328**	0.004889
**6656**	0.007333
**13824**	0.000244

Average memory size in FIFO method = ***1227.8235150329995 bits***
Average memory size in original method = ***11520 bits***
Notice, that for each mRNA molecule, the width of the width of the codon index is different and depends on the length of the mRNA molecule. That is also taken account in the above results.

That means, that by implementing the FIFO method, we can save up to 90% of BRAMs in comparison to the current implementation.

2.3 Initial mRNA model - Inaccuracies

2.3.1 mRNA state machine bias

It is easy to see that as in this model, the mRNA state machine iterates over all active ribosomes, the mRNA state machine latency depends on the number of its active ribosomes. That latency is not taken into consideration and causes more occupied mRNAs to generate proteins in a lower rate in the model although that may not be the case in real cells.

First, let us examine the influence of the state machine delay via simulation. Following, are the simulation waveforms generated from simulating a mRNA molecule being translated by one, two and three ribosomes. The captures focus on a single delay counter decrement.

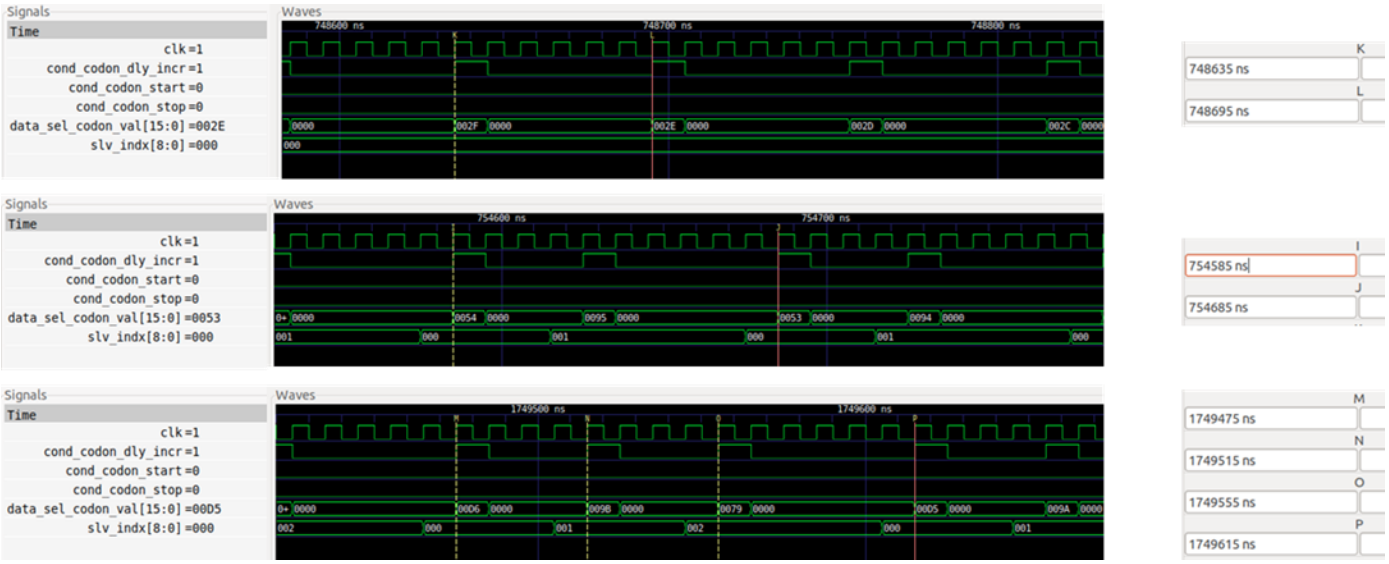

Figure S7 – In this figure we see the simulation wave diagram of some selected signals to demonstrate the bias that is introduced by the state machine. **data_sel_codon_val represents the current value of the translation timer. In each figure, we examine the time in nanoseconds that it takes to decrement the timer. In the upper figure, which is generated for the case one active ribosome, we see that the timer is decremented from 0x2f to 0x2e in 60ns. In the middle figure, for two active ribosomes, the timer is decremented from 0x54 to 0x53 in 100ns. In the bottom figure, for three ribosomes, the timer is decremented from 0xd6 to 0xd5 in 140ns.**

Those captures show that a single timer decrement of a codon in a mRNA molecule takes approximately:

$$40r + 20ns$$

When r denotes the current number of active ribosomes on the mRNA molecule. The 20 ns constant comes from the state machine delay that is common to all ribosomes.

That shows that the state machine delay is not neglectable at all and causes a huge bias to the entire system.

In fact, that dependency is linear, meaning that, the time between two consecutive decrements of the same ribosome ($\Delta t_{decrement}$) is proportional to the current number of the mRNA's active ribosomes (r):

$$\Delta t_{decrement} = c * r + const$$

By analyzing the simulation of our hardware model, we had $\Delta t_{decrement} = 4 * r + 2\ clock\ cycles$. That demonstrates that current design's state machine delay is not neglectable in comparison to the codon translation delay. This delay also causes a distortion in the model's timing – ribosomes will be released in hardware long after they are released in "real" cell when they are operating on mRNAs with lots of other active ribosomes.

Also, that introduces another issue - causality –the entire simulator is not casual – the "current time" of each mRNA is different. When requesting a ribosome regardless of the current time, the mRNA can receive a ribosome that has not yet released in "real time".

<u>mRNA synchronization</u> – one can consider synchronizing the state of all active mRNA molecules in a manner that whilst a given molecule has not done iterating over all its ribosomes, the other mRNA does not advance.

Intuitively, this approach is inefficient because it causes plenty hardware idle times. Moreover, the idle periods are more frequent when we model more mRNA molecules.

Although the method of mRNA synchronization might impact the performance of the simulator, it helps with the causality issue – when the state of all mRNAs is synchronized – the system is causal.

<u>Adding current time to the mRNA state</u> – we can add a time counter to the state of each mRNA molecule. The counter should contain the dT passed from the beginning of the simulation. The counter should be advanced only upon completion of the state machine's iteration.

In this way, we will be able to divide the generated protein's counter by the dT of that mRNA molecule to receive the actual generation rate. Those changes were taken into consideration in the later versions of the iterative model presented in the article.

We also considered the following approaches:

<u>Halt and advance the slowest</u> – occasionally, when the dT of the current time is large enough between mRNA molecules in the simulator – halt the fastest mRNAs and advance the slowest.

Regardless of the implementation in hardware (which might be extremely complicated when having multiple mRNA molecules), it might not solve the problem – longer mRNA molecules are more likely to have more ribosomes and therefore might be consistently slower than all other mRNAs. That might cause frequent halts.

Match time in pairs – instead of halting the entire system to approximate causality, we can halt only consecutive pairs in the following way:

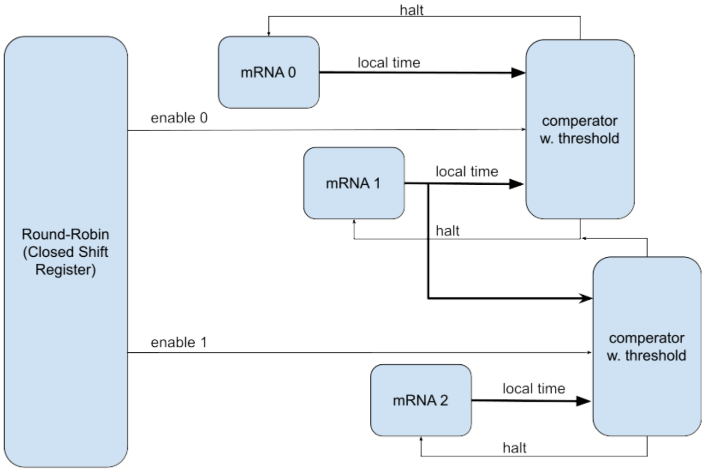

Figure S8- block diagram for the match in pairs synchronization method. We have a cyclic shift register with only one set bit which cyclically generates the enable signal for each comparator. The comparators are used to match the current time of each mRNA molecule by halting the fastest mRNA when the time difference exceeds a parametric threshold.

In this way, only one comparator is active in each time. That results at most in only one halted mRNA molecule in each time. The threshold input to the comparator can also determine the dT inaccuracy that we can tolerate.

In practice, this solution is less accurate than the mRNA synchronization solution. In the final iterative model presented in the article, we used the mRNA synchronization method, and the performance speedup was quite sufficient for our need. Implementing the match-in-pairs method should be considered if we can tolerate a less accurate model that runs faster.

2.3.2 Ribosomes' allocation bias

The concatenation of the mRNA ribosomes' FIFOs causes a bias in the free ribosome allocation probability. The first mRNA molecules will always receive a ribosome molecule at the beginning of the simulation. Moreover, when a ribosome is released, the next mRNA molecule will have priority in receiving it. In a real cell, the allocation of free ribosomes happens randomly with uniform probability. The assumption was that in the steady state, the variance in the mRNA lengths will cause enough randomness so that the free ribosomes will be distributed uniformly among the mRNA molecules and this bias will not be noticeable.

In fact, by analyzing the simulation results, the bias of the ribosomes allocation is not neglectable and shown in the following graph:

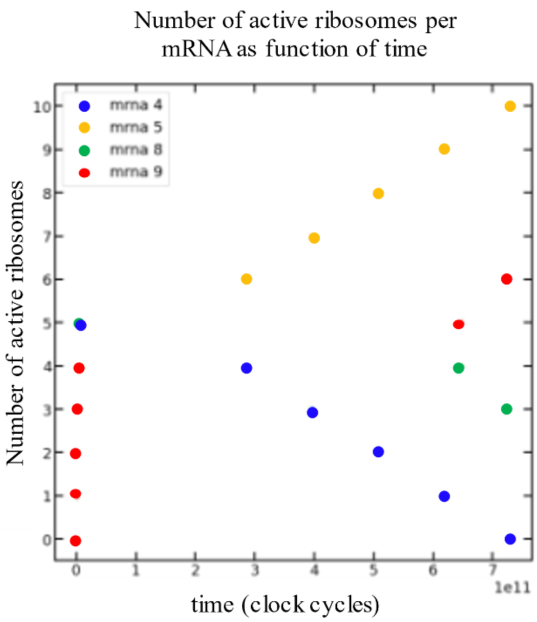

Figure S9 – This figure represents the amount of active ribosomes on each mRNA molecule as function of time in the initial model for selected mRNA molecules. The module time is in nano seconds. This graph is generated by parsing the ".vcd" simulation output of the initial model. The graph clearly shows the bias of the ribosome allocation probability towards consecutive mRNAs.

In this graph, one can notice that the ribosomes released from mRNA 4 are immediately caught by the concatenated mRNA (5). That also happens in the 8,9 pair. This graph shows clearly that the current ribosomes allocation favors the consecutive mRNA molecules and is far from being uniform.

One can notice that:

1. The ID of each ribosome is not important.
2. In real cells, the ribosome allocation to a pending mRNA is random.

The current implementation uses a concatenated FIFO architecture to propagate free ribosomes by ID from one mRNA to another. That results in an inefficient memory utilization (as shown in the utilization chapter) and an inaccurate model (due to a bias in the ribosomal allocation).

After realizing that bias, we were able to come up with the new approach of having a global ribosomes' arbiter which is brought in the paper:

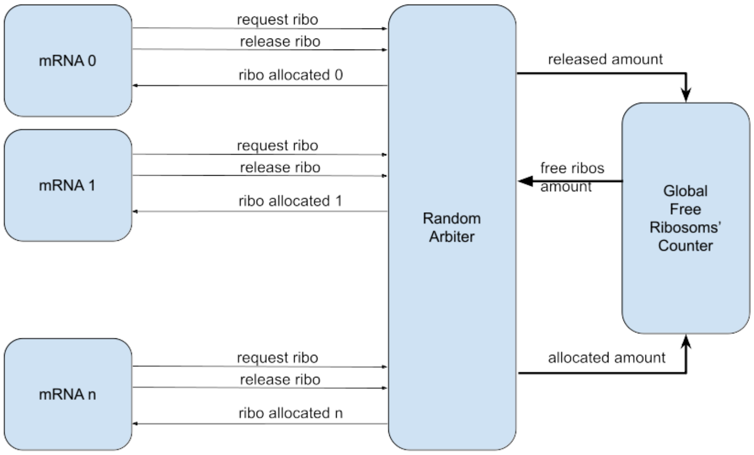

Figure S10 – The high-level architecture of the connections between the global arbiter and the mRNA molecules. Like the block diagram that is presented in the main text.

The round-robin arbiter is very similar to the initial design as it favors consecutive mRNA molecules. The uniform arbiter, that was introduced, fixes this bias entirely.

2.4 Initial Design – Conclusions

For conclusion, here is the list of the insights that we got from the initial design analysis:

1. In case we have a memory bottleneck, we should consider storing the codon delays in two separate concatenated memories instead of a single large one. That was useful in the iterative model and as shown in the main text, the codon's data is stored in two concatenated memories. In the parallel model, the bottleneck was logic utilization and not memory utilization. Therefore, in the parallel we kept the single large memory for the codon data for the state machine simplicity.
2. Working with absolute indexes for the ribosomes leads to a large memory consumption. The ID of the ribosomes is not important for the sake of mRNA translation modeling. Therefore, in the iterative model, we kept the ribosomes' state inside a FIFO and the only thing that we kept track of was the number of active ribosomes (and their order – which is enforced by using a FIFO).
3. When having autonomous separate mRNA molecules, it is important to make sure that the local time of each mRNA molecule is synchronized with all other mRNAs. In the parallel model it is given by having the system run simultaneously in parallel and not iteratively. In the iterative model, as shown in the article, we synchronized all mRNA state machines by adding a "hold" signal that releases only if all mRNAs are done with the current iteration over their ribosomes.
4. We should embrace the global arbiter architecture to avoid bias in the ribosomes' distribution among mRNA molecules.

3 PARALLEL MODEL – ADDITIONAL INFORMATION

The general idea is to avoid iterating over each active ribosome of a single mRNA molecule. Instead, we shall try having the ribosomes act as independent hardware entities. As suggested above, the mRNAs are connected to a global arbiter. The arbiter receives the request & release signals of the mRNAs and generates grants the ribosomes. The arbiter in the new design contains the global counter of free ribosomes. Here is an example for 4 mRNA molecules from the synthesis tools:

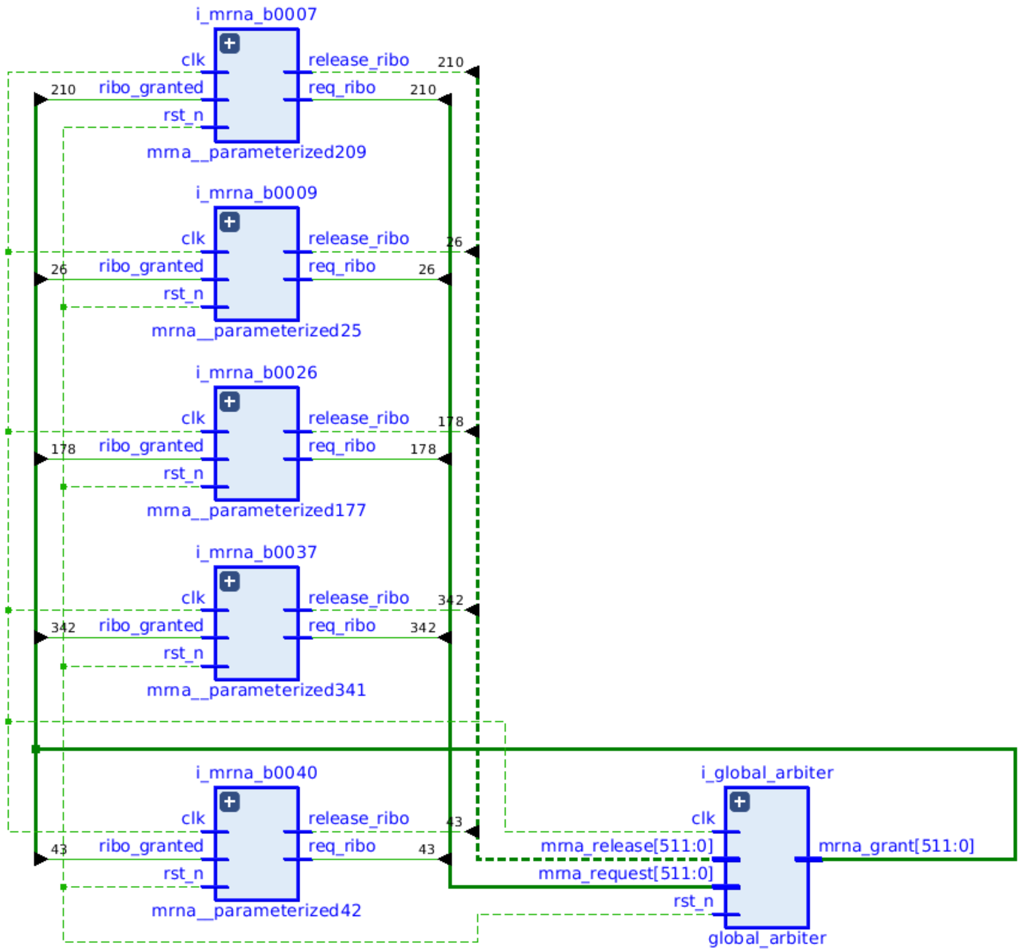

Figure S11 – This is an illustration generated by Vivado synthesis tools based on the HDL of the parallel model. Here we present the connections between the mRNA modules and the global arbiter. The same architecture is later used in the iterative module.

Notice: here, each mRNA raises the release signal for a single clock cycle when a ribosome is released. Therefore, the global arbiter logic should count the release events until flushed.

Next, each mRNA molecule, contains a concatenated structure of hardware implemented ribosomes.

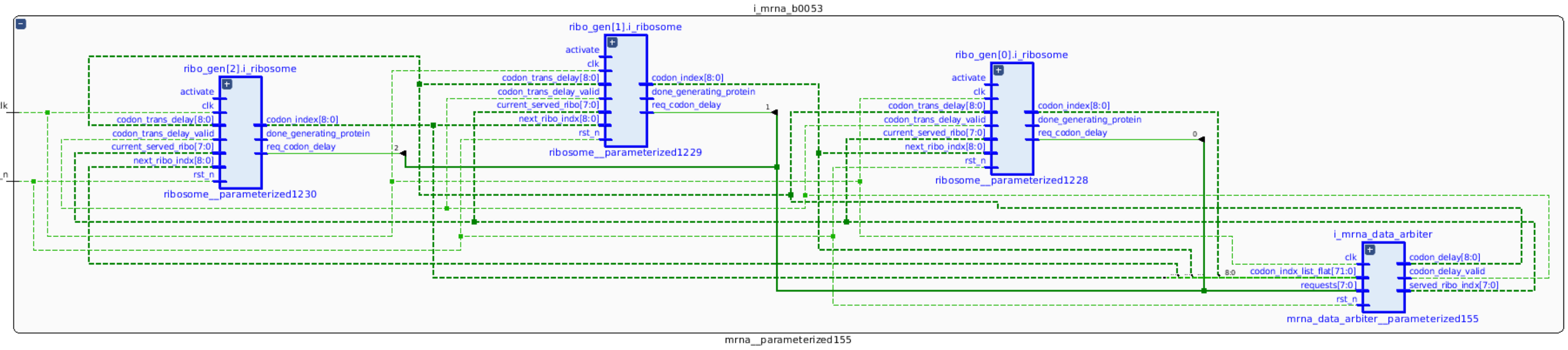

Figure S12 – This is also generated by Vivado based on our HDL. In this figure we see the connections between the hardware ribosome (here we present three of them) and the global data arbiter.

Each ribosome has its own state machine. Each ribosome is connected to the index of its consecutive ribosome (to make sure it can skip to the next codon when done counting). The last ribosome in the hardware structure is connected to the index of the first in a cyclic manner.

One can think of the concatenated ribosomes as a hardware ribosome FIFO. First, all the ribosomes start inactive and when the global arbiter assigns a new ribosome, the mRNA logic is responsible for activating a ribosome in the hardware.

The mRNA logic is responsible for keeping the following values:

1. Read pointer – a pointer to the first current active ribosome.
2. Write pointer – a pointer to the first inactive ribosome – when a new ribosome is granted by the global arbiter, the one pointed by the write pointer is activated.
3. First pointer – a pointer for the first active ribosome from the 5' end of the mRNA molecule – that is kept for the mRNA logic to determine if a new ribosome request should be issued.
4. Active ribosomes counter – to make sure that the hardware ribosomes FIFO does not saturates.

Then, we have all the ribosomes run freely and maintain the minimal distance from each other. To get the wait time for a given codon index, we could have kept a local BRAM memory for each ribosome in hardware. That is not hardware efficient since the hardware ribosome spends most of its life counting. The event of skipping to the next codon occurs far less than timer advancement.

Therefore, we shall have the mRNA codon delay table in a common place accessible for all hardware ribosomes. This memory should be managed by an arbiter. We implemented it by a simple round-robin arbiter. The arbiter iterates over all ribosomes' indexes (including the inactive ones) and outputs the delay value. The ribosomes contain a comparator that constantly examines if their index is the one being served[3].

[3] Instead of having the arbiter signaling each endpoint – that would have cost more hardware.

The state machine of each ribosome is given by:

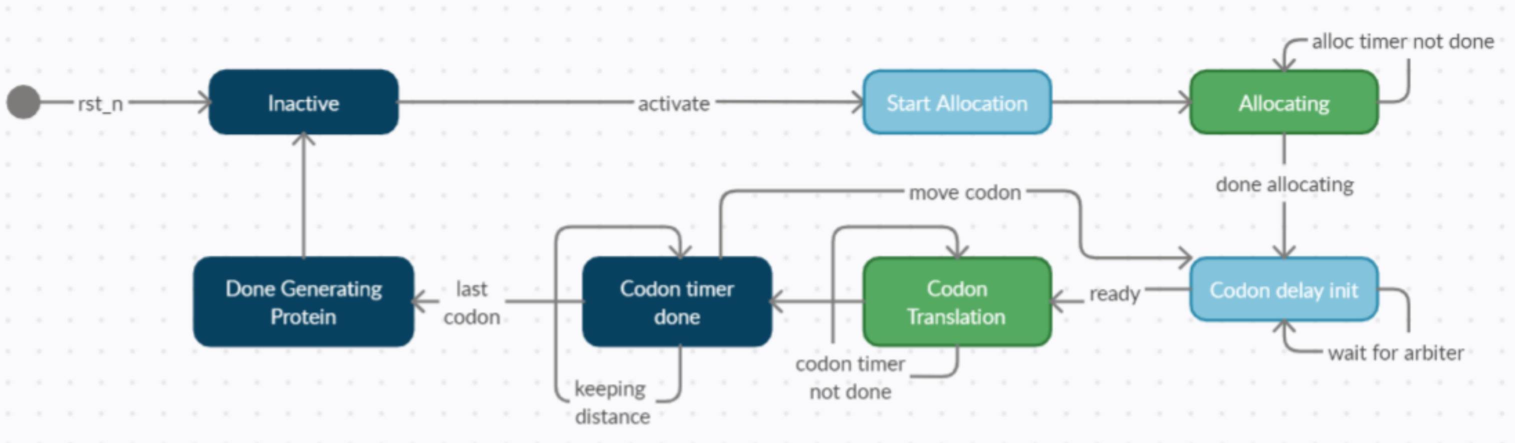

Figure S13 – The ribosomes' state machine. It begins in the inactive state (which is basically and idle state) and then continues to the allocation states when it receives the activate *signal from the mRNA module. Next, after the allocation timer is done (*done_allocating*), the ribosome continues with translating the codons. It is done by first retrieving the codons' delay from the data arbiter (*codon delay init*) and then advancing the translation timer. When the translation timer reaches 0, the ribosome waits until the concatenated ribosome is far enough (*keeping_distance*). When the index of the current codon equals to the mRNAs length, the ribosomes is done (*done generating protein*).*

3.1 First Results

We have implemented the parallel design using the Zynq Ultrascale+ chip. To feat in as many mRNAs and ribosomes as possible, we had to make some adjustments. First, notice that the true bottleneck of the entire design is the size of a single hardware ribosome. The internal state of the ribosome is not expected to be different than in size from the iterative design. As before, we must keep for each ribosome:

1. The index of the current codon
2. The remaining allocation time
3. The remaining translation time
4. The state (allocating / counting / advancing / done)

The difference in the parallel design is that each ribosome is implemented in hardware and run autonomously. That means that we also must keep that state machine logic & adders for each ribosome.

Also, each mRNA molecule (as before) should keep a buffer of hardware ribosomes. Theoretically, the buffer size for a mRNA molecule of size M and ribosome size D (minimal distance) is $\frac{M}{D}$. Although that is the theoretical bound, the practical value of maximal ribosomes active on a single mRNA depends on the global availability of ribosomes in the cell.

Let us denote the global number of ribosomes as R and the number of mRNAs in the cell as N. Then, if the ribosomes' allocation happens uniformly, it is not probable that a single mRNA molecule will have substantially more than $\frac{R}{N}$ **active** ribosomes[4].

---

[4] In the "methods" section we examined the dependency on the length of the mRNA molecule with respect to others in the simulation

So, the size of the hardware ribosomes FIFO for each mRNA molecule was first chosen to be: $\min\left(\frac{M}{D}, \frac{2R}{N}\right)$.

Moreover, notice that **at a given time, only one ribosome can be in the allocation state**. That means that if the allocation state machine logic & allocation timer are substantial, we can consider extracting the allocation from the internal ribosome state machine to the mRNA module level. The resulting state machine of the ribosome will then be:

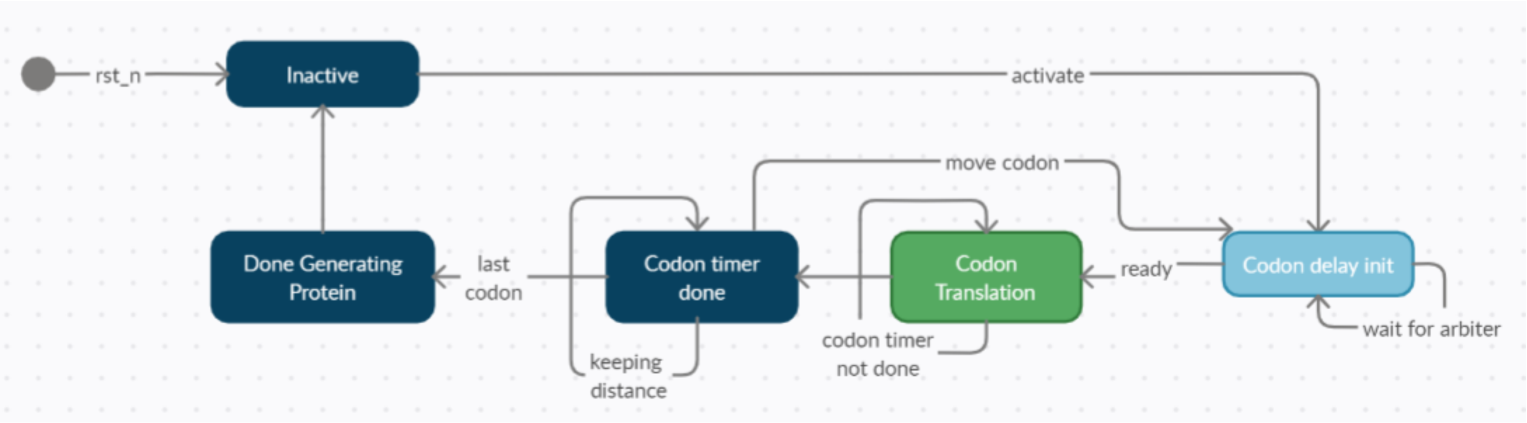

Figure S14 – like the previous brought state machine with the same states and signals except the allocation states that are completely removed. That is done by placing the allocation logic as part of the mRNA module to save resources as shown in this chapter.

Let us examine how substantial is the allocation logic by comparing the LUT and FF consumption of each ribosome before and after extracting the allocation logic to the mRNA module:

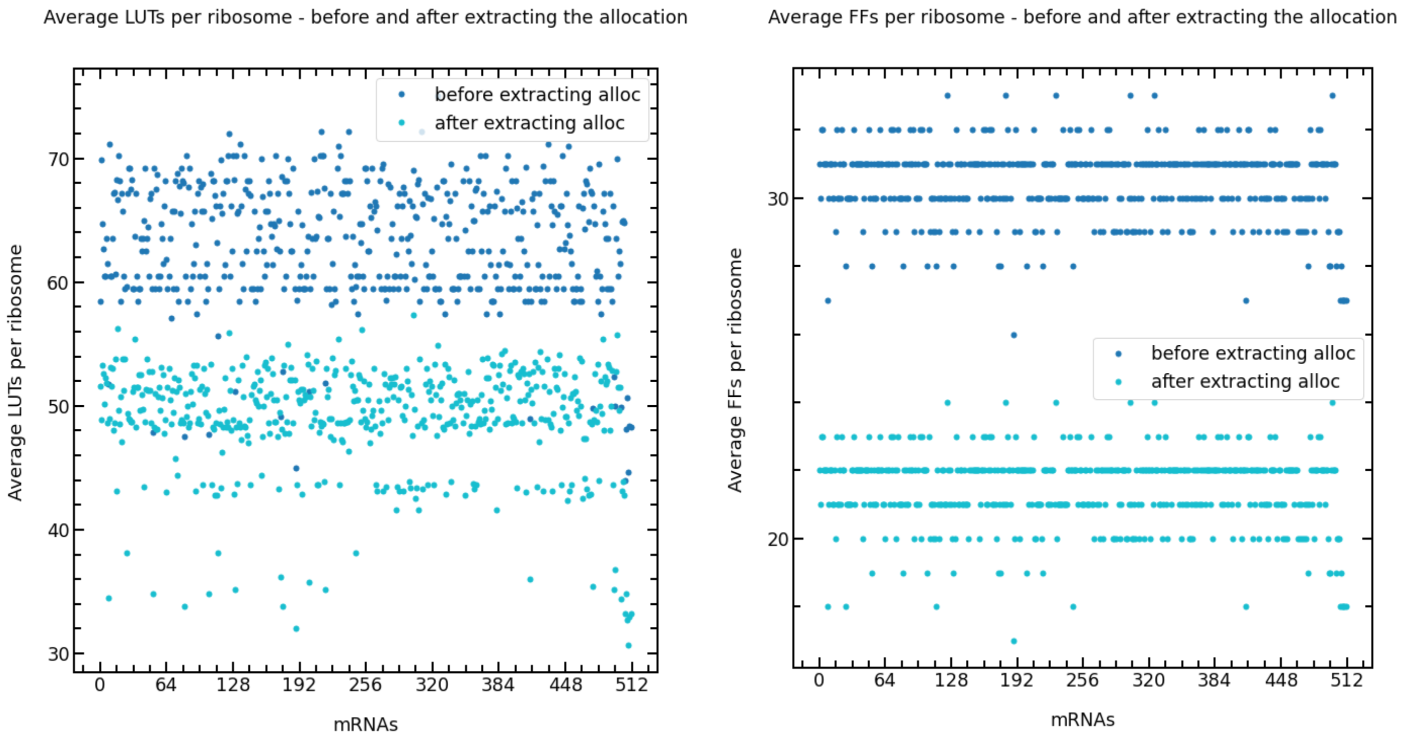

Figure S15 – Left – shows the average LUT consumption per ribosome per mRNA molecule before and after removing the allocation logic to the mRNA module. Right - The same but for the flip flops consumption (FF).

From the graphs[5], we can see that removing the allocation logic can save 25% LUTs and 30% FFs on average. That is substantial enough.

And finally, we have noticed that the synthesis chooses to implement the mRNA memories (the codon delay tables) as distributed RAM instead of BRAMs. To make sure that the BRAMs are used, we have used the directive:

```
(* ram_style = 'block' *)
```

Before the memory instantiation.

After applying all these changes, we were able to squeeze successfully into the design:

1. 512 mRNA molecules
2. 2048 free ribosomes
3. Approximately 4096 hardware ribosomes (according to the above formula)
4. 100 MHz input clock

Here is an illustration of the FPGA (created using Vivado). Each pale-blue cube represents a used resource.

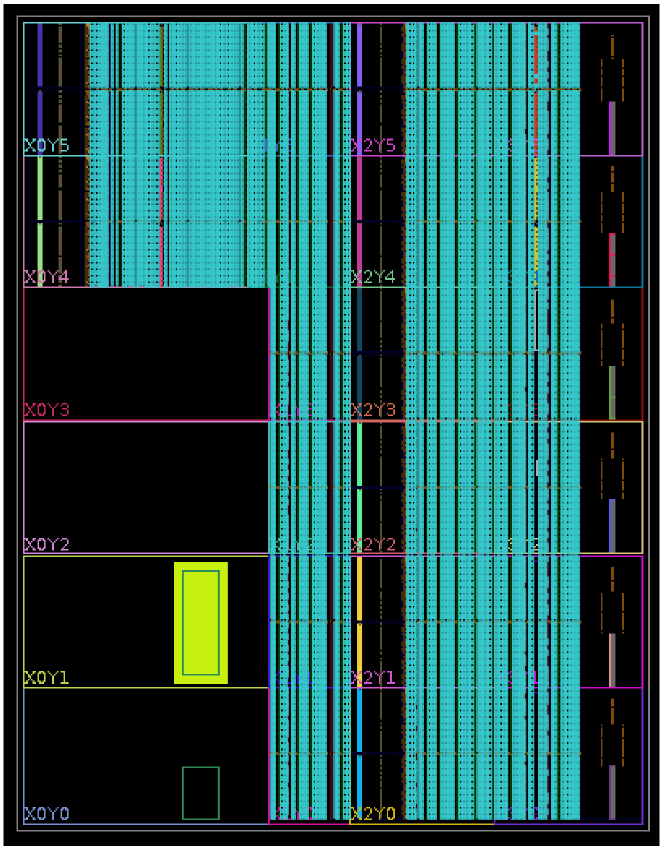

Figure S16 – This figure is also generated by Vivado tool chain after implementation of the parallel design. This figure shows that the device utilization is high as each light-blue cube represents a used slice.

[5] The graphs are made using the hierarchical utilization report of Vivado (of the design before and after remove the allocation logic).

Later, after pipelining the multiplexers of the global arbiter we were able to raise the operation frequency to 200MHz.

3.2 UTILIZATION

Important parts from Vivado utilization report:

CLB utilization:

Site Type	Used	Fixed	Available	Util%
CLB LUTs	220857	0	230400	95.86
LUT as Logic	220857	0	230400	95.86
LUT as Memory	0	0	101760	0.00
CLB Registers	117723	0	460800	25.55
Register as Flip Flop	117723	0	460800	25.55
Register as Latch	0	0	460800	0.00
CARRY8	8442	0	28800	29.31
F7 Muxes	2884	0	115200	2.50
F8 Muxes	289	0	57600	0.50
F9 Muxes	0	0	28800	0.00

CLB logic distribution:

Site Type	Used	Fixed	Available	Util%
CLB	28791	0	28800	99.97
CLBL	16076	0		
CLBM	12715	0		
LUT as Logic	220857	0	230400	95.86
using O5 output only	4219			
using O6 output only	143010			
using O5 and O6	73628			
LUT as Memory	0	0	101760	0.00
LUT as Distributed RAM	0	0		
LUT as Shift Register	0	0		
CLB Registers	117723	0	460800	25.55
Register driven from within the CLB	102949			
Register driven from outside the CLB	14774			
LUT in front of the register is unused	2375			
LUT in front of the register is used	12399			
Unique Control Sets	7105		57600	12.34

Block RAM Utilization:

Site Type	Used	Fixed	Available	Util%
Block RAM Tile	256	0	312	82.05
RAMB36/FIFO*	0	0	312	0.00
RAMB18	512	0	624	82.05
RAMB18E2 only	512			
URAM	0	0	96	0.00

From those tables we can see the LUTs are maxed out for logic and that it was a beneficial choice to implement the mRNA data as BRAM. That is the opposite from the initial iterative design, in which the bottleneck was the memory consumption.

4 SYSTEM PROOF-OF-CONCEPT DETAILS

As shown in the main text, for the system POC we used the iterative model. The connectivity of the system is as follows:

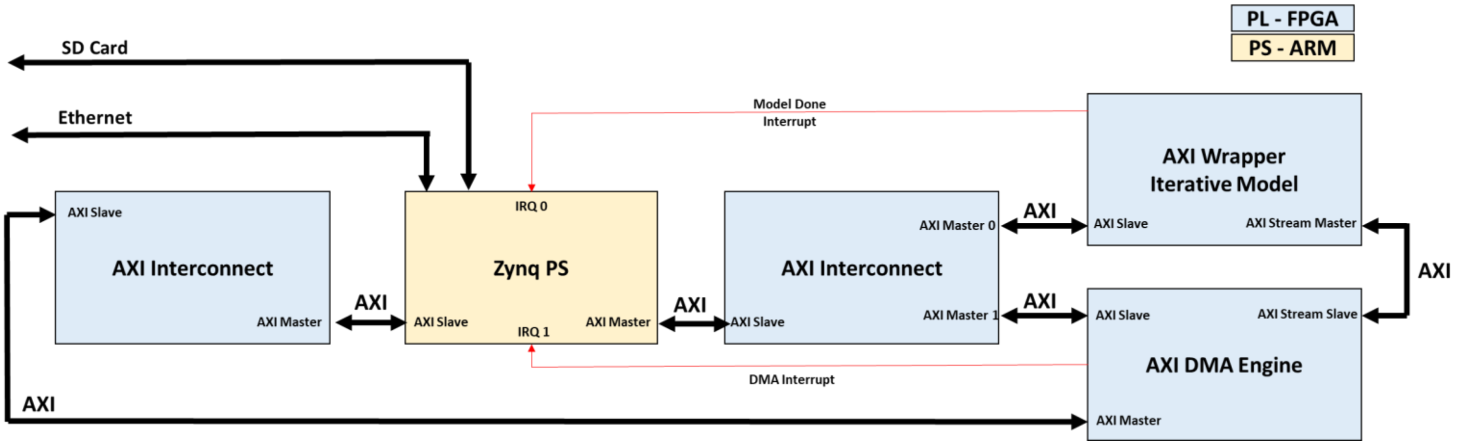

Figure S17 – This figure shows the high-level connectivity inside the Zynq chip that is used for the POC. In yellow – the CPU complex parts (ARM), in blue – the FPGA parts.

4.1 ZYNQ PS

The Zynq PS is basically the CPU complex in which there are few ARM cores and some important embedded peripherals. Here is a block diagram of the Zynq PS:

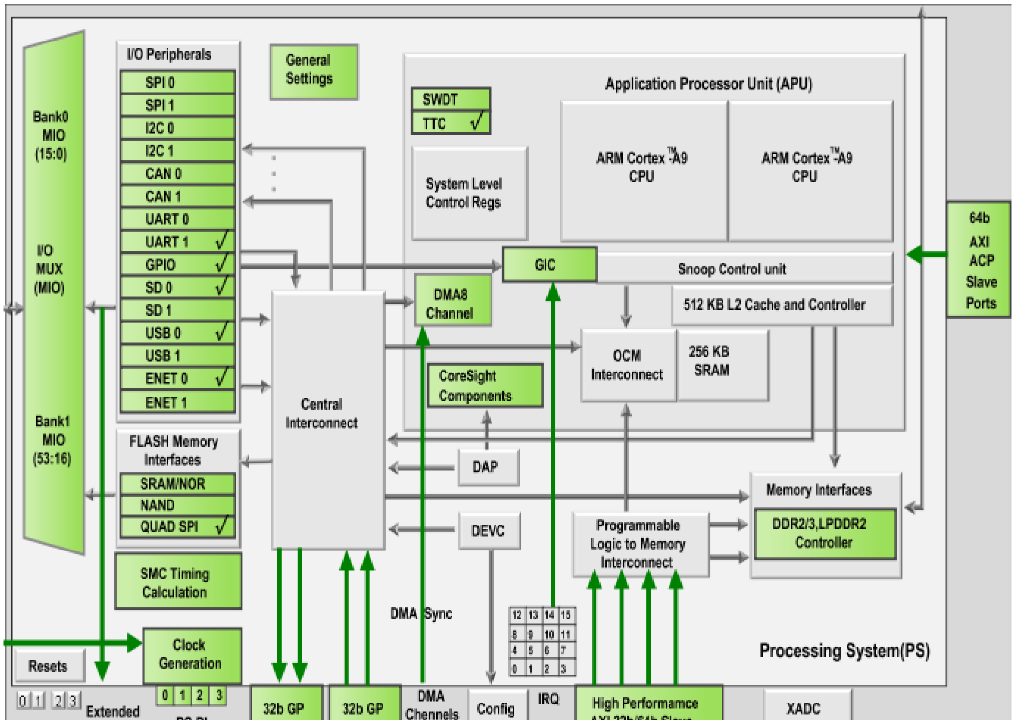

Figure S18 - This figure is also taken from the Vivado tool chain and is used to illustrate the internal blocks inside the Zynq processing system (PS). The PS contains the ARM cores alongside some embedded peripherals to interface with the FPGA (PL) and the board components (such as Ethernet ports, SD card, UART, etc.).

When configuring the Zynq PS subsystem, it is important to enable the required peripherals for the to allow the ARM core the access to the relevant access. For the POC we needed the following interfaces:

1. SD – the compiled Linux, the drivers and the python scripts are all part of the image loaded into the SD card from which the board boots. Also, during runtime, the python optimization algorithms write the logs to the file system stored on the SD.
2. UART – to follow the boot process of the platform – the log output of the boot process is output to the UART port. The UART of the Zynq chip is connected to an FTDI device which translates the communication to USB.
3. Ethernet – In the POC we are running Linux on the ARM cores of the Zynq chip. The Ethernet port is useful for SSH and file transfer to / from the Zynq. The Ethernet is also required for the connection of the board to the network so we can access the later install Jupyter Notebook server that runs on the ARM cores.

Also, the Zynq PS is also responsible for the management of the AXI interfaces to / from the FPGA and the clocks that are supplied to the FPGA. For the POC we used two different clocks that had to be configured in the Zynq PS:

1. Slow communication clock – 100 MHz – that is the clock used for the AXI interfaces between the FPGA and the ARM cores. Therefore, this clock is also used for running the internal communication related registers' logic that is later described.
2. Fast model clock – 200 MHz – that is the clock used for running the model itself. As the communication is not the bottleneck, it is important that the model itself would run as fast as possible.

And then, we have the following AXI interfaces connected to the Zynq PS:

1. AXI Master – this port is than connected to an AXI interconnect module which is responsible for splitting this port to two AXI Slave ports:
   a) Iterative model AXI wrapper – this port is connected to the AXI wrapper that contains the iterative model within. The AXI wrapper is responsible to manage the configuration registers that are used for the communication between the ARM processor and the iterative model.
   b) DMA engine AXI Slave port – that port is useful for configuring the DMA engine in the hardware. Via this port, the destination address in the DDR memory is configured and the DMA engine is enabled.
      The DMA engine was tested as part of the system but for the iterative model it is not necessary since the register interface works fast enough.
2. AXI Slave – This port is connected to the AXI Master port of the DMA Engine (via AXI Interconnect module). Via this port, the DMA engine can access directly to the destination address in the DDR.

And finally, the Zynq PS also has two interrupt ports connected to it:

1. Model done interrupt – this interrupt comes from the iterative model wrapper. When the local time of the model reaches the configured required stop time, this signal goes high and raises an interrupt for the ARM core.

2. DMA done interrupt – when the iterative model finished writing the frame of data to the DMA engine via the AXI Stream interface, the DMA engine (if enabled) writes the data to the DDR memory and then raises an interrupt to signal the CPU core that the transaction is done.

4.2 Using Xilinx PYNQ

For the sake of running python algorithms over Linux OS that is compiled specifically for the ZYNQ chips, Xilinx have developed a dedicated software environment called PYNQ. That is a Linux based software that allows all the basic features of Linux on top of having a comfortable Python Jupyter Notebook server and a dedicated Python package named also PYNQ.

For running this environment on the ZCU104 board, one can simply download a precompiled version from Xilinx's website or to compile it. For our case, we found that the precompiled version for our board contained several bugs, so we had to fix them and compile our own version.

The generated PYNQ image was then programmed to an SD Card (which also included a filesystem partition). Then, after configuring a static IP for the ZCU board (over the UART interface) and for the connected PC port, we where able to connect to the Jupyter server or to SSH directly to the Linux on the board.

To program the FPGA with our bitstream, we had to use the following lines at the beginning of our Python code:

```
from pynq import Overlay
overlay = Overlay("<path to the bitstream file>")
```

It is important to notice that on the same directory in which the bitstream is located, one should also put the ".hwh" file which configures the software environment for the PYNQ package to work properly. This file is automatically generated by Vivado as part of the regular synthesis process.

4.3 Registers interface

As explained, the Zynq core communicates with the FPGA via the AXI interface. This interface eventually reveals a set of configuration registers that are used for activating the model and reading back the results. We created a compact list of registers for the configuration. The base address of our module in the ARM memory space is 0xA000000. The interface registers are defined as follows:

Register name	Register Address	Direction
**CTRL_REG**	**0xA0000000**	**Write**
0: model_rst_n – reset the iterative model.<br>1: model_config_rst_n – reset the model configuration.<br>2: model_config_enable – enable the model configuration.<br>3: stop_time_config_enable – enable the stopping time configuration.<br>4: clear_interrupt – when written 1, the iterative model clears the interrupt.		
**MRNA_CONF_ADDR**	**0xA0000004**	**Write**
[31:16] – mrna_index – the index of the addressed mRNA for codon configuration<br>[15:0] – codon_index – the index of the configured codon in the pointed mRNA		
**MRNA_CONF_DATA**	**0xA0000008**	**Write**

[5:0] – new codon code to be written. **Notice**: this value is only written if the model_config_enable is on.		
**MODEL_STOP_TIME**	**0xA000000C**	**Write**
[31:0] – the stop time in milliseconds – when the iterative model finish modeling the specified number of milliseconds, the model_done interrupt goes high.		
**PROT_MRNA_ADDR**	**0XA0000010**	**Write**
[31:0] – the index of the mRNA from which we wish to read the protein counter. Notice that in practice, only the last 10 bits are used because we currently support 1024 mRNAs.		
**MODEL_REAL_TIME**	**0XA0000014**	**Read**
[31:0] – the current model time in milliseconds.		
**PROT_CNTR**	**0XA0000018**	**Read**
[31:0] Contains the protein counter of the mRNA pointed by PROT_MRNA_ADDR.		
**SEL_MRNA**	**0XA000001C**	**Read**
[31:0] Connected to the internal pipelined mux of the protein readback. Therefore, it is important to compare this value to PROT_MRNA_ADDR before reading the PROT_CNTR register.		
**STATUS_REG**	**0XA0000020**	**Read**
0: model_done – that bit has the same value as the interrupt signal model_done		
**CURR_FREE_RIBOS**	**0XA0000024**	**Read**
[31:0] – The number of currently free ribosomes in the cell.		
**MAX_CELL_RIBOS**	**0XA0000028**	**Write**
[31:0] – assign the total number of ribosomes in the cell at the beginning of the run.		

4.4 PL_CONTROL PACKAGE

To facilitate the access to the above low-level interface of the configuration registers, we developed a dedicated Python package for operating the FPGA (PL – programmable logic). Inside this package, the PL_CONTROL class is defined. The constructor of this class only receives the BAR (base address) of the iterative model wrapper in the ARM address space.

Then, the class uses the MMIO class from the PYNQ package to access the above list of objects. When instantiating the class, the created object exports the following methods:

assert_model_reset() – resets the iterative model.

deassert_model_reset() – de-asserts the model reset and by that, letting it run until the configured stopping time.

assert/deassert_config_reset() – asserts/de-asserts the configuration reset to allow the model configuration before running it.

config_model_time (stopping time) – This function configures the stopping time of the simulation (receives the required value as an argument).

set_number_of_ribosomes (num_ribosomes) – configures the number of ribosomes in the cell to the received value.

config_mrna_codon (mrna_indx, codon_indx, codon_code) – this function configures the *codon_indx* codon in the *mrna_indx* mRNA with *codon_code* value.

read_protein_counter (mrna_indx) – this function returns the current generated proteins counter of the required mRNA index.

read_num_free_ribos() – this function returns the current number of free ribosomes in the model.

print_hardware_status() – prints basic information regarding the iterative model status: the *model_done* signal status, the current model time, the current number of free ribosomes and the number of generated proteins for each mRNA up to this point.

5 HDL DETAILS

5.1 MODEL TOP HIERARCHY

Before wrapping the model with the AXI wrapper (which will be described later), we have created a top module that contains all mRNA molecules and the global arbiter. This top module is usually generated via a dedicated python script that instantiates the mRNA molecules and configures the mRNA modules parameters. This python script also generates the ".mem" configuration files that contains the specific codon data of each mRNA molecule (for initialization). The following is the high-level module's hierarchy.

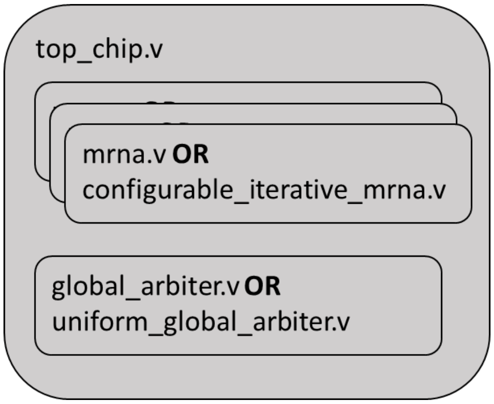

Figure S19 – HDL modules' hierarchy of the top chip module.

The python script that generates the module can choose whether to use the round robin arbiter (via *global_arbiter.v*) or to use the unform global arbiter (via *uniform_global_arbiter.v*). Also, the instantiated mRNA modules can be either the parallel mRNA modules (*mrna.v*) or the iterative mRNA modules (*configurable_iterative_mrna.v*). The interface between the mRNA molecules and the arbiter is kept the same for all modules' combination so the design can be changed smoothly, and various combinations of the arbiter type and the mRNA type can be examined.

Also, as the iterative mRNA module is the one finally used in the POC, it also supports the configuration features required for the POC. Those features can be similarly added to the parallel mRNA if needed.

The python script used to generate the top module also generates the simulation test bench and the required supplementary files needed.

5.2 MODEL TOP INTERFACE AND FURTHER DETAILS

The following is the list of parameters for the automatically generated top chip module.

Parameter	Description	Nominal values
**P_NUM_MRNAS**	This parameter dictates the amount of mRNA molecules instantiated in the top module.	Should be always a power of 2. Also, currently, the uniform arbiter supports up to 1024 endpoints, so this value must not exceed

		1024 without updating the uniform arbiter.
**P_GLOBAL_TIME_WIDTH**	The bit width of the local timer of each mRNA molecule.	Up to 32 bits (as that is the width of the AXI registers).
**P_FREE_RIBOS_COUNTER_WIDTH**	The width of the ribosomes counter.	Also should be up to 32 bits as that is the width of the AXI registers.
**P_GENERATED_PROTS_COUNTER_WIDTH**	The width of the local proteins counter for each mRNA molecule.	For the POC, 10 bits suffices. This value should also be kept bellow 32.

Next, here is the list of the interface signals of the module:

Signal name	Direction	Type	Description
**clk**	Input	Clock	That is the clock signal for the model. Currently, the maximal frequency allowed is up to 200MHz.
**rst_n**	Input	Reset	That is the reset signal for the model.
**conf_clk**	Input	Clock	That is the configuration clock – used to configure the mRNA memories, the stopping time and also the number of ribosomes in the cell. It is also used to sample busses that are later copied to the AXI registers.
**config_rst_n**	Input	Reset	Configuration reset signal. Resets the configuration registers and logic (like the stopping time register).
**memory_config_enable**	Input	Enable	When 1, the module configures the mRNA codon's list with the supplied values.
**config_mrna_indx**	Input	Bus	That is the address of the mRNA molecules that is about to be configured (its codons' ROM is about to be updated).
**config_codon_indx**	Input	Bus	The index of the codon in the pointed mRNA (via *config_mrna_indx*) that is going to updated in the codon's ROM.
**config_codon_data**	Input	Bus	The new value of the configured codon.
**stop_time_config_enable**	Input	Enable	When 1, this signal enables the update the model stopping time.
**stop_time**	Input	Bus	The required value of the model stopping time.
**num_ribosomes**	Input	Bus	The assigned number of ribosomes for the current model run.
**real_time_ms**	Output	Bus	The current model time in milliseconds (in real cell time).
**porteins_counters_flat**	Output	Bus	This is a flatten bus which contains the concatenated protein counters of all mRNA molecules. That is used by the wide multiplexer in the AXI wrapper (described later).

**model_done**	Output	Interrupt	This is the output interrupt that the model raises when reaching the configured stopping time.
**free_ribosomes_counter**	Output	Bus	The current value of free ribosomes in the cell.

Also, the module contains the *or_release_ribo_list* and *or_release_ribo_from_mrna_list* for debug purposes.

Moreover, apart from containing the instantiations of the mRNA molecules and global ribosome, this module is also responsible for sampling the stopping time and generating the *mrna_wr_en* signals for all mRNAs. This signal is calculated in the following generate block:

```
genvar g_indx;
generate
    for (g_indx=0; g_indx < P_NUM_MRNAS; g_indx=g_indx+1)
        assign mrna_conf_wr_en_list[g_indx] = memory_config_enable
                                            & (g_indx == config_mrna_indx);
endgenerate
```

Furthermore, the current time of the model is calculated in all mRNA molecules simultaneously. For the sake of generating the *model_done* interrupt signal, the top module uses the current time port of the first mRNA (arbitrarily). The synthesis process recognizes that the other counters are not in use and deletes their instantiation to reduce the utilization. That is done for the sake of simplicity in the routing and instantiation of the mRNA molecules.

Also, the top module also contains the generation of the hold signal which is shared between all mRNAs to keep the state machine synchronization. This signal is only used in the iterative model since the parallel model in synchronized in its nature.

At the beginning, the hold generation was simply done by the negation of the bitwise and of the *ready_for_next_iteration* signals from all mRNAs as follows:

```
wire [P_NUM_MRNAS - 1 : 0] done_iter_list;
wire hold;
assign hold = !(&done_iter_list);
```

Later, to ease the router’s task to meet the timing constraints, this wide and gate was replaced by a pipelined version of it for the case of 1024 mRNAs, as follows:

```
wire [1023 : 0] done_iter_list;
reg [1:0] and_done_iter_list;
wire hold;

always @(posedge clk) begin
    and_done_iter_list[0] <= &(done_iter_list[511:0]);
    and_done_iter_list[1] <= &(done_iter_list[1023:512]);
end
assign hold = !(&and_done_iter_list);
```

And finally, before the instantiation of the global arbiter, the module contains sample logic for sampling the free ribosomes counter at the exact moment in which the *model_done* signal goes high.

5.3 Round-robin global arbiter

The round-robin arbiter is the simplest version of the global arbiter. Apart from having the *P_NUM_MRNAS* parameter as before, it also has the following parameters:

Parameter	Description	Nominal values
**P_NUM_RIBOS**	The number of ribosomes in the cell. This value initializes the internal free ribosomes' pool counter.	This module was not used in the final POC of the system, so the value is hard coded by this parameter. Having this value configurable as in the uniform arbiter is easy and can be done in the same way.
**P_MAX_FREE_RIBOS**	Contains the maximal amount of release events that can occur between consecutive visits of the arbiter in the same mRNA molecule.	The main text includes an explanation on how this value was calculated.

The interface of the module consists of the *clk* and *rst_n* signals as before and the buses *mrna_request, mrns_release* and *mrna_grant* which are all of width *P_NUM_MRNAS* and contain the request and release signals of all mRNAs and the output grant signal for all mRNAs. Notice that later, this interface was changed for the uniform arbiter to avoid the demultiplexer for the *mrna_grant* bus.

The module contains a simple counter of the free ribosomes in the cell. The counter is initialized via the *P_NUM_RIBOS* parameter and is decreased upon granting a ribosome. This value is increased when the arbiter lands on an mRNA molecule that has release ribosomes from the last time the arbiter visited that mRNA.

That means that the global arbiter module should keep a local counter, for each mRNA molecule, that counts the number of ribosomal release events until the next time it visits the same mRNA. The width of that counter is the *P_MAX_FREED_RIBOS* parameter (its calculation is explained in the main text). This is done by the following generate block:

```
genvar g_indx;
generate
    for (g_indx=0; g_indx < P_NUM_MRNAS; g_indx=g_indx+1) begin
        always @(posedge clk) begin
            if (~rst_n)
                freed_counter[g_indx] <= {P_MAX_FREED_RIBOS{1'b0}};
            else
                freed_counter[g_indx] <= (current_mrna == g_indx)
                                        ? {P_MAX_FREED_RIBOS{1'b0}}
                                        : freed_counter[g_indx] +
mrna_release[g_indx];
        end
    end
endgenerate
```

In this code, the *freed_counter* of each mRNA molecule is cleared whenever the arbiter is currently visiting this mRNA.

Then, the new value of the free ribosome counter is calculated as follows:

```
always @(posedge clk) begin
    if (~rst_n)
        free_ribos_counter <= P_NUM_RIBOS;
    else
        free_ribos_counter <= free_ribos_counter
                              + freed_counter[current_mrna]
                              - (mrna_request[current_mrna] & available_ribos)
                              + mrna_release[current_mrna];
end
```

Which basically means that the new value of the *free_ribos_counter* is calculated by adding the current value of the local release counter of each mRNA to the current value, decreasing the counter by 1 if the current mRNA requests ribosomes (and there are ribosomes available) and increased by one if it happens to be a clock cycle in which a ribosome is released from the current mRNA.

5.4 UNIFORM GLOBAL ARBITER – IMPLEMENTATION DETAILS

The hierarchy of this module is as follows:

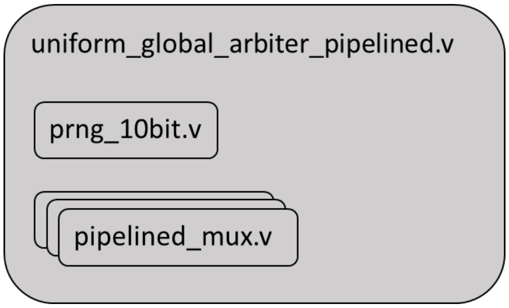

Figure S20 – HDL modules hierarchy of the pipelined uniform global arbiter module.

The module consists of one instantiation of the pseudo random number generator (*prng_10bit.v)* which generates random 10-bit numbers with uniform probability. As the module name suggests, this module is a pipelined version of the original module named *uniform_global_arbiter.v*. The pipelining was added to increase the clock frequency of this arbiter (as the timing reports revealed that the critical path was this arbiter for large amount of mRNA molecules).

In this module, 3 pipelined multiplexers are needed. The first is used to view the selected mRNA request signal, the second for the selected mRNA release signal and the third for the selected mRNA local *freed_counter*. Those signals also exist in the round robin arbiter and similar pipelining can be used to produce a pipelined version of the round robin arbiter.

5.4.1 Pipelined multiplexer

The pipelined multiplexer is built by two hierarchies of multiplexers as follows:

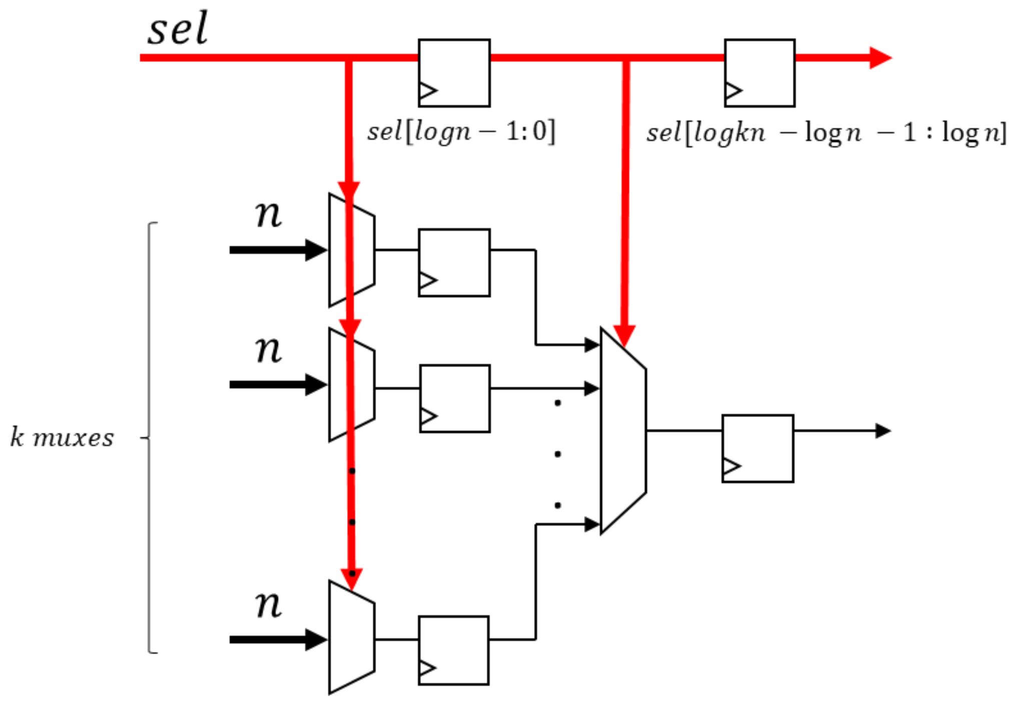

Figure S21 – The pipelined multiplexer modules' architecture. It consists of two levels of multiplexers with registers to sample the intermediate values. Also, the inputs are delayed for the output and for keeping the data and control signals synced to each other.

In this diagram, we see k multiplexers of width n. The output of those multiplexers is sampled and then inputted to a single mux of width k. The output of that mux is also sampled. This complex, generates a pipelined mux that can selected between $k * n$ inputs. The selector width is therefore $\log(k * n)$. The lower bits of the selector are routed to all the multiplexers of the input level. The input to each of those multiplexers is the subsequent section of n signals among the total $k * n$ signals. Then, the selector is delayed, and the upper bits of the selector are used to select among the outputs of the multiplexers of the first level. Notice that the selector is also delayed by another clock cycle, so the module's output consists of the selected value alongside its index.

This module has two important parameters: *P_MUX_WIDTH_LVL1*/2 – those parameters are the values of n and k.

5.4.2 PRNG

The module *prng_10bit.v* is based on a python script that generates the Verilog code with few modifications. The python script generates the generation matrices shown in the main text and uses them to generate the internal state registers x0 and x1. Those registers are then inputted into the output matrix (also generated by the python script). Here is an example code for the x0 state register:

```
always @(posedge clk) begin
    if(!rst_n)
        x1 <= state_init[2 * A_DIM - 1 : A_DIM];
    else begin
        x1[0] <= x1[0] ^ x1[1] ^ x1[3] ^ x1[4] ^ x1[12];
        x1[1] <= x1[3] ^ x1[4] ^ x1[6] ^ x1[10] ^ x1[11] ^ x1[12];
        x1[2] <= x1[9] ^ x1[10] ^ x1[11] ^ x1[12] ^ x1[15];
        x1[3] <= x1[0] ^ x1[4] ^ x1[7] ^ x1[8] ^ x1[11];
        x1[4] <= x1[0] ^ x1[3] ^ x1[4] ^ x1[9] ^ x1[11] ^ x1[14];
```

```
        x1[5] <= x1[0] ^ x1[1] ^ x1[2] ^ x1[4] ^ x1[6] ^ x1[14];
        x1[6] <= x1[3] ^ x1[4] ^ x1[5] ^ x1[7] ^ x1[9];
        x1[7] <= x1[5] ^ x1[7] ^ x1[11] ^ x1[12] ^ x1[13] ^ x1[15];
        x1[8] <= x1[0] ^ x1[10] ^ x1[14] ^ x1[15];
        x1[9] <= x1[0] ^ x1[1] ^ x1[5] ^ x1[7] ^ x1[15];
        x1[10] <= x1[2] ^ x1[8] ^ x1[9] ^ x1[12] ^ x1[13];
        x1[11] <= x1[8] ^ x1[9] ^ x1[11] ^ x1[14];
        x1[12] <= x1[3] ^ x1[4] ^ x1[9] ^ x1[10] ^ x1[13];
        x1[13] <= x1[2] ^ x1[3] ^ x1[6] ^ x1[13] ^ x1[15];
        x1[14] <= x1[2] ^ x1[4] ^ x1[9] ^ x1[10] ^ x1[11] ^ x1[15];
        x1[15] <= x1[7] ^ x1[9] ^ x1[10] ^ x1[13] ^ x1[15];
    end
end
```

In this code we can see that the state is initiated via an input port (*state_init*) and then, at each clock cycle, each bit of the state register is calculated by XOR-ing at most 6 bits of the current state. As mentioned in the main text, we XOR up to 6 bits to not exceed the maximal input number of each LUT in the ZCU104 FPGA. If we were to use more bits, then the synthesis would use more than one LUT to generate each state bit and that would impact the maximal clock frequency of the module and the overall utilization. By keeping this module compact as we did, it is also possible to use this module later for generating random numbers in the design for other features (as the arbitration over the tRNA pools and the random variables of the codon delays for example).

Also, notice that the x0 state register only advances after 2^{16} cycles of the x0 state register to increase the period of the output stream.

Then, the output of the module is calculated via mixing the state bits with the output matrix. Here is an example for the generated code:

```
always @(posedge clk) begin
    local_random_number[0] <= x0[4] ^ x0[10] ^ x0[14] ^ x1[1] ^ x1[5] ^ x1[13];
    local_random_number[1] <= x0[8] ^ x0[13] ^ x0[14] ^ x1[1] ^ x1[11] ^ x1[12];
    local_random_number[2] <= x0[2] ^ x0[3] ^ x0[13] ^ x1[0] ^ x1[3] ^ x1[11];
    local_random_number[3] <= x0[5] ^ x0[8] ^ x0[9] ^ x1[6] ^ x1[8] ^ x1[15];
    local_random_number[4] <= x0[6] ^ x0[7] ^ x0[11] ^ x1[4] ^ x1[9] ^ x1[14];
    local_random_number[5] <= x0[1] ^ x0[2] ^ x0[8] ^ x1[1] ^ x1[2] ^ x1[11];
    local_random_number[6] <= x0[9] ^ x0[10] ^ x0[12] ^ x1[6] ^ x1[8] ^ x1[10];
    local_random_number[7] <= x0[0] ^ x0[4] ^ x0[5] ^ x1[0] ^ x1[2] ^ x1[14];
    local_random_number[8] <= x0[1] ^ x0[10] ^ x0[15] ^ x1[5] ^ x1[7] ^ x1[14];
    local_random_number[9] <= x0[5] ^ x0[0] ^ x0[12] ^ x1[3] ^ x1[9] ^ x1[1];
end

assign random_number = local_random_number[P_OUTPUT_WIDTH - 1 : 0];
```

Here we can see that we also do not exceed 6 inputs to each XOR for the same reasons as before. Also, the output of the module is the lower *P_OUTPUT_WIDTH* (one of the modules' parameters) bits of the random number. It is important to notice that the python code generates this module so if the width of the output matrix is 10 bits, as in this example, the output width of the module is up to 10 bits.

5.4.2.1 PRNG – alternative hash-based implementation

We have also considered using hash functions to generate the uniform random numbers for the global arbiter as follows:

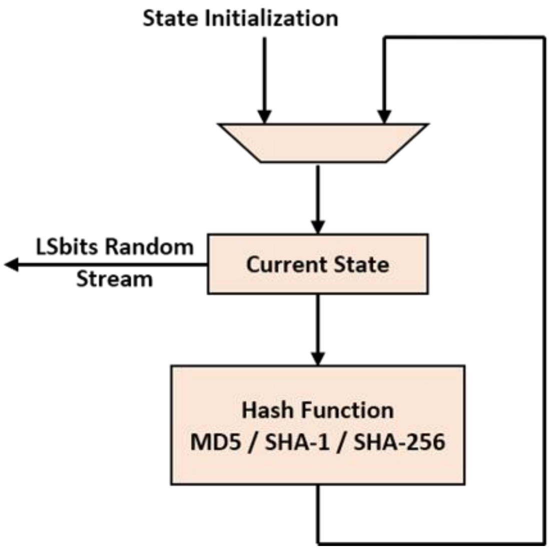

Figure S22 – Using hash functions to generate random numbers. In the figure we can see that the state is initialized via an arbitrary value and then digested by the hash function repeatedly. The random stream consists of the least significant bits of the current state.

As shown in the figure, we examined the common MD5, SHA-1 and SHA-256 hash functions. We have compared this method to the matrix method, that was shown in the main text, considering the following features:

1. Logic consumption – how much resources does the hardware implementation of the module requires.
2. Random quality – we wish that the output distribution will be as close to uniform as possible (to have the *p-value* as close to 1 as possible).

To estimate the utilization of the hash functions, we used the commercial reports of Helion hashing IPs[6]. Our FPGA chip is part of the Ultrascale+ family of FPGAs, and according to their reports, the logic consumption is 169 CLBs (configurable-logic-blocks). Each CLB consists of 8 LUTs, so each hash function is going to use 1352 LUTs.

The utilization in our PRNG consists of the following:

1. Calculating the state registers using the matrices *A0, A1* – as shown, each state register consists of 16 bits and the calculation of each bit is designed to consume only one LUT. Therefore, the calculation of the state registers consumes 32 LUTs.
2. Calculating the output value – the output of the module consists of 10 bits that are generated by XOR combinations of up to 6 bits from the state registers, so as for the state registers, each output bit requires a single LUT. Therefore, the output calculation consumes 10 LUTs.
3. Generating the iteration counter – we count to 2^{16} before we advance the second state register. From Xilinx counter IP core resource utilization report[7], we can see that an 18 bits counter consumes 19 LUTs. So, as we are having a 16 bits counter, we consume up to this value.

[6] https://www.heliontech.com/fast_hash.htm
[7] https://www.xilinx.com/html_docs/ip_docs/pru_files/c-counter-binary.html

So, as the utilization analysis of our PRNG shows, our PRNG consumes up to 61 LUTs. That is expected because the PRNG shown here was design specially to utilize the LUT properties of our FPGA to construct a fast and low-weight module. This low resource utilization is also necessary for future features that will also require random variables (tRNA pool arbitration and translation delays as random variables).

Next, we calculated the p-value of the output streams of each of the techniques. We used a stream of 100 million values to get an accurate estimate for the p-value. Here are the results:

PRNG type	p-value (100M stream)	LUT consumption
**Matrix multiplication**	0.991632965695079	up to 61 LUTs
**Hash – MD5**	0.8469259137833651	1352 LUTs
**Hash – SHA-1**	0.7143014578650664	1352 LUTs
**Hash – SHA-256**	0.6359696921506162	1352 LUTs
**Python random** (for reference)	0.796723329718767	-

From this table, we can see that the presented PRNG that was finally used in our work is very light weighted in terms of FPGA resource utilization in comparison to the hashing technique. Also, the PRNG developed here yields a random stream with the best *p-value*.

To improve the random quality of the hashing techniques, one might consider adding an output matrix as follows:

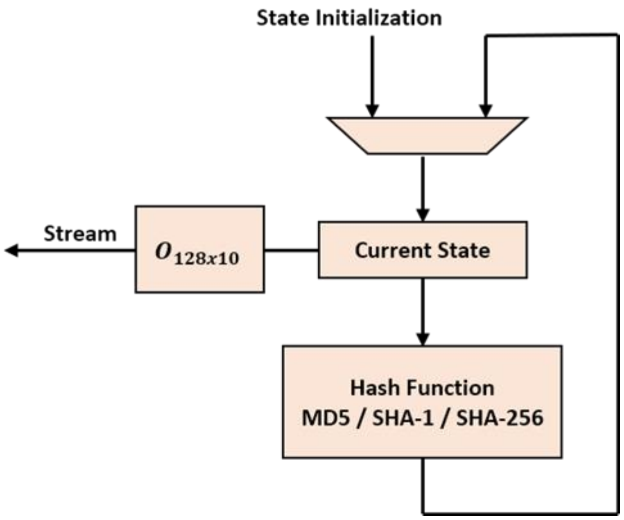

Figure S23 – random number generator using hash table and an output matrix. This is the same figure as in the previously presented hash PRNG but with an output matrix that might improve the output quality.

The output matrix O_{128x10} takes the hash's state (for MD5 it is 128) and generates the 10 output bits combining several state bits (via XOR) to generate each output bit (similarly as shown in the matrix PRNG). Although that might improve the output quality, it will only increase the LUTs utilization. In the presented PRNG we have already got a very high-quality random stream for very low resources utilization.

5.5 UNIFORM ARBITER – INTERFACE AND PARAMETERS

The interface of the pipelined uniform arbiter module is more complex than the interface of the round robin arbiter. The reason is that this module contains a pipelined mux that should be configured, and the output of the module no longer consist of a list of separate grant signals, as in the round robin arbiter, to avoid the large output demultiplexer.

The parameters of this module include *P_NUM_MRNAS, P_MAX_FREE_RIBOS* with the same functionality as in the round robin arbiter. Those are the added parameters:

Parameter	Description	Nominal values
**P_FREE_RIBOS_COUNTER_WIDTH**	The width of the free ribosomes' counter in the cell. In the round robin arbiter, the number of the ribosomes was a static parameter. Here, the value is an input port, so we must limit its width.	Up to 32 bits (the value of the counter propagates from the AXI registers).
**P_MUX_WIDTH_LVL1/2**	As explained in the pipelined multiplexer module – those parameters define the width of the first and second level multiplexers.	The sum of those values must be exactly $\log(\mathrm{P\_NUM\_MRNAS})$.
**P_MRNA_INDX_WIDTH**	The width of the outputted mRNA index. The output of the module contains the index of the selected mRNA module.	Must be log(P_NUM_MRNAS).

The interface of this module is like the interface of the round robin arbiter with the following changes. First, we do not have the *mrna_grant* bus. Instead, we output the *granted_mrna_indx* value which is used by the top module (as explained) to generate the local grant signal for each mRNA molecule. This value also has a valid signal (*grant_valid*) that signals if this bus value is valid (in cases that the arbiter visits an mRNA molecule but does not grant a ribosome).

The *grant_valid* and *granted_mrna_indx* are generated by the following always block:

```
always @(posedge clk) begin
    if (!rst_n) begin
        granted_mrna_indx <= {P_MRNA_INDX_WIDTH{1'b0}};
        grant_valid <= 1'b0;
    end else begin
        granted_mrna_indx <= selected_mrna;
        grant_valid <= selected_mrna_request & available_ribos;
    end
end
```

In this code, we can see that the output index of the selected mRNA is the value that is generated by the PRNG (*selected_mrna*) after being delayed by the pipelined multiplexer (to keep the module in sync). The *grant_valid* signal is 1 only if the module currently has free ribosomes (*available_ribos*) and the selected mRNA requests a ribosome (*selected_mrna_request*).

Also, the interface includes the *free_ribos_counter* bus which contains the number of currently free ribosomes in the cell.

5.6 PARALLEL MRNA MODULE – INTERNAL MODULES

Here we provide further details regarding the parallel mRNA module. The hierarchy of this module is as follows:

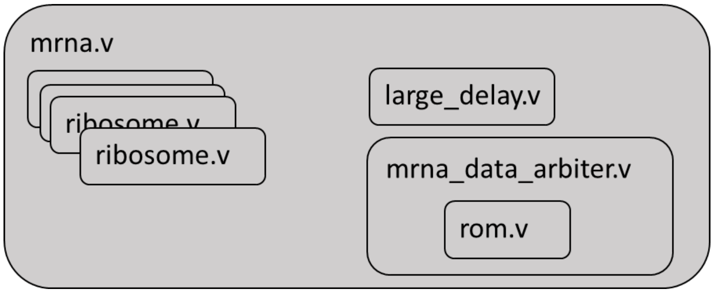

Figure S24 – HDL modules' hierarchy of the parallel mRNA module.

As shown in the main text, this module consists of a concatenated structure of hardware ribosomes (*ribosome.v*). This module also contains the codon's data inside a ROM (*rom.v*) that is controlled via an arbiter that servers all ribosomes (*mrna_data_arbiter.v*). Let us begin by first explaining the structure of each module and then we proceed with the high-level module interface and parameters.

5.6.1 Hardware ribosome module

The hardware ribosome essentially consists of the state machine which is presented in the main text. This module receives the following parameters:

Parameter	Description	Nominal values
**P_MRNA_LENGTH**	The length of the mRNA molecule (in codons).	Positive integer.
**P_CODON_INDX_WIDTH**	The width of the index to the codons in the specific mRNA. This value depends on the length of the mRNA and therefore parametric.	Must be $\lceil \log(P\_MRNA\_LENGTH) \rceil$
**P_CODON_DELAY_WIDTH**	The width of the translation delay timer of the codons.	This value should be calculated according to the maximal delay of the slowest codon.
**P_RIBO_MIN_DISTANCE**	The minimal distance (in codons) that should be kept between two consecutive ribosomes).	Positive integer.
**P_RIBO_ALLOC_DELAY**	The allocation time for the specific mRNA. As shown in the main text, this value was used when the allocation delay was part of the ribosomes' state machine.	Positive integer.
**P_RIBO_INDX**	The index of the current ribosome in the hardware ribosomal chain. This value is used to check if the current served	From 0 to the number of ribosomes in the local mRNA (excluding).

	ribosome by the data arbiter is the local ribosome.	
**P_MAX_ACTIVE_RIBOS**	Number of hardware ribosomes in the current mRNA.	Positive integer.

Those parameters are automatically assigned to the ribosome module via a large generate block inside the *mrna.v* module. That basically means that those parameters are either propagated from the *mrna.v* parameters or are generated using the generate variable. As mentioned in the top module section, the parameters for the mRNA modules are generated via the python script with accordance to the various parameters of each mRNA in E.coli.

Next, here are the non-trivial ports for the ribosome module:

**Signal name**	**Direction**	**Type**	**Description**
**activate**	Input	Control	This signal is generated by the mRNA module's state machine. When the mRNA receives a new ribosome from the global arbiter, the mRNA state machine activates the next IDLE hardware ribosome. This signal enables the ribosome's state machine.
**next_ribo_indx**	Input	Bus	This is the connection to the codon index of the consecutive ribosome. This signal is used to keep the minimal distance between the ribosomes.
**codon_indx**	Output	Bus	The index of the current codon. This bus is connected to the data arbiter of the mRNA used to request the translation delay of the current codon from the ROM.<br>This bus is also connected to the next hardware ribosome's *next_ribo_indx* bus.
**req_codon_delay**	Output	Control	When 1, the ribosome waits for the arbiter to send the delay of the current codon from the ROM.
**codon_trans_delay**	Input	Bus	The value of the translation delay from the ROM arbiter.
**current_serverd_ribo**	Input	Bus	The index of the current served ribosome by the mRNA's ROM arbiter.
**codon_trans_delay_valid**	Input	Control	This is the valid signal for the *current_served_*ribo and *codon_trans_*delay busses.
**done_generating_protein**	Output	Control	When 1, the ribosome finished generating the protein of the current mRNA. After raising this signal, the ribosome goes to IDLE state until re-activated.

Apart from the state machine that is brought in the main text, it is important to understand the following aspects of the module. First, the *done_generating_protein* signal can be 1 only for one single hardware ribosome (for each mRNA). That is because the hardware ribosomes are concatenated, and it is not possible for a ribosome to generate a protein if it is not the current first active ribosome.

Also, the *next_ribo_indx* is wired to the concatenated ribosome. This signal is important for keeping the required distance from the consecutive ribosome. This bus is used to generate the *clear_to_move* signal that signals the ribosome's state machine that the ribosome can advance to the next codon. This signal is generated as follows:

```
wire clear_to_move;
assign clear_to_move = (next_ribo_indx <= codon_index) ? 1'b1
                        : (next_ribo_indx - codon_index > P_RIBO_MIN_DISTANCE);
```

In this code, we first check if we are the first ribosome. The check is done by comparing the current index to the next ribosome index. If the current index is bigger than the next ribosomes' index, it means that that is the first active ribosome and there are no active ribosomes ahead of it. In that case, the *clear_to_move* signal goes high. Otherwise, the ribosome can advance the current codon index only if the distance between this ribosome to the next is bigger than *P_RIBO_MIN_DISTANCE* parameter (see the parameters table).

Also, to keep the ribosome as minimal as possible, it is important to make sure that the coding of the states in the state machine allows compact usage of the LUTs. For example, let us view the following code in the ribosome module:

```
always @(posedge clk) begin
    if (~rst_n)
        codon_delay_timer <= {P_CODON_DELAY_WIDTH{1'b0}};
    else
        codon_delay_timer <= (current_state == S_CODON_TIMER_INIT)
        ? codon_trans_delay : codon_delay_timer - 1'b1;
end
```

Here we can see that the delay timer of the current codon is handled. We can see that if the ribosome's state machine is in the initialization state, the timer is initialized to the value that eventually comes from the ROM's arbiter. Otherwise, the timer decrements by 1. The comparison of the state register *current_state* to the required state (*S_CODON_TIME_INIT*) might consume more resources if the states are not coded properly. In this case for instance, if we use one-hot coding for the states, the comparison *current_state == S_CODON_TIMER_INIT* will be equivalent to just a single bit in the state register. Fortunately, Vivado synthesis tools automatically infers that the *current_state* register is part of a state machine and re-codes the states. It is important to view the synthesis reports to see that the new codes make sense.

5.6.2 Large delay module

This module is used for delaying the *done_generating_protein* signal for modeling the diffusion property of the ribosomes. The diffusion property basically means that it takes time for a ribosome to be available again by the cell after it is released from the mRNA molecule. The diffusion delay in clock cycles is chosen to be the value that causes some percentage of ribosomes to be in diffusion state in steady state. For E.coli, nominally there are 30% of the ribosomes in diffusion in the steady state. Therefore, the delay value is calculated as follows:

$$diffusion\ delay = \frac{\sum_{i=0}^{M-1}\left(A_i + \sum_{j=0}^{L_i-1} c_j^i\right)}{M}\left(\frac{D}{1-D}\right)$$

M is the number of mRNA molecules, A_i is the allocation delay of the i-th mRNA, L_i is the length of the i-th mRNA, c_j^i is the translation delay of the j-th codon of the i-th mRNA and D is the diffusion factor (0.3 for 30%). This formula basically calculates the average time it takes for an mRNA molecule to be translated (ignoring the traffic jams) and uses it to calculate the time a ribosome should wait for receiving the required diffusion percentage. This formula is an approximation (because it ignores the ribosomes' traffic jams) but it yields the required results in practice.

Using this formula, in E.coli, we get that the diffusion time is between 20,000 milliseconds and 40,000 milliseconds. This value is quite large. The most straight forward implementation of a delaying module is a simple shift register. In this case, a shift register should be of length of 30,000 registers on average. This shift register needs to be duplicated for the number of mRNA molecules in the design. So, for 1024 mRNAs, if we use a simple shift register, it will consume $30{,}000 * 1024 = \mathbf{30.72}\ \boldsymbol{M}$ **registers**. In our chip, we only have 460,800 flip-flops. Since we cannot afford a simple shift register, we then considered using the SLICEM slices in the Zynq FPGA. Those specific slices, allow using the LUT's internal memory as a shift register. Here is a block diagram for those LUTs (taken from Xilinx xapp465 datasheet):

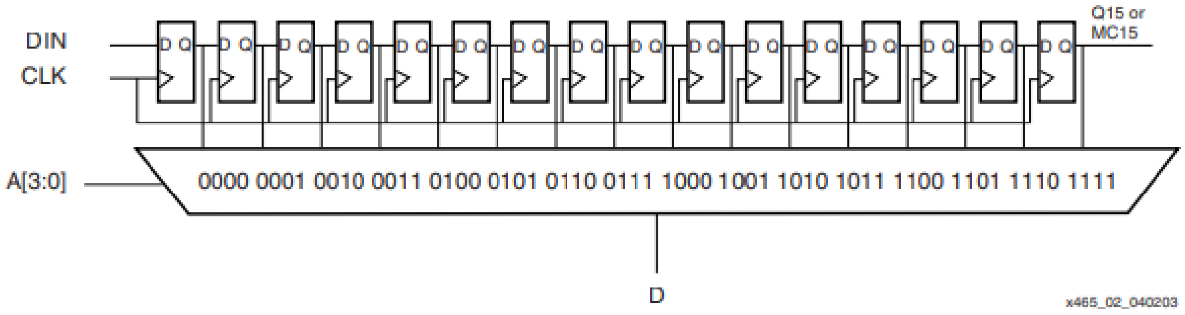

Figure S25 – An example of a 4 inputs LUT which can also be used as a shift register. This image is taken from Xilinx* xapp465 *datasheet.

In our FPGA, we have 6 input LUTs. Therefore, in each LUT we can store up to $2^6 = 64$ bits of our shift register. In ZCU104 we have 101,760 LUTs that can be used as shift registers. Therefore, we can store up to: $101{,}760 * 64 = 6{,}512{,}640$ bits of shift registers. Therefore, also when using the SLICEM LUTs, we do not have enough resources for implementing the diffusion delay as shift-registers.

Therefore, we developed the *large_delay.v* module that utilizes the specific features of delaying the release signal of a ribosome from an mRNA molecule. The module has two parameters:

P_NUM_FAST_CLK_CYCLES – this parameter is the value of the required diffusion delay in clock cycles. As mentioned, for E.Coli it should be around 30,000.

P_NUM_CLK_CYCLES_PER_ITER – this parameter is used to define an internal counter that generates a slow enable signal.

Then, the module delays the fast input signal by approximately *P_NUM_FAST_CLK_CYCLES*. It is done by an internal shift register of size *P_NUM_FAST_CLK_CYCLES / P_NUM_CLK_CYCLES_PER_ITER* that advances each *P_NUM_CLK_CYCLES_PER_ITER*. There are certain points that should be mentioned here. The first, is that the value of the parameters should be chosen as such that no release event of a ribosome can occur in the *P_NUM_CLK_CYCLES_PER_ITER* consecutive clock cycles after the previous ribosome

release. That utilizes the fact that ribosomes are released at a rate that is bounded by the time it take to translate the last 9 codons (the minimal distance in E.coli).

The second point is that the *fast_signal* input to the module (the release signal) is asynchronous to the local iteration counter and is kept until the next iteration begins. That causes a slight variation in the diffusion delay (typically around half *P_NUM_CLK_CYCLES_PER_ITER*).

5.6.3 mRNA data arbtier

As mentioned in the main text, instead of copying the codons' delay for each ribosome, a single ROM is kept and arbitered using a simple round robin arbiter that serves all hardware ribosomes. That is implemented in the *mrna_data_arbiter* module.

This module contains the actual data of the mRNA's codons. As shown in the previous sections, we found that it is more memory efficient to keep the codon's data in two concatenated memories instead of a large, big one. For the parallel model, the memory was not the bottleneck for the utilization, so we kept a single large table from the codon index to the codon delay.

The table is stored in a ROM memory (in *rom.v*) module. The ROM is initialized using a ".mem" file that is generated by the Python script that also instantiate the mRNA modules as the path to that configuration file is a parameter of the mRNA module and eventually propagates to the ROM module.

Inside the ROM module we have the following code with important directives to the synthesis tool:

```
 (* rom_style = "block" *) reg  [DATA_WIDTH-1:0] mem [0:MEM_SIZE];
initial begin
    $readmemb(INIT_FILE, mem, 0, ACTUAL_MEM_SIZE-1);
end
```

In this code we first see the *rom_style* directive that direct the synthesis tool to implement the ROM as a BRAM. That is important because in the parallel case the memory is not the bottleneck. If we emit this directive, the synthesis might implement the ROM as distributed RAM and that will cost LUTs which are the bottleneck of the parallel design.

Also, in this code we can see the *$readmemb* directive that receives the *INIT_FILE* path (the .mem file). This directive directs the synthesis to initialize the BRAM in hardware with the contents of the given *INIT_FILE*. The format of the file is simply a list of binary encoded values of the bits inside the ROM. If one wished to save more disk files for the storage of those memory files, it is possible to use the *$readmemh* directive that receives files with data that is encoded in hexadecimal.

5.7 PARALLEL MRNA MODULE – PARAMETERS AND INTERFACE

As mentioned, the mRNA modules are parametric and the python script that instantiate them in the top module is responsible for setting the parameters. The parameters: *P_CODON_INDX_WIDTH, P_CODON_DELAY_WIDTH, P_RIBO_MIN_DISTANCE, P_MAX_ACTIVE_RIBOS, P_RIBO_ALLOC_DELAY, P_MRNA_LENGTH* are the same as presented in the ribosome module. The parameters *P_DEFUSION_TIME* and *P_DEFUSION_ITERATION_TIME* are the parameters for the *large_delay* module presented earlier. *P_MRNA_INIT_FILE* is the path of the ".mem" file used for initializing the internal ROM.

The interface of the module is very simple and is the same as shown in the high-level block diagram in the main text. Apart from the trivial signals (clocks and reset), the interface consists of the following:

Signal name	Direction	Type	Description
**req_ribo**	Output	Control	When 1, the mRNA is free to receive a new ribosome from the pool.
**release_ribo_to_pool**	Output	Control	When 1, the mRNA releases the ribosome to the global pool **after** diffusion.
**release_ribo_from_mrna**	Output	Control	When 1, a new protein is generated but the ribosome is first entering the diffusion state. This signal is delayed via the *large_delay* module to produce the *release_ribo_to_pool* output.
**ribo_granted**	Input	Control	When 1, the global arbiter grants a new ribosome to the mRNA molecule.

Notice that the *ribo_granted* signal is replaced with the mRNA index, that is currently served, in the final POC with the iterative mRNA. This simple adjustment can be applied here if necessary.

5.8 ITERATIVE mRNA MODULE

This module is used for the final proof-of-concept. The hierarchy of the module is as follows:

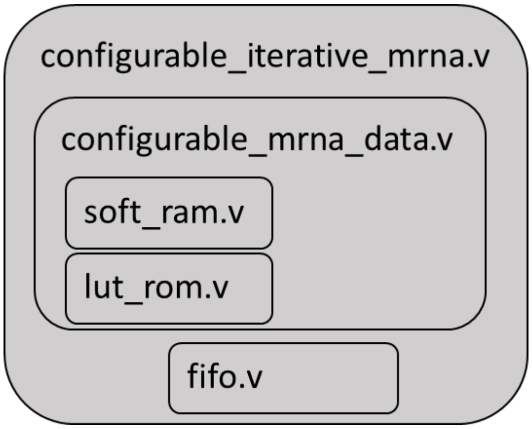

Figure S26 – HDL modules' hierarchy of the configurable iterative mRNA module.

The FIFO, as shown in the main text, is responsible for keeping the state of the ribosomes. The *configurable_mrna_data* module contains the codons' data and as the name suggests, this module also support reconfiguration of the codons' table. This module also contains two state machines as shown in the main text. The FIFO implementation is quite trivial so we proceed by describing the *configurable_mrna_data* module.

5.8.1 Iterative mRNA data module

This module is responsible for keeping and reconfiguring the mRNA's codon data. In the parallel model, the codons' data was stored inside the data arbiter module. Here, there is only one consumer for the codons' data – the mRNA state machine. Therefore, the arbiter is not required here.

As opposed to the parallel model, in which the bottleneck was the logic utilization, the bottleneck in the iterative model is the memory utilization. Therefore, here it is necessary to apply the two-table method that was described in full details in previous chapters.

The mapping between the codons' code to the codon data is stored in *lut_rom.v*. This is the exact same module as the *rom.v* module, that was part of the parallel models' data arbiter, apart from a small change:

```
  (* rom_style = "distributed" *) reg  [DATA_WIDTH-1:0] mem [0:MEM_SIZE];
```

The change is in the synthesis directive – instead of using the "block" directive, we use the "distributed". That directs the synthesis to favor the implementation of that memory using LUTs instead of BRAMs. As shown earlier, the synthesis indeed implements this memory using 16 LUTs as desired.

Also, notice that the initialization of this memory should be generated. As before, the *$readmemb* directive is used with the codons' delay values.

Next, the mapping between the codon index to the codons' code is stored in the *soft_ram.v* module. This module is like the ROM module. Its interface also includes the write logic required for updating the values. Also, the memory register declaration is as follows:

```
reg  [DATA_WIDTH-1:0] mem [0:MEM_SIZE];
```

Here we see that no specific directive was given to the synthesis. We found that this degree of freedom makes a difference and allows fitting more mRNAs in the design as the synthesis can implement small mRNAs using distributed memory and large mRNAs using BRAMs.

Also, this memory begins with an empty cell (initialized to 0). That is done to have the state machine treat the allocation delay as all other codons. The reason why we did not simply put the allocation delay as the first value in that memory is because the allocation delay is typically large and therefore that memory would have been unnecessarily wide.

5.9 ITERATIVE MRNA MODULE – INTERFACE AND PARAMETERS

Perhaps that is the module with the most complicated interface in our work. The complexity is derived from using this module as part of the proof-of-concept which required high visibility to internal signals and high configurability.

We begin by describing the modules' parameters. The parameters *P_CODON_INDX_WIDTH, P_CODON_DELAY_WIDTH, P_RIBO_MIN_DISTANCE, P_MAX_ACTIVE_RIBOS, P_RIBO_ALLOC_DELAY, P_MRNA_LENGTH, P_DEFUSION_TIME* and *P_DEFUSION_ITERATION_TIME* are the same as before. Those are the new parameters introduced by this module:

Parameter	Description	Nominal values
**P_MRNA_INDX**	The index of the current mRNA module. This is used when the arbiter outputs the index of the mRNA that is currently served.	Up to the number of mRNA molecules in the design.

**P_MRNA_INDX_WIDTH**	The width in bits of the mRNA index param.	Must be $\lceil \log(NUM\ MRNAS) \rceil$
**P_CODON_WIDTH**	The width of the codons' code.	6 bits.
**P_DELAY_WIDTH**	The maximal width of the ribosomes' timer that is used for the allocation delay and for the codons' delay.	Positive integer.
**P_ALLOC_DELAY_WIDTH**	The width of the allocation delay.	Positive integer.
**P_RIBO_FIFO_ADDR_WIDTH**	The address width of the FIFO used to store the ribosomes' state.	Ceil value of the log of the maximal amount of simultaneously active ribosomes on the current mRNA.
**P_LOCAL_TIME_WIDTH**	The width of the local time counter.	Positive integer (up to 32 bits to be read by the AXI registers).
**P_CODONS_MEM_FILE**	Path to the .mem file used to initialize the *lut_rom* module with the codons delay values.	File path.
**P_CODONS_DELAY_FILE**	Path to the .mem file used to initialize the *soft_rom* module with the specific mRNA's codons' list.	File path.
**P_GENERATED_PROTS_CNT_WIDTH**	The width of the local protein counter.	Up to 32 bits (eventually read by the AXI registers).

Next, the interface of the module consists of the following signals:

**Signal name**	**Direction**	**Type**	**Description**
**model_clk, rst_n**	Input	Clock, Reset	The clock and reset signals for the internal model state machine and internal logic.
**conf_clk, memory_rst_n**	Input	Clock, Reset	The clock and reset signal for the configuration of the module and the communication with the AXI interface (eventually).
**memory_wr_en**	Input	Control	Derived by the AXI registers. That is the write enable signal for the *soft_ram* module inside the module. This signal is generated by the top module for each mRNA separately according to the mRNA index that is set by the AXI registers.
**memory_wr_addr, memory_wr_data**	Input	Bus	Those are the write address and the write data for the internal codons' memory in the *soft_ram* module. Also derived by the AXI logic.
**req_ribo, release_ribo_to_pool, release_ribo_from_mrna**	Output	Control	The same as in the parallel mRNA module – those signals are used for signaling the global arbiter.

**grant_mrna_indx, grand_valid**	Input	Bus, Control	Those signals are derived by the global arbiter and are used to provide the index of the mRNA that received a ribosome (if *grant_valid* is high).
**ready_for_next_iteration**	Output	Control	This signal goes high whenever the internal state machine of the module finishes iterating over all active ribosome (or when there are no active ribosomes). This signal is used to synchronize all mRNA molecules in the chip via the *hold* signal that is generated in the top module.
**hold**	Input	Control	When this signal is High, the mRNA cannot proceed to the next iteration. As explained, this signal is responsible for synchronizing the mRNA molecules.
**local_time**	Output	Bus	The local time of the mRNA module in milliseconds in real cell time.
**proteins_counter**	Output	Bus	The proteins counter of the current mRNA.
**stop_counting**	Input	Control	When the model reaches the end time, the top module derives 1 to this signal to freeze the proteins counter.

The *grant_mrna_indx* and *grant_valid* signals replace the single input *ribo_granted* signal that was in the parallel mRNA to avoid a wide multiplexer. The mRNA modules derive the *ribo_granted* signal internally as follows:

```
assign ribo_granted = grant_valid & (granted_mrna_indx == P_MRNA_INDX);
```

Also, as can be noticed from the interface, the mRNA module is not aware of the stopping time directly, for simplicity. The only thing that is important when reaching the stopping time is to freeze the proteins counter and that is achieved using the *stop_counting*. The reset of the module can keep running until the next reset sequence initiated by the CPU using the AXI registers.

5.10 AXI WRAPPER MODULES

Finally, when instantiating the top module in the FPGA it should be wrapped with AXI interface to allow the connectivity to the ARM CPU core as shown above. The following is the hierarchy of the AXI wrapper:

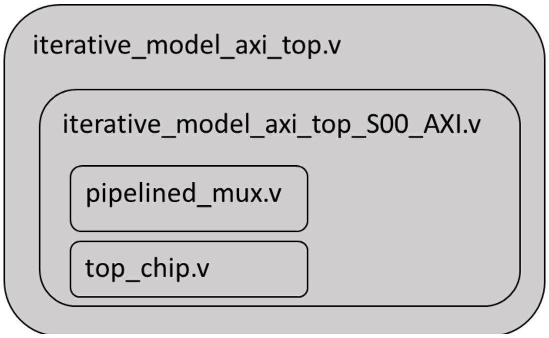

Figure S27 – AXI Wrapper HDL modules' hierarchy.

The module *iterative_model_axi_top.v* and *iterative_model_axi_top_S00_AXI.v* are coded based on a templated that is generated by the Vivado toolchain upon creating a new IP core with AXI Slave interface. Most of the work is done in the *iterative_model_axi_top_S00_AXI.v* module. The *iterative_model_axi_top.v* module contains an instantiation of the *S00_AXI* module and used for the AXI interface and parameter propagation to the IP top.

The AXI wrapper contains a pipelined mux for accessing the protein counters of the top chip module. As shown in the top chip interface, the protein counters are outputted to a flattened bus so they can be inputted to this pipelined multiplexer. This is used to allow better timing convergence.

Then, the top chip module is instantiated inside the wrapper. The wrapper template is automatically generated with the management logic for the AXI registers. Those registers are named *slv_reg0..15* as we generated the wrapper for only 16 registers. The configuration signals for the top module are assigned from the AXI registers as follows:

```
assign model_rst_n = slv_reg0[0];
assign model_config_rst_n = slv_reg0[1];
assign model_memory_config_enable = slv_reg0[2];
assign stop_time_config_enable = slv_reg0[3];
assign clear_interrupt = slv_reg0[4];

assign config_mrna_indx = slv_reg1[25:16];
assign config_codon_indx = slv_reg1[15:0];
assign config_codon_data = slv_reg2[5:0];

assign stop_time = slv_reg3;

assign mrna_addr_for_proteins_readback = slv_reg4[9:0];

assign num_ribosomes = slv_reg10;
```

Here we see that the relevant bits of the configuration registers are used to generate the input to the top chip. Those configuration registers are described in full details in previous chapters.

Also, we have also added to the top AXI wrapper the *model_done* interrupt signal to be connected to the interrupt pins of the ARM processor. The generation of this signal is done as follows:

```
assign model_done_introut = model_done & !clear_interrupt;
```

The *model_done* signal is generated from the top chip module as explained before, and the *clear_interrupt* is derived from the AXI registers.

5.11 Design adjustments for better implementation convergence

When instantiating the top chip module inside the AXI wrapper and trying to fit the entire system inside the chip we came across some extra complexities. Initially, the design was unrouteable, meaning that the router could not route all the nets that are connecting the different logic blocks. The reason for that is that some of the logic blocks where highly congested. A congested block is a logic block that has many signals that should be routed from it to other blocks. Using the tools, we got the following congestion heat map:

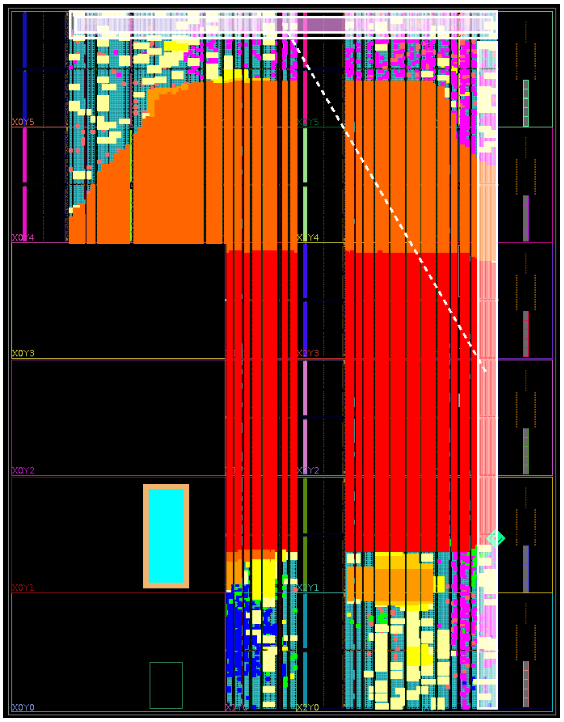

Figure S28 – This is also generated using the Vivado tool chain with accordance to our HDL code. Here we can see that we have the middle cells which high highly congested and are marked with red. Then there are the less congested cells denoted with orange and yellow. As shown in the highlighted rectangles along the upper and right edges – this congestion map is received after mapping the global arbiter to the corners.

In this heat map, we can see that the blocks in the middle are most congested. To spread the design even further, we used the floor planning feature and mapped the global arbiter to the upper and right edges of the chip (also shown in this figure). Although that improved the congestion, it was not enough to have the design routable. Here is the mapping of different logic modules to cell in the FPGA:

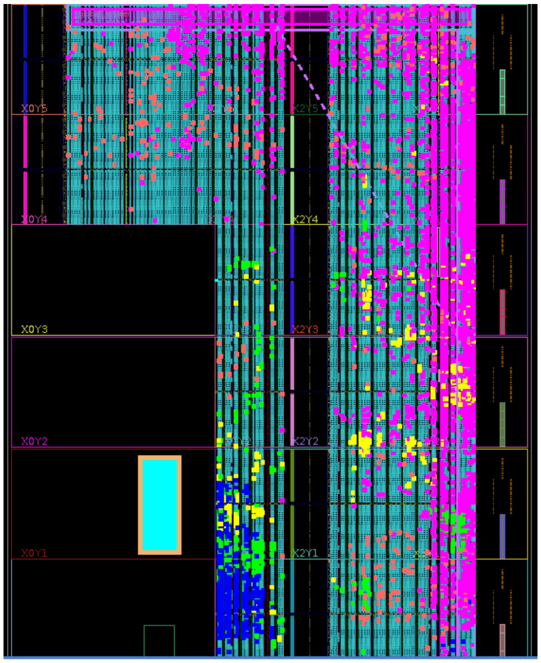

Figure S29 – This map was also generated using Vivado. Here, we view the congested design after the design placement stage. We colored some of the modules to see to where they were mapped. In pink – the global arbiter, in blue – AXI interconnect blocks, light-blue – the ARM cores and the Zynq PS (the large rectangle on the bottom-left), yellow – the first mRNAs (low indices), green – the last mRNAs (high indices), light-red – the proteins counter multiplexer in the AXI module.

By tracking the location to which the modules where mapped, we were able to see that the placement makes sense and no other placement adjustments should be made – the AXI logic is close to the Zynq PS, the mRNAs are located near the global arbiter and the proteins counter is spread across the chip.

The next thing that we examined to relax the congestion is the nets fanout. Here is the list of the high-fanout nets generated by Vivado:

iterative_model_v2_0_i/iterative_model_axi_0/inst/iterative_model_axi_top_v1_0_S00_AXI_inst/slv_reg0_reg[0]_rep__26_n_0_BUFG	88908	BUFGCE
iterative_model_v2_0_i/iterative_model_axi_0/inst/iterative_model_axi_top_v1_0_S00_AXI_inst/slv_reg1_reg_n_0_[0]_repN_6	6721	FDRE
iterative_model_v2_0_i/iterative_model_axi_0/inst/iterative_model_axi_top_v1_0_S00_AXI_inst/slv_reg1_reg_n_0_[1]_repN_6	6721	FDRE
iterative_model_v2_0_i/iterative_model_axi_0/inst/iterative_model_axi_top_v1_0_S00_AXI_inst/slv_reg1_reg_n_0_[2]_repN_6	6721	FDRE
iterative_model_v2_0_i/iterative_model_axi_0/inst/iterative_model_axi_top_v1_0_S00_AXI_inst/slv_reg1_reg_n_0_[3]_repN_6	6721	FDRE
iterative_model_v2_0_i/iterative_model_axi_0/inst/iterative_model_axi_top_v1_0_S00_AXI_inst/slv_reg1_reg_n_0_[4]_repN_6	6721	FDRE
iterative_model_v2_0_i/iterative_model_axi_0/inst/iterative_model_axi_top_v1_0_S00_AXI_inst/slv_reg1_reg_n_0_[5]_repN_6	6673	FDRE
iterative_model_v2_0_i/iterative_model_axi_0/inst/iterative_model_axi_top_v1_0_S00_AXI_inst/slv_reg1_reg_n_0_[0]_repN_2	6319	FDRE
iterative_model_v2_0_i/iterative_model_axi_0/inst/iterative_model_axi_top_v1_0_S00_AXI_inst/slv_reg1_reg_n_0_[1]_repN_2	6319	FDRE
iterative_model_v2_0_i/iterative_model_axi_0/inst/iterative_model_axi_top_v1_0_S00_AXI_inst/slv_reg1_reg_n_0_[2]_repN_1	6319	FDRE
iterative_model_v2_0_i/iterative_model_axi_0/inst/iterative_model_axi_top_v1_0_S00_AXI_inst/slv_reg1_reg_n_0_[3]_repN_2	6319	FDRE
iterative_model_v2_0_i/iterative_model_axi_0/inst/iterative_model_axi_top_v1_0_S00_AXI_inst/slv_reg1_reg_n_0_[4]_repN_1	6319	FDRE
iterative_model_v2_0_i/iterative_model_axi_0/inst/iterative_model_axi_top_v1_0_S00_AXI_inst/slv_reg1_reg_n_0_[5]_repN_1	6319	FDRE
iterative_model_v2_0_i/iterative_model_axi_0/inst/iterative_model_axi_top_v1_0_S00_AXI_inst/slv_reg1_reg_n_0_[0]	5919	FDRE
iterative_model_v2_0_i/iterative_model_axi_0/inst/iterative_model_axi_top_v1_0_S00_AXI_inst/slv_reg1_reg_n_0_[1]	5919	FDRE
iterative_model_v2_0_i/iterative_model_axi_0/inst/iterative_model_axi_top_v1_0_S00_AXI_inst/slv_reg1_reg_n_0_[2]	5919	FDRE
iterative_model_v2_0_i/iterative_model_axi_0/inst/iterative_model_axi_top_v1_0_S00_AXI_inst/slv_reg1_reg_n_0_[3]	5919	FDRE
iterative_model_v2_0_i/iterative_model_axi_0/inst/iterative_model_axi_top_v1_0_S00_AXI_inst/slv_reg1_reg_n_0_[4]	5919	FDRE
iterative_model_v2_0_i/iterative_model_axi_0/inst/iterative_model_axi_top_v1_0_S00_AXI_inst/slv_reg1_reg_n_0_[5]	5839	FDRE

Figure S30 – high-fanout nets report generated by Vivado. Here we present only few of the nets with the highest fanout.

In this report, we can see that all the high fanout nets where already replicated by the synthesis tool (due to the *repN_x* suffix. But still, after replicating those signals, the fanout is quite high. That is one of the reasons for the high congestion. To direct the Vivado tool chain to be more aggressive with the replication of those high fanout nets, we used the directive *AggressiveFanoutOpt.* This directive causes the implementation to use different algorithms for fanout related optimizations with more aggressive goals.

This change improved the congestion of the design but was not enough to allow the router to finish routing. The last change that was applied here was the separation of the design into two clock domains: the slow 100MHz clock domain for configuration and the fast 200MHz clock domain for the actual model. After splitting the design to those two clock domains, the congestion level was substantially reduced, and the router was able to finish routing the entire design.

Unfortunately, although the design was routed at this point, it did no meat the timing requirements. To fix that, we examined the critical paths that fail to meet the timing requirements. The first critical path was of the wide mux of the global arbiter as followed:

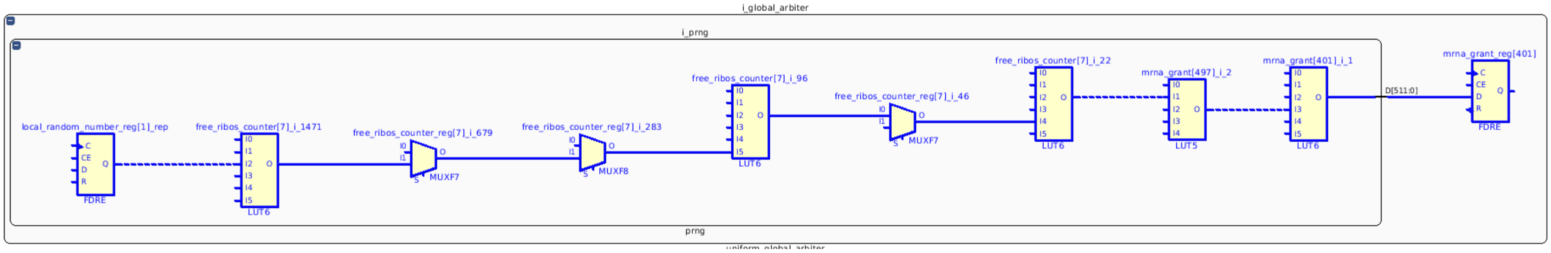

Figure S31- That was the critical path of the design. We can see that it is the demultiplexer used for the separate ribosome grant signal used for each mRNA molecule. That result lead to change of the interface of the arbiter to address based as explained in previous sections.

This critical path occurred due to the wide demultiplexer of the sperate grant signals that were connected to each mRNA molecule. To fix that, as mentioned, we changed the interface to and address based interface in which the arbiter provides the selected mRNA index to all mRNA and each mRNA molecules checks for itself.

After fixing that, we continued with place and route iterations in which continued failing in the timing report for different critical paths that are related to the wide multiplexers used in the arbiter. We changed those multiplexers to the pipelined multiplexers and that further improved the timing convergence.

The last change that was made it to pipeline the generation of the *hold* signal as explained in the iterative top module chapter. At this point the design met the setup requirements but had some small hold violations. Here is an example of such path that fails with a hold violation:

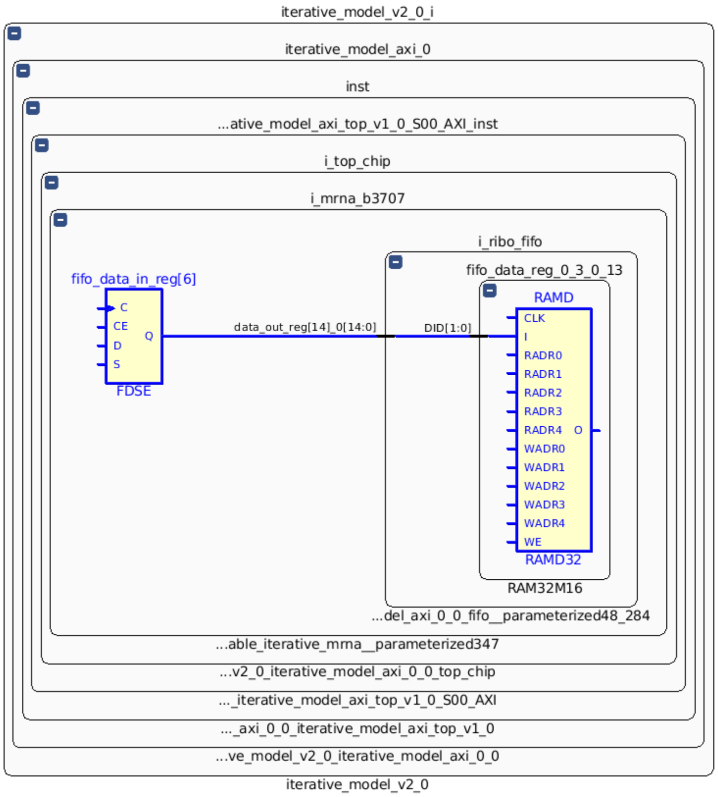

Figure S32 – one of the paths that are failing timing with hold violations. Logically it is just a wire. That means that those violations can be addressed with different router strategies to try and improving the wire's delay.

By viewing those paths, just as in the example, we can see that the paths that are failing the hold constraints are just wires that can possibly be routed differently. In order to fix that we used the directive *ExploreWithAggressiveHoldFix* which directs the router to run different algorithms in multiple passes of optimization, including aggressive hold violation fixing and replication of very high fanout nets. After applying this change, the design was finally routed and met all the timing constraints with positive slacks:

```
Phase 10 Post Router Timing

INFO: [Route 35-57] Estimated Timing Summary | WNS=0.336  | TNS=0.000  | WHS=0.010  | THS=0.000
```

This is taken from the router's log. It shows that after the 10th iteration of the router, the design finished with a positive setup slack of more than 0.336 for all nets. That basically means that we can try and improve the model clock frequency even further.

6 E.COLI DATA

6.1 CODON TRANSLATION DELAY

codon	Translation time in milliseconds (1ms resolution)
AAA	203
AAC	317
AAG	253
AAU	240
ACA	253
ACC	281
ACG	193
ACU	281
AGA	119
AGC	262
AGG	123
AGU	196
AUA	174
AUC	194
AUG	234
AUU	156
CAA	204
CAC	354
CAG	214
CAU	275
CCA	307
CCC	195
CCG	188
CCU	242
CGA	167
CGC	131
CGG	127
CGU	115
CUA	179
CUC	247
CUG	109
CUU	247
GAA	113

GAC	188
GAG	115
GAU	158
GCA	161
GCC	228
GCG	209
GCU	232
GGA	167
GGC	111
GGG	117
GGU	115
GUA	149
GUC	209
GUG	174
GUU	146
UAC	221
UAU	181
UCA	211
UCC	329
UCG	187
UCU	274
UGC	244
UGG	178
UGU	214
UUA	173
UUC	302
UUG	83
UUU	231

7 Appendix A – Xilinx Device Resources

Zynq®-7000 SoC Family

		Cost-Optimized Devices						Mid-Range Devices			
	Device Name	Z-7007S	Z-7012S	Z-7014S	Z-7010	Z-7015	Z-7020	Z-7030	Z-7035	Z-7045	Z-7100
	Part Number	XC7Z007S	XC7Z012S	XC7Z014S	XC7Z010	XC7Z015	XC7Z020	XC7Z030	XC7Z035	XC7Z045	XC7Z100
Processing System (PS)	Processor Core	Single-Core ARM® Cortex™-A9 MPCore™ Up to 766MHz			Dual-Core ARM Cortex-A9 MPCore Up to 866MHz			Dual-Core ARM Cortex-A9 MPCore Up to 1GHz[1]			
	Processor Extensions	NEON™ SIMD Engine and Single/Double Precision Floating Point Unit per processor									
	L1 Cache	32KB Instruction, 32KB Data per processor									
	L2 Cache	512KB									
	On-Chip Memory	256KB									
	External Memory Support[2]	DDR3, DDR3L, DDR2, LPDDR2									
	External Static Memory Support[2]	2x Quad-SPI, NAND, NOR									
	DMA Channels	8 (4 dedicated to PL)									
	Peripherals	2x UART, 2x CAN 2.0B, 2x I2C, 2x SPI, 4x 32b GPIO									
	Peripherals w/ built-in DMA[2]	2x USB 2.0 (OTG), 2x Tri-mode Gigabit Ethernet, 2x SD/SDIO									
	Security[3]	RSA Authentication of First Stage Boot Loader, AES and SHA 256b Decryption and Authentication for Secure Boot									
	Processing System to Programmable Logic Interface Ports (Primary Interfaces & Interrupts Only)	2x AXI 32b Master, 2x AXI 32b Slave<br>4x AXI 64b/32b Memory<br>AXI 64b ACP<br>16 Interrupts									
Programmable Logic (PL)	7 Series PL Equivalent	Artix®-7	Artix-7	Artix-7	Artix-7	Artix-7	Artix-7	Kintex®-7	Kintex-7	Kintex-7	Kintex-7
	Logic Cells	23K	55K	65K	28K	74K	85K	125K	275K	350K	444K
	Look-Up Tables (LUTs)	14,400	34,400	40,600	17,600	46,200	53,200	78,600	171,900	218,600	277,400
	Flip-Flops	28,800	68,800	81,200	35,200	92,400	106,400	157,200	343,800	437,200	554,800
	Total Block RAM	1.8Mb	2.5Mb	3.8Mb	2.1Mb	3.3Mb	4.9Mb	9.3Mb	17.6Mb	19.2Mb	26.5Mb
	(# 36Kb Blocks)	(50)	(72)	(107)	(60)	(95)	(140)	(265)	(500)	(545)	(755)
	DSP Slices	66	120	170	80	160	220	400	900	900	2,020
	PCI Express®	—	Gen2 x4	—	—	Gen2 x4	—	Gen2 x4	Gen2 x8	Gen2 x8	Gen2 x8
	Analog Mixed Signal (AMS) / XADC[2]	2x 12 bit, MSPS ADCs with up to 17 Differential Inputs									
	Security[3]	AES & SHA 256b Decryption & Authentication for Secure Programmable Logic Config									
	Speed Grades – Commercial	-1			-1			-1			-1
	Speed Grades – Extended	-2			-2,-3			-2,-3			-2
	Speed Grades – Industrial	-1, -2			-1, -2, -1L			-1, -2, -2L			-1, -2, -2L

Notes:
1. 1 GHz processor frequency is available only for -3 speed grades in Z-7030, Z-7035, and Z-7045 devices. See DS190, *Zynq-7000 SoC Overview* for details.
2. Z-7007S and Z-7010 in CLG225 have restrictions on PS peripherals, memory interfaces, and I/Os. Please refer to UG585, *Zynq-7000 SoC Technical Reference Manual* for more details.
3. Security block is shared by the Processing System and the Programmable Logic.

Page 2

© Copyright 2014–2019 Xilinx

XILINX

Zynq® UltraScale+™ MPSoCs: EV Devices

		Device Name[1]	ZU4EV	ZU5EV	ZU7EV
Processing System (PS)	Application Processor Unit	Processor Core	**Quad-core** ARM® Cortex™-A53 MPCore™ up to 1.5GHz		
		Memory w/ECC	L1 Cache 32KB I / D per core, L2 Cache 1MB, on-chip Memory 256KB		
	Real-Time Processor Unit	Processor Core	**Dual-core** ARM Cortex-R5 MPCore™ up to 600MHz		
		Memory w/ECC	L1 Cache 32KB I / D per core, Tightly Coupled Memory 128KB per core		
	Graphic & Video Acceleration	Graphics Processing Unit	Mali™-400 MP2 up to 667MHz		
		Memory	L2 Cache 64KB		
	External Memory	Dynamic Memory Interface	x32/x64: DDR4, LPDDR4, DDR3, DDR3L, LPDDR3 with ECC		
		Static Memory Interfaces	NAND, 2x Quad-SPI		
	Connectivity	High-Speed Connectivity	PCIe® Gen2 x4, 2x USB3.0, SATA 3.1, DisplayPort, 4x Tri-mode Gigabit Ethernet		
		General Connectivity	2xUSB 2.0, 2x SD/SDIO, 2x UART, 2x CAN 2.0B, 2x I2C, 2x SPI, 4x 32b GPIO		
	Integrated Block Functionality	Power Management	Full / Low / PL / Battery Power Domains		
		Security	RSA, AES, and SHA		
		AMS - System Monitor	10-bit, 1MSPS – Temperature and Voltage Monitor		
PS to PL Interface			12 x 32/64/128b AXI Ports		
Programmable Logic (PL)	Programmable Functionality	System Logic Cells (K)	192	256	504
		CLB Flip-Flops (K)	176	234	461
		CLB LUTs (K)	88	117	230
	Memory	Max. Distributed RAM (Mb)	2.6	3.5	6.2
		Total Block RAM (Mb)	4.5	5.1	11.0
		UltraRAM (Mb)	13.5	18.0	27.0
	Clocking	Clock Management Tiles (CMTs)	4	4	8
	Integrated IP	DSP Slices	728	1,248	1,728
		Video Codec Unit (VCU)	1	1	1
		PCI Express® Gen 3x16	2	2	2
		150G Interlaken	-	-	-
		100G Ethernet MAC/PCS w/RS-FEC	-	-	-
		AMS - System Monitor	1	1	1
	Transceivers	GTH 16.3Gb/s Transceivers	16	16	24
		GTY 32.75Gb/s Transceivers	-	-	-
	Speed Grades	Extended[2]	-1 -2 -2L -3		
		Industrial	-1 -1L -2		

Notes:
